# Network analysis of extraintestinal manifestations and associated autoimmune disorders in Crohn’s disease and ulcerative colitis

**DOI:** 10.1038/s41746-025-01504-6

**Published:** 2025-04-15

**Authors:** Daniel C. Baumgart, C. Hing Cheng, Tian X. Du, Michael D. Parkes, Daniel C. Sadowski, Eytan Wine, Frank Hoentjen, Brendan P. Halloran, Aldo Montano-Loza, Sergio Zepeda-Gomez, Karen Wong, Farhad Peerani, Randolph Goebel, J. Ross Mitchell

**Affiliations:** 1 Department of Gastroenterology and Hepatology, Charité Medical School, Humboldt-University of Berlin, Berlin, Germany; 2 College of Health Sciences, University of Alberta, Edmonton, AB Canada; 3 College of Natural and Applied Sciences, University of Alberta, Edmonton, AB Canada; 4 Alberta Health Services, Edmonton, AB Canada

**Keywords:** Inflammatory bowel disease, Computational science

## Abstract

We detect and interactively visualize occurrence, frequency, sequence, and clustering of extraintestinal manifestations (EIM) and associated immune disorders (AID) in 30,334 inflammatory bowel disease (IBD) patients (Crohn’s disease (CD) *n* = 15924, ulcerative colitis (UC) *n* = 11718, IBD unclassified, IBD-U *n* = 2692, 52% female, median age 40 years (IQR: 25)) with artificial intelligence (AI). 57% (CD > UC 60% vs. 54%, *p* < 0.00001) had one or more EIM and/or AID. Mental, musculoskeletal and genitourinary disorders were most frequently associated with IBD: 18% (CD vs. UC 19% vs. 16%, *p* < 0.00001), 17% (CD vs. UC 20% vs. 15%, *p* < 0.00001) and 11% (CD vs. UC 13% vs. 9%, *p* < 0.00001), respectively. AI detected 4 vs. 5 vs. 5 distinct EIM/AID communities with 420 vs. 396 vs. 467 nodes and 11,492 vs. 9116 vs. 16,807 edges (links) in CD vs. UC vs. IBD, respectively. Our newly developed interactive free web app shows previously unknown communities, relationships, and temporal patterns—the diseasome and interactome.

## Introduction

Chronic inflammatory bowel diseases (IBD)^1^, i.e. Crohn’s disease (CD)^2^ and ulcerative colitis (UC)^3^, result from an inappropriate immune response towards the commensal microbiota^4^ in genetically^5^ susceptible individuals, exacerbated and promoted by environmental factors such as Western lifestyle, diet and industrialization. They cannot be cured and require lifelong medical therapy^6^.

IBD affects mainly the digestive tract including the liver^7^, but due to their systemic nature, they can involve virtually all parts of the human body through extraintestinal manifestations (EIM) and associated autoimmune diseases (AID)^8,9^. Previously^10,11^, research on extraintestinal manifestations and associated autoimmune disorders in IBD was based on non-population representative cohorts mostly from academic tertiary referral centers with roughly 1000 patients based on self-reporting.

Here we apply—for the first time—the concept of network medicine to IBD^12^. By mapping network-based dependencies between diseases a concept of an unbiased IBD diseasome evolves, with color-coded disease maps whose nodes are diseases and whose links represent various relationships between CD and UC associated EIM and AID. We report and digitally visualize the frequency and relationship of EIM and AID to the underlying disease (CD vs. UC vs. IBD) and among each other at different organization levels.

Our study is the most comprehensive, first interactive, and first artificial intelligence-supported analysis of extraintestinal manifestations and associated autoimmune disorders in IBD based on the largest, most diverse, population-representative cohort to date including 30 times more patients than previous studies, spanning nearly two decades, identifying disease clusters and networks and thereby introducing the concept of network medicine into IBD.

Through our interactive models (web app available in the online supplement) we hope to assist clinicians in their daily practice by recognizing relationships between individual and clustered EIM and AID and optimizing clinical decision-making on their management and advance research in this field.

## Results

### Patients

#### IBD patient cohort extraction

We initially identified individual patients who were diagnosed at least once with IBD, either CD or UC as described above in a cohort of up to 18 years spanning from 2002 to 2020. After application of the previously validated definition^13^, 30,334 IBD (CD *n* = 15,924, UC *n* = 11,718, IBD unclassified, IBD-U *n* = 2692) patients remained for evaluation (Table 1).

**Table 1:** Basic demographic characterization of the patient population

Disease type		Crohn’s disease		Ulcerative colitis		IBDU		IBD Total(CD + UC + IBDU)		UC vs. CD
		**Total** ***N*** = **15,924**		**Total** ***N*** = **11,718**		**Total** ***N*** = **2692**		**Total** ***N*** = **30,334**		***p*** **value** ≤
**Sex**		**14,806**		**10,476**		**1663**		**26,945**		
		***n***	**%**	***n***	**%**	***n***	**%**	***n***	**%**	
Male		6805	45.961	5401	51.556	828	49.790	13,034	48.373	0.00001
Female		8001	54.039	5075	48.444	835	50.210	13,911	51.627	0.00001
**AGE [years]**		**14,806**		**10,476**		**1663**		**26,945**		
Age (at first encounter)		**Median**	**IQR**	**Median**	**IQR**	**Median**	**IQR**	**Median**	**IQR**	
		40	25.000	40	25.000	44	27.000	40	25.000	0.00001
Age groups (at first encounter)		***n***	**%**	***n***	**%**	***n***	**%**	***n***	**%**	
Children and adolescents [<18 years]		1133	7.652	651	6.214	59	3.548	1843	6.840	0.00001
Adults [18–64 years]		12,471	84.229	8784	83.849	1354	81.419	22,609	83.908	0.4250
Seniors [>64 years]		1202	8.118	1041	9.937	250	15.033	2493	9.252	0.00001
*Disease features and complications*										
Fistulas										
Fissure and fistula of anal and rectal regions	K60	0	0.000	0	0.000	0	0.000	0	0.000	0.00001
Acute anal fissure	K60.0	55	0.345	24	0.205	5	0.186	84	0.277	0.0404
Chronic anal fissure	K60.1	78	0.490	31	0.265	9	0.334	118	0.389	0.0043
Anal fissure. unspecified	K60.2	909	5.708	400	3.414	99	3.678	1408	4.642	0.00001
Anal fistula	K60.3	1342	8.428	180	1.536	80	2.972	1602	5.281	0.00001
Rectal fistula	K60.4	258	1.620	19	0.162	10	0.371	287	0.946	0.00001
Anorectal fistula	K60.5	80	0.502	10	0.085	7	0.260	97	0.320	0.00001
Fistula of intestine	K62.3	61	0.383	68	0.580	18	0.669	147	0.485	0.0221
Fistulae involving female genital tract	N82	0	0.000	0	0.000	0	0.000	0	0.000	0.00001
Fistula of vagina to small intestine	N82.2	15	0.094	4	0.034	4	0.149	23	0.076	0.0988
Fistula of vagina to large intestine	N82.3	251	1.576	32	0.273	13	0.483	296	0.976	0.00001
Other female intestinal-genital tract fistula.	N82.4	57	0.358	11	0.094	7	0.260	75	0.247	0.00001
Vesicointestinal fistula	K82.3	4	0.025	1	0.009	0	0.000	5	0.016	0.5750
Number of patients with Fistulas		2350	14.758	675	5.760	206	7.652	3231	10.651	0.00001
Abscesses										
Abscess of anal and rectal regions	K61	0	0.000	0	0.000	0	0.000	0	0.000	0.00001
Anal abscess	K61.0	1246	7.825	225	1.920	75	2.786	1546	5.097	0.00001
Rectal abscess	K61.1	485	3.046	97	0.828	35	1.300	617	2.034	0.00001
Anorectal abscess	K61.2	119	0.747	26	0.222	9	0.334	154	0.508	0.00001
Ischiorectal abscess	K61.3	484	3.039	93	0.794	30	1.114	607	2.001	0.00001
Number of patients with Abscesses		1496	9.395	308	2.628	101	3.752	1905	6.280	0.00001
Obstructive complications										
Paralytic ileus and intestinal obstruction without hernia	K56	0	0.000	0	0.000	0	0.000	0	0.000	0.00001
Paralytic ileus	K56.0	59	0.371	25	0.213	10	0.371	94	0.310	0.0254
Intussusception	K56.1	39	0.245	15	0.128	6	0.223	60	0.198	0.0416
Volvulus	K56.2	98	0.615	47	0.401	14	0.520	159	0.524	0.0186
Other impaction of intestine	K56.4	81	0.509	31	0.265	12	0.446	124	0.409	0.0022
Intestinal adhesions [bands] with obstruction	K56.5	759	4.766	198	1.690	55	2.043	1012	3.336	0.00001
Other and unspecified intestinal obstruction	K56.6	6306	39.601	1613	13.765	374	13.893	8293	27.339	0.00001
Ileus. unspecified	K56.7	590	3.705	232	1.980	80	2.972	902	2.974	0.00001
Number of patients with obstructive complications		6586	41.359	1804	15.395	438	16.270	8828	29.103	0.00001
*Medication exposure*	ATC-CODES									
Conventional therapy										
5-ASA (mesalazine/mesalamine)	A07EC02	6450	40.505	9241	78.862	1348	50.074	17039	56.171	0.00001
SASP (sulfasalazine) PO	A07EC01	635	3.988	549	4.685	88	3.269	1272	4.193	0.0051
Number of patients on conventional therapy		6782	42.590	9373	79.988	1385	51.449	17540	57.823	0.00001
Immunomodulators (antimetabolites)										
Azathioprine	L04AX01	6353	39.896	2867	24.467	297	11.033	9517	31.374	0.00001
6-Mercaptopurine	L01BB02	351	2.204	136	1.161	10	0.371	497	1.638	0.00001
Methotrexate	L01BA01	2577	16.183	759	6.477	147	5.461	3483	11.482	0.00001
Methotrexate	L04AX03	80	0.502	28	0.239	9	0.334	117	0.386	0.0007
Number of patients on immunomodulators		8023	50.383	3353	28.614	419	15.565	11795	38.884	0.00001
Calcineurin inhibitors										
Tacrolimus	D11AH01	649	4.076	389	3.320	68	2.526	1106	3.646	0.0012
Tacrolimus	L04AD02	77	0.484	78	0.666	23	0.854	178	0.587	0.0546
Cyclosporine	L04AD01	69	0.433	54	0.461	12	0.446	135	0.445	0.8039
Cyclosporine	S01XA18	156	0.980	124	1.058	24	0.892	304	1.002	0.5594
Number of patients on calcineurin inhibitors		911	5.721	617	5.265	115	4.272	1643	5.416	0.1072
Steroids										
Prednisone	H02AB07	9152	57.473	6949	59.302	1174	43.611	17275	56.949	0.0024
Prednisolone	H02AB06	73	0.458	55	0.469	5	0.186	133	0.438	0.9659
Prednisolone	S01BA04	1466	9.206	1085	9.259	261	9.695	2812	9.270	0.8970
methylprednisolone	H02AB04	824	5.175	612	5.223	139	5.163	1575	5.192	0.8801
Budesonide	A07EA06	3960	24.868	1835	15.660	382	14.190	6177	20.363	0.00001
Hydrocortisone	A07EA02	750	4.710	2747	23.443	183	6.798	3680	12.132	0.00001
Hydrocortisone	D07AA02	3097	19.449	2149	18.339	504	18.722	5750	18.956	0.0210
Hydrocortisone	D07XA01	139	0.873	102	0.870	20	0.743	261	0.860	0.9650
Hydrocortisone	H02AB09	2143	13.458	982	8.380	115	4.272	3240	10.681	0.00001
Number of patients on Steroids		12,162	76.375	8914	76.071	1745	64.822	22821	75.232	0.5665
JAK inhibitors										
Tofacitinib	L04AA29	21	0.132	135	1.152	10	0.371	166	0.547	0.00001
Biologics										
Anti-TNF										
Infliximab	L04AB02	4179	26.243	2049	17.486	190	7.058	6418	21.158	0.00001
Adalimumab	L04AB04	3919	24.611	988	8.431	136	5.052	5043	16.625	0.00001
Golimumab	L04AB06	133	0.835	168	1.434	19	0.706	320	1.055	0.00001
Certolizumab pegol	L04AB05	19	0.119	6	0.051	3	0.111	28	0.092	0.0971
Anti-IL12/23										
Ustekinumab	L04AC05	1617	10.154	184	1.570	41	1.523	1842	6.072	0.00001
Anti-integrin										
Vedolizumab	L04AA33	533	3.347	596	5.086	39	1.449	1168	3.850	0.00001
Number of patients on Biologics		7667	48.147	3081	26.293	332	12.333	11080	36.527	0.00001
*Healthcare utilization*										
Average number of hospitalizations per patient per year		0.231851294		0.185782557		0.20690936		0.2118415		0.000001
Average number of ER visits per patient per year		3.902599849		2.971070148		3.12667162		3.47389068		0.00001
Average number of hospitalizations & ER visits per patient per year		4.134451143		3.156852705		3.33358098		3.68573218		0.00001

#### IBD patient cohort characterization

The studied cohort included equal numbers of female (51.6%) and male (48.4%) mostly adult (83.9%) patients aged between 18 and 64 years, aged 40 ± 25 (median, IQR) years. CD was as expected statistically significantly more often complicated by obstructive problems, fistulas, and abscesses. Patients’ medical treatment history was typical for IBD, with CD patients statistically significantly more often exposed to antimetabolites, anti-TNFα, and anti-IL-12/23 biologics, compared with ulcerative colitis patients who were, in line with professional treatment guidelines, prescribed aminosalicylates, calcineurin inhibitors, and anti-integrins more often. Healthcare utilization among CD patients was higher with per patient per year compared with UC, respectively. Patient features are further detailed in Table 1.

#### Frequency of extraintestinal manifestations and associated autoimmune disorders

The Canadian version of official WHO ICD code domains was followed to group the data for comparability and secondary data use with our above-described selection criteria, i.e. not ICD codes (comorbidities) were included but those that related to EIM and AID (Table 2 Web Tables 1–14). More than half of all IBD patients experience EIM or AID. EIM and AID occur significantly more frequently in CD (60%) vs. UC (54%) (*p* < 0.00001) (Table 2).

**Table 2:** Summary of frequency and comparison of extraintestinal disease manifestations

Organ system (sorted by frequency)	Crohn’s disease		Ulcerative colitis		IBD-unclassified		IBD-Total (CD + UC + IBDU)		Comparison
	*N* = 15,924		*N* = 11,718		*N* = 2692		*N* = 30,334		UC vs. CD
	*n*		*n*		*n*		*n*		*p* value
		%		%		%		%	
Mental behavioral and neurodevelopmental disorders	3095	19.436	1869	15.950	537	19.948	5501	18.135	0.00001
Diseases of the musculoskeletal system and connective tissue	3142	19.731	1767	15.079	467	17.348	5376	17.723	0.00001
Diseases of the genitourinary system	2046	12.849	1106	9.438	279	10.364	3431	11.311	0.00001
Cerebrovascular diseases	1657	10.406	1188	10.138	310	11.516	3155	10.401	0.4820
Diseases of the circulatory system	1584	9.947	1240	10.582	322	11.961	3146	10.371	0.0888
Diseases of the respiratory system	1658	10.412	1092	9.319	276	10.253	3026	9.976	0.0029
Symptoms, signs and abnormal clinical and laboratory findings, not elsewhere classified	1415	8.886	916	7.817	275	10.215	2606	8.591	0.0017
Diseases of the digestive system	1209	7.592	968	8.261	290	10.773	2467	8.133	0.0438
Diseases of the blood and blood-forming organs and certain disorders involving the immune mechanism	865	5.432	461	3.934	129	4.792	1455	4.797	0.00001
Diseases of the skin and subcutaneous tissue	794	4.986	381	3.251	89	3.306	1264	4.167	0.00001
Diseases of the nervous system	640	4.019	472	4.028	122	4.532	1234	4.068	0.9950
Endocrine. nutritional and metabolic diseases	327	2.054	286	2.441	94	3.492	707	2.331	0.0341
Diseases of the eye and adnexa	458	2.876	212	1.809	37	1.374	707	2.331	0.00001
Diseases of the ear and mastoid process	244	1.532	215	1.835	48	1.783	507	1.671	0.0578
Number of patients with any EIM & AID	9490	59.596	6290	53.678	1522	56.538	17302	57.038	0.00001

Please see the additional 14 web tables detailing all organ systems in the online supplemental material and the web software application.

#### Mental and musculoskeletal conditions are the most common EIM and AID in IBD

Mental, behavioral, and neurodevelopmental disorders (IBD 18%, CD vs. UC 19% vs. 16%, *p* < 0.00001) as well as diseases of the musculoskeletal system and connective tissue are most frequently associated with IBD (IBD 17%, CD vs. UC 20% vs. 15%, *p* < 0.00001). Table 2 Among the mental disorders depression and anxiety dominate. Supplementary Table 1 Among the musculoskeletal disorders, arthropathies, ankylosing spondylitis, and myalgia dominate (Supplementary Table 2).

#### Genitourinary, cerebrovascular, circulatory, respiratory, and digestive EIM and AID are less frequently associated with IBD

Overall, Genitourinary conditions are more frequently associated with Crohn’s disease (IBD 11%, CD vs. UC 13% vs. 9%, *p* < 0.00001) (Table 2). Calculus of the kidney, ureter, and bladder dominate and occur more frequently in UC, while tubulo-interstitial nephritis is more commonly seen in CD (Supplementary Table 3). Cerebrovascular diseases are associated with 10% of IBD patients with no preference for CD or UC (10% vs. 10%, *p* = 0.48) (Table 2). Among the cerebrovascular disorders, phlebitis and thrombophlebitis, embolism, and thrombosis and stroke dominate (Supplementary Table 4). Circulatory system diseases are associated with 10% of IBD patients with no preference for CD or UC (10% vs. 10%, *p* = 0.08) (Table 2). Among the circulatory disorders, cardiac ischemia and pulmonary embolism dominate (Supplementary Table 5). Respiratory system diseases are associated with 10% of IBD patients and more often in CD vs. UC (10% vs. 9%, *p* = 0.029) (Table 2). Among the respiratory disorders asthma dominates by far and occurs significantly more frequently in CD vs. UC (Supplementary Table 6).

#### General, digestive, hematological, dermatological, neurological, endocrine, ophthalmic, and otolaryngeal EIM and AID are least frequently associated with IBD

General symptoms signs and abnormal clinical and laboratory findings not elsewhere classified are more frequently associated with CD (CD vs. UC 9% vs. 8%, *p* = 0.0017) (Table 2). Malaise and fatigue dominate in this category (Supplementary Table 7). Digestive disorders (other than the underlying conditions CD and UC) are more frequently associated with UC (UC vs. CD 7% vs. 8%, *p* = 0.04). Table 2 Celiac disease and autoimmune liver diseases dominate here (Supplementary Table 8). Diseases of the blood and blood-forming organs and certain disorders involving the immune system are more frequently associated with CD (CD vs. UC (5% vs. 3%, *p* < 0.000001)) (Table 2). Anemia, coagulation defects, immunodeficiencies, and immune diseases dominate here (Supplementary Table 9). Diseases of the skin and subcutaneous tissue are more frequently associated with CD (CD vs. UC (5% vs. 3%, *p* < 0.000001)). Table 2. Psoriasis, pyoderma, and erythema nodosum dominate here (Supplementary Table 10). Diseases of the nervous system are equally associated with IBD (CD vs. UC (3% vs. 3%, *p* = 0.99)). Transient cerebral ischemia and multiple sclerosis dominate here (Supplementary Table 11). Endocrine, nutritional and metabolic diseases are slightly more frequently associated with UC (UC vs. CD 2% vs. 2%, *p* = 0.03). Type 1 diabetes dominates here (Supplementary Table 12). Diseases of the eye and adnexa are more frequently associated with CD (CD vs. UC (3% vs. 2%, *p* < 0.000001) (Table 2). Iridocyclitis, episcleritis and scleritis dominate here (Supplementary Table 13). Diseases of the ear and mastoid process are least frequently associated with IBD and sensorineural hearing loss (Supplementary Table 14) dominate among the least frequent EIM and AID in IBD (Table 2).

### Clustering of EIM and AID—community detection network analysis

**Fig. 1:**
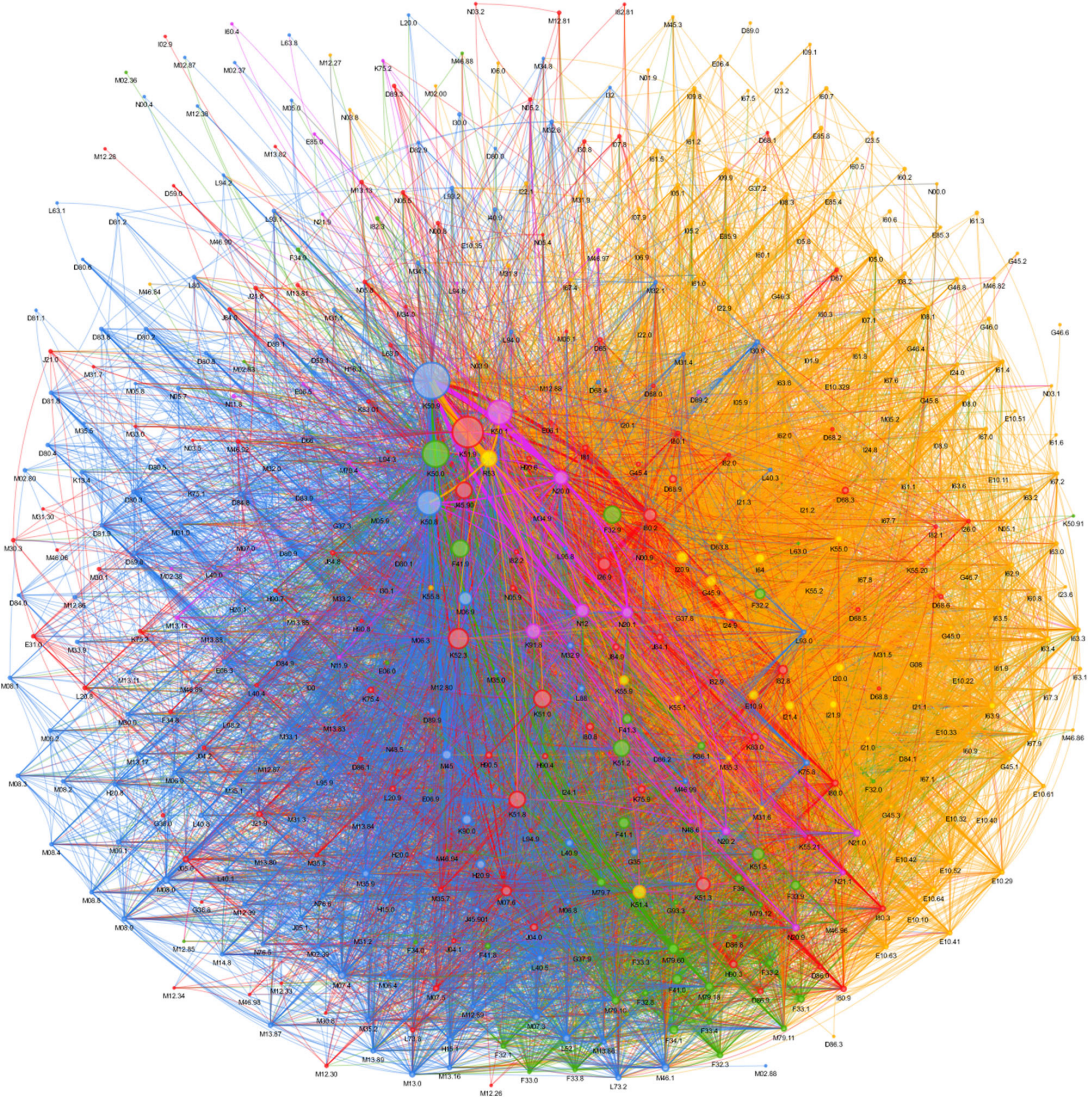
Louvain community network detection analysis of extraintestinal manifestations in inflammatory bowel disease. Most EIM and AID associated with IBD occur in two large clusters that appear in blue and yellow, three smaller red, green, and purple clusters. Click here for the interactive version Interactive Figure 1.

**Fig. 2:**
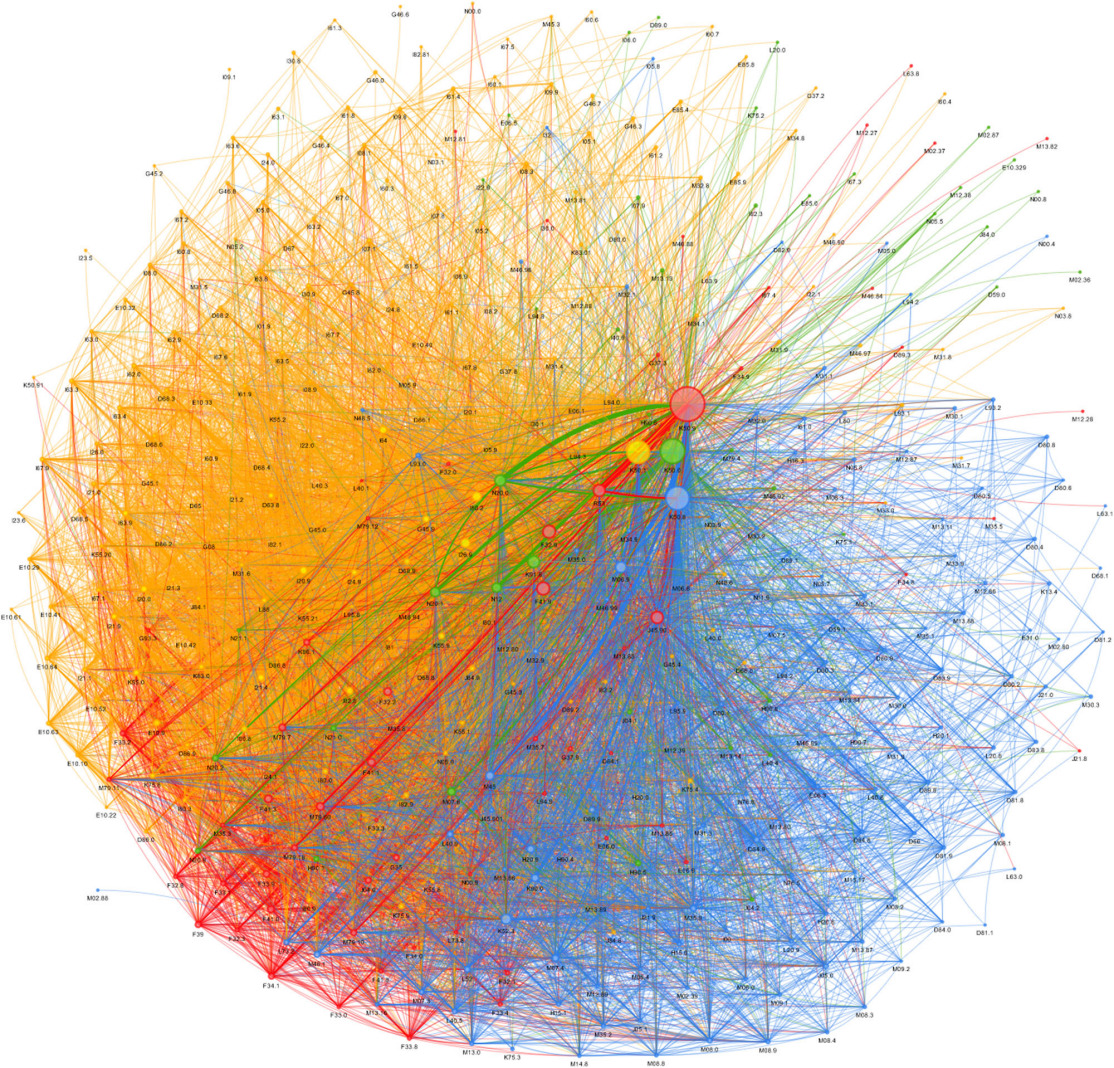
Louvain Community Network Detection Analysis of Extraintestinal Manifestations in Crohn’s Disease. Most EIM and AID associated with CD occur in two large clusters that appear in blue and yellow and two smaller green and red clusters. Click here for the interactive version Interactive Figure 2.

**Fig. 3:**
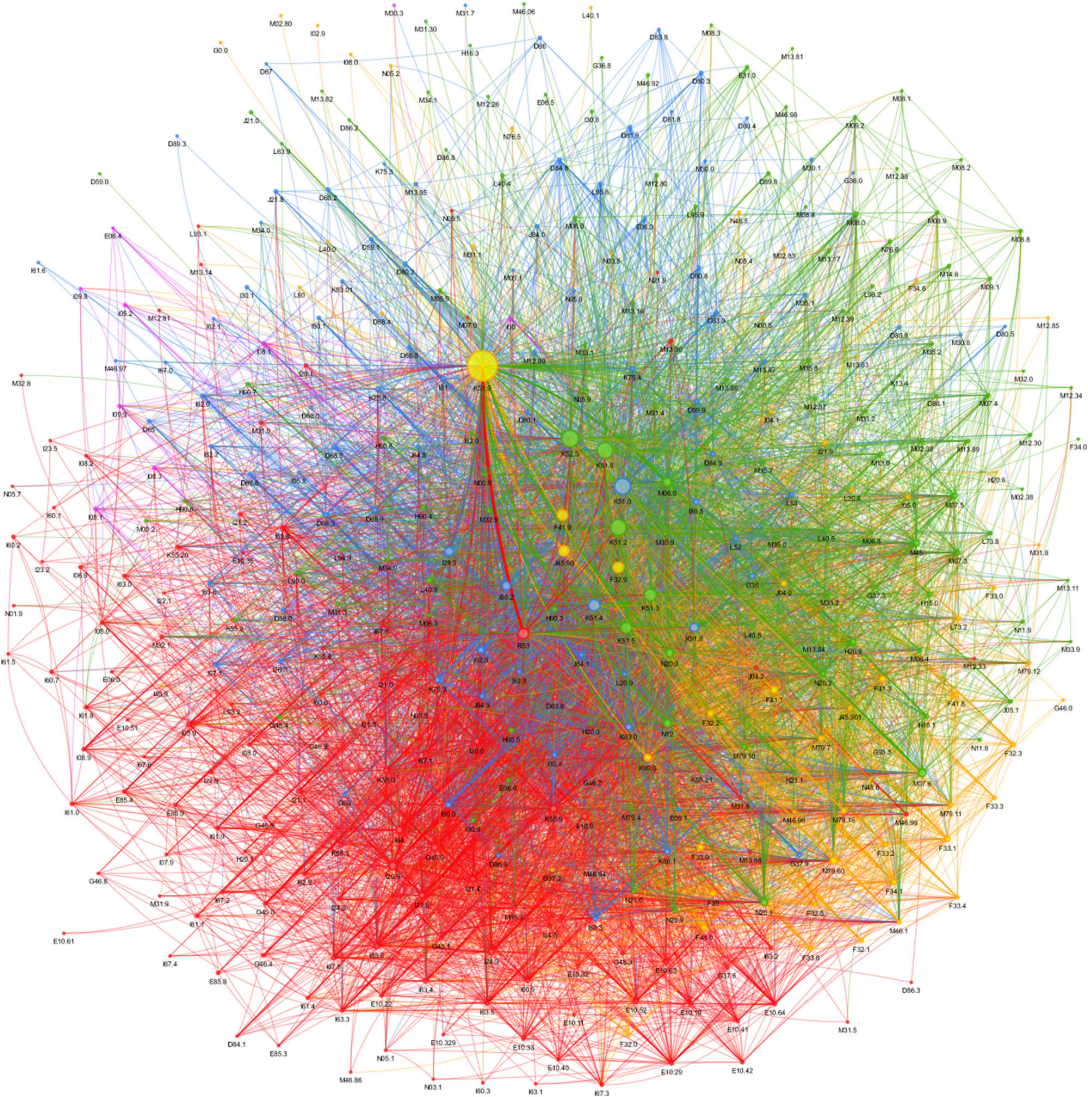
Louvain community network detection analysis of extraintestinal manifestations in ulcerative colitis. Most EIM and AID associated with UC occur in two large clusters that appear in green and red, two smaller red and blue clusters, and one very small purple cluster. Click here for the interactive version Interactive Figure 3 in the online supplement.

Below we are summarizing the main clusters and associations of EIM and AID in the static printed figures. Many more clusters, networks and relationships can be explored by individually interrogating our interactive dataset. This can be accomplished either by clicking a) on any *node* of the network to highlight the associated *edges* (links) and associated *nodes* b) dragging the clicked nodes around to rearrange them on the online Interactive Fig. 1, Interactive Fig. 2, and Interactive Fig. 3 or more systematically including additional statistics and tables with our Interactive App.

#### Louvain network analysis identifies two large and three smaller distinct EIM and AID clusters in IBD

Most EIM and AID associated with IBD occur in two large clusters that appear in blue and yellow, three smaller red, green, and purple clusters (Fig. 1, Interactive Fig. 1).

In the blue cluster the central, largest node depicts unspecified CD (K50.9) connected to a smaller blue node depicting CD of the small and large intestine (K50.8). They form a tightly woven network of links (*edges*) to rheumatoid arthritis (M06.9) and most other musculoskeletal and connective tissue conditions.

The largest *node* in the yellow cluster is malaise and fatigue (R53). This is most closely related to (thick link/edge) unspecified CD (K50.8) much lesser degree to ulcerative proctitis (K51.2) and a tight network (many links/*edges*) of distinct endocrine, cerebrovascular, circulatory, neurological and some musculoskeletal EIM and AID.

The most significant *nodes* in the smaller green cluster are CD of the small intestine (K50.0) and to a lesser degree ulcerative proctitis (K51.2) and left-sided colitis (K51.5) with dominant clustering of depression (F32.9), panic disorder (F41), anxiety (F41.4), and limb pain (M79.60).

The smaller purple cluster evolves around the CD of the large intestine (K50.1) with strong relationships to calculus of the kidney (N20.0) and ureter (N20.1), tubulo-interstitial nephritis (N12) but also intraoperative and postprocedural complications of digestive disorders (K91.8).

The smaller red cluster centers around ulcerative colitis (K51.9) with tight woven networks (links/*edges*) to phlebitis and thrombophlebitis of other and unspecified deep vessels of the lower extremities (I80.2), pulmonary embolism without acute Cor pulmonale (I26.9), and embolism and thrombosis of other specified veins (I82.2).

#### Network analysis identifies two large and two smaller distinct EIM and AID clusters in CD

Most EIM and AID associated with CD occur in two large clusters that appear in blue and yellow and two smaller green and red clusters (Fig. 2, Interactive Fig. 2).

The yellow cluster is focused on its largest *node*, CD of the large intestine (K50.1) and is connected to smaller yellow *nodes* representing VTE (venous thromboembolic events) and MACE (major cardiovascular events) like phlebitis and thrombophlebitis of other and unspecified deep vessels of the lower extremities (I80.2), pulmonary embolism without acute Cor pulmonale (I26.9), transient ischemic attacks (G45.9), Angina pectoris (I20.9), non-ST-elevation myocardial infarction (I21.4) (NSTEMI), vascular disorders of the intestine (K55.9) and a tightly woven network of distinct mostly cerebrovascular, circulatory, endocrine EIM and AID.

The blue cluster is focused on its largest *node*, CD of both small and large intestine (K50.9), and smaller blue nodes like unspecified rheumatoid arthritis (M06.9), ankylosing spondylitis (M45.9), celiac disease (K90.0) and unspecified iridocyclitis (H20.9), immunodeficiency (D84.9) and tightly woven network of distinct, mostly musculoskeletal, and some dermatological EIM and AID.

The smaller green cluster is centered around the CD of the small intestine (K50.0) with larger green sub-nodes for kidney (N20.0), ureter calculus (N20.1), tubule-interstitial nephritis (N12), but also intraoperative and procedural complications of digestive disorders (K91.8).

The smaller red cluster is centered around unspecified CD (K50.9) is strongly associated with mental disorder red *sub-nodes* like malaise and fatigue (R53) and major depressive disorder (F32.9) and anxiety (F41.9) but also asthma unspecified (J45.90) and limb pain (M79.60) as well as a tightly woven network of mostly mental EIM and AID.

#### Network analysis identifies two large and two smaller distinct EIM and AID clusters in UC

Most EIM and AID associated with UC occur in two large clusters that appear in green and red, two smaller red and blue clusters, and one very small purple cluster. (Fig. 3, Interactive Fig. 3).

The main node series in the green cluster centers around other ulcerative colitis (K51.8), ulcerative proctitis (K51.2), ulcerative rectosigmoiditis (K51.3), left-sided UC (K51.5) but also indeterminate colitis (K52.3) associated with kidney (N20.0) calculus (N20.1) and tubulo-interstitial nephritis (N12) and a tightly woven network of distinct mostly musculoskeletal and dermatological EIM and AID.

The main node in the red cluster is malaise and fatigue (R53), which is strongly connected (thick *edge*) to the smaller yellow cluster of unspecified UC (K51.9) and a tightly woven network venous thromboembolic events (VTE) and major cardiovascular events (MACE), like phlebitis and thrombophlebitis of other and unspecified deep vessels of the lower extremities (I80.2), pulmonary embolism without acute Cor pulmonale (I26.9), transient ischemic attacks (G45.9), angina pectoris (I20.9), non-ST-elevation myocardial infarction (NSTEMI) (I21.4) and vascular disorders of the intestine (K55.9).

The smaller yellow cluster is centered around unspecified UC (K51.9) and strong associations with mental disorders such as anxiety (F41.9), major depression (M32.9), generalized- (F41.1), and mixed anxiety disorders (F41.3) as well as asthma (J45.90), but also acute laryngitis (J04.0) and celiac disease (K90.0).

The smaller blue cluster is centered around pancolitis (K51.0) and strongly associated with inflammatory polyps (K51.4) and various VTE / MACE disorders like phlebitis and thrombophlebitis of other and unspecified deep vessels of the lower extremities (I80.2), pulmonary embolism without acute Cor pulmonale (I26.9), angina pectoris (I20.9), non-ST-elevation (I21.4) and vascular disorders of the intestine (K55.9).

One very small purple cluster evolves around rheumatic heart conditions (I00).

#### Algorithm detected EIM and AID communities are not random

**Fig. 4:**
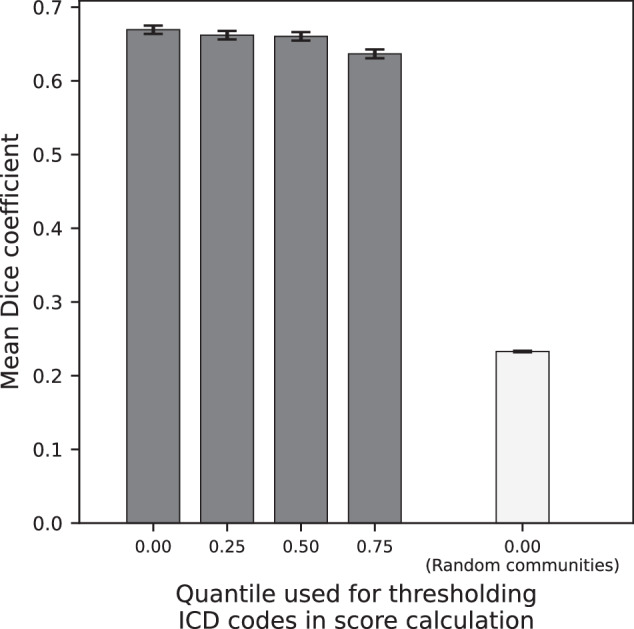
EIM and AID community overlap score by shuffling ICD co-occurrence edge list. Error bars represent SEM. The lightly colored bar gives the mean community overlap score for 1000 pairs of randomly generated communities. The *x*-axis values indicate the quantile of ICD frequencies in the patient population that was used to threshold the ICD codes that were included in the overlap score calculation. ICD codes whose frequencies in the patient population fell in the quantiles stated along the *x*-axis were included in the overlap score calculation.

**Fig. 5:**
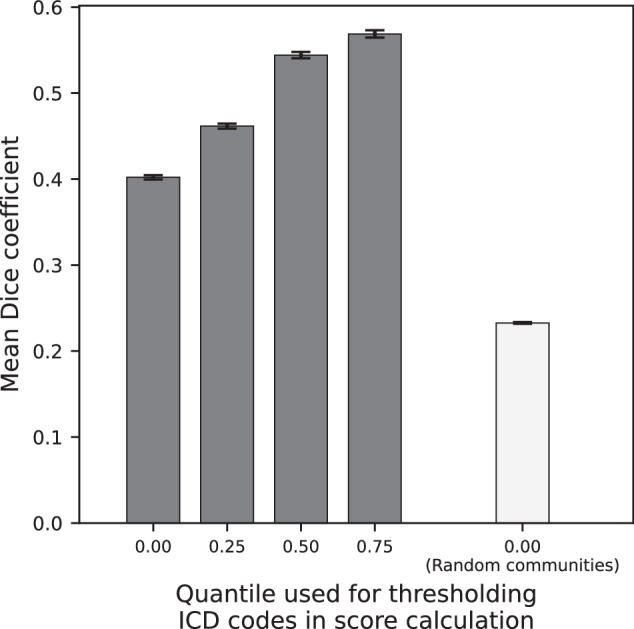
EIM and AID community overlap score on random disjoint samples of patients. Error bars represent SEM. The lightly colored bar gives the mean community overlap score for 1000 pairs of randomly generated communities. The *x*-axis values indicate the quantile of ICD frequencies in the patient population that was used to threshold the ICD codes that were included in the overlap score calculation. ICD codes whose frequencies in the patient population fell in the quantiles stated along the *x*-axis were included in the overlap score calculation.

The mean overlap score (Dice Sorensen coefficient) for randomly generated communities was 0.232 ± 0.001 SEM. Figure 4 illustrates the expected community overlap variability driven by the order in which ICD code co-occurrences are input to the Louvain algorithm. Figure 5 illustrates the overlap variability is driven by patient sampling differences. The differences between the quartile-restricted scores were pronounced when the source of variability was patient sampling. The most frequently observed ICD codes form the most consistent community association across trials, supporting the notion that the algorithm-detected communities are based on a stable “core” set of ICD codes. Irrespective of whether the source of variability was algorithmic or patient sampling differences, the mean overlap scores using real ICD co-occurrence data were much better than when the communities were randomly assigned (one-sided Welch’s *t*-test *p* < 0.001).

#### Further network interactive disease network exploration

While the main goal of our study was to discover and report disease clusters within the main network categories IBD, CD, and UC, the data can be explored further with our newly developed web-based Interactive App accessible in any web browser.

By default (i.e. without any additional user input in the web browser) IBD is visualized in cluster mode including all WHO ICD disease domains (hierarchies) and without any code pair restrictions, i.e. even if only one code association exists, it will be included. This default setting allows for a comprehensive overview.

However, the user may want to explore specific aspects of the data in a research or clinical setting. Several visualization options are available to choose from in the left panel. First, one of the three disease networks (IBD, CD, or UC) can be selected. Second, the network organization can be grouped by clusters (default) or the WHO ICD hierarchy (essentially organ system domains). As discussed in the methods section above, in cluster mode discovered disease clusters are grouped by the same color, while in ICD mode WHO disease domains are grouped by the same color. Third, the number of WHO disease domains to be considered (i.e. hierarchical organization) can be reduced from 7 (default) to 1. A reduced number of WHO disease domains allows for the identification of the strongest clusters. Lastly, the number of ICD pairs can be varied from (default) to 1000. A lower number of IVD code pairs considers even very rare associations, while a higher number focuses on the most frequent associations in a cluster.

## Discussion

To the best of our knowledge, this is the most comprehensive, first interactive, and first artificial intelligence^14–16^ supported analysis of extraintestinal manifestations and associated autoimmune disorders in IBD. Our results are based on the largest cohort to date (i.e. >30 times more patients than the last study, published more than a decade ago)^11^. Our work goes beyond reporting frequencies and associated classic statistics as we have identified and visualized disease clusters and networks in IBD using a novel methodology.

The concept of network medicine, i.e. a network-based approach to the understanding of complex human diseases originated in genetics^12^, a natural data-rich branch of biomedical research that adopted computational methodology early on and successfully uses it until this very day^17^. Previously, computational molecular genetics was used to discover gene interactions^18^, and disease modules^19^, identify disease pathways^5^, and predict other disease genes to develop new therapeutic concepts.

The increasing availability of population-representative large electronic health record-based data sets^20^ enables the application of the network medicine concept to such sufficiently deep, rich, and structured resources and drives unbiased discovery from the clinical setting including hypothesis generation for molecular research. Our study demonstrates the highly interconnected nature, the *interactome*, of IBD and its respective EIM and AID. It validates and expands previously established molecular relationships with nearly two decades of big data from electronic health records between various chronic systemic inflammatory conditions^8^. It shows in previously not possible ways that these diseases cannot be considered independent of one another. The mapping of network-based dependencies has culminated in the concept of the “diseasome”, which represents disease maps whose nodes are diseases and whose links represent various relationships between them. Our interactive Web App allows clinicians to uncover possibly associated EIM and AIDS in IBD and apply these insights to their respective patients. This advanced knowledge is another important step towards precision medicine and helps guide therapy in a way that addresses all medical needs.

Our results align with the efforts of large international expert panels to take a much broader look at extraintestinal manifestations and associated autoimmune disorders^8,9^. For example, many clinical trials are designed to investigate luminal inflammation exclusively. Whereas previous epidemiological^10,11,21^, genome-wide association studies^5,22–25^ and national consensus panels^26^ directed clinicians’ focus mostly to other obvious chronic inflammatory conditions such as musculoskeletal, dermatological, ophthalmological disorders or hepatobiliary disorders, our work uncovers - for the first time - that mental, behavioral and neurodevelopmental disorders, especially anxiety and depression actually are most frequently associated with IBD. The lack of attention to this problem^27,28^ is perhaps more surprising than the actual finding since a bi-directional brain-gut axis is a well-established concept^29^ and neuroinflammation^30^ and other molecular mechanisms^31^ for the disturbed blood–brain barrier have been recently unraveled.

Strengths of our work include the population-representative nature of this study with the inclusion of patients from academic and community healthcare centers, rural and urban settings, ethnic and cultural diversity, nearly two-decade observation period, and the ability to interrogate the data set beyond our analysis through the supplied interactive figures and software applications.

The results we present further validate, but also expand our understanding of extraintestinal manifestations and associated autoimmune disorders in IBD. We would like to highlight and discuss the following findings.

Our work emphasizes the importance of cerebrovascular and circulatory disorders. Major cardiovascular, cerebrovascular, and other thromboembolic events have recently mostly received attention in the context of severely hospitalized^32^ IBD patients for which preventive therapies have been recommended^33^ or as side effects of novel small molecules for the management of IBD, such as Janus kinase (JAK) inhibitors^6^. Since the JAK inhibitor exposure was only 0.5%, but their frequency was 20 times higher, this appears to be an independent phenomenon. There are alternative mechanistic explanations involving heat shock protein 47 and its’ regulation of thromboinflammation^34^. Our findings are supported by a population-based, sibling-controlled cohort study from Sweden between 1969 and 2019 that reported an increased risk of mostly ischemic strokes in IBD^35^. The increased risk of thromboembolic events independent of age-related processes and medications is further highlighted by reports of their occurrence in children^36^ and young adults^37^ and genetic susceptibilty^38^. A literature search across PubMed since its inception has reported an increased frequency of cardiovascular events in IBD^39,40^ and several molecular mechanisms have been proposed^41,42^.

The findings of our work are supported by a British Danish study currently not fully published but presented at a United European Gastroenterology Week (UEGW)^43^. While they looked at all comorbidities and we focused on EIM and AID, i.e. disorders that are mechanistically related to IBD, as stated in our methods section, we both arrive at comparable findings and independently conclude that IBD is a multisystemic disease, particularly manifesting with metabolic, immune, and neuropsychological disorders and that some conditions substantially precede diagnosis of CD or UC. We consider the independent, indirect validation of our results another major strength of our work.

Our work has limitations. We have used a very stringent selection algorithm, one that errs on the side of specificity and due to its design excludes early IBD. This is a compromise we accepted for this study to ensure we are really dealing with established, rather than suspected IBD. Our analyses rely on ICD codes. These codes and their WHO-assigned domains themselves have limitations in the sense that they do not always include our latest pathophysiology-driven understanding and classification of the respective diseases and sometimes also change their designation. We are also aware of potential coding errors, especially when it comes to the trailing first, second, or even third digits. Coding in Alberta’s electronic health record system *does not exclusively rely on clinicians*, whose initial choice of codes undergoes a variety of plausibility tests, independent validations, and corrections as necessary. None of these quality assurance measures, however, can completely rule out errors. Although tempting, associated codes, i.e., the coincidence of disorders, do not necessarily establish causality. This, however, is also true for genome, microbiome, or metabolome-wide association studies. Lastly, EIM and AID frequencies are likely also related to patient demographic factors, disease extent, disease activity,y and disease complications over time as well as treatment details. Not all these details are available in our data set, but future work in our group will attempt to investigate and dissect their impact.

Overall, our results support the hypothesis that IBD is a systemic inflammatory disorder with new and further reaching evidence from a large population-representative data set that requires a holistic view of the patient, including a multidisciplinary approach beyond digestive diseases^44^.

The power of interactive visualization in our work may help remind clinicians in their daily practice to actively look for EIM and AIDS, especially cardiovascular and mental disorders, that have not received the same attention as others in the past. We believe that our work can inspire and drive new research, using basic and clinical data complementing the common vision of precision health.

## Materials and methods

### The healthcare system in Alberta

Alberta is home to more than 4.85 million people. The population has diverse ethnic and cultural origins with 250 distinct groups including First Nations and immigrants from all continents according to the latest census of Statistics Canada^45^. All legal residents of Alberta are entitled to publicly funded and administered healthcare. Their care is documented in the Alberta Electronic Health Record Information System (EHRIS) dating back to 1997. EHRIS is jointly operated by Alberta’s Ministry of Health and Alberta Health Services^46^. Its main domains comprise access tools, repositories, registries, and infrastructure. All available data items are cataloged in the Alberta Health Data Asset Directory and the Alberta Health Services Data Asset Inventory Summary.

### The Alberta Inflammatory Bowel Disease Patient Registry

A Provincial IBD Patient Registry was developed and implemented by author D.C.B. in the Alberta EHRIS Connect Care, which is based on a highly customized version of Epic Hyperspace (Epic Inc., WI, USA). IBD patients as well as their EIM and frequently associated AID were identified with the Canadian versions of the WHO International Classification of Diseases (ICD) codes ICD-10CA (K50.X or K51.X) and ICD-9CA (555.X or 556.X) and their respective systematized nomenclature of medicine clinical terms (SNOMED CT).

The selection criteria for the analysis cohort were refined with a previously validated algorithm.

It was derived from a total of 150 IBD case definitions using 1399 IBD patients and 15,439 controls in the development phase. In the validation phase, 318,382 endoscopic procedures were searched and 5201 IBD patients were identified. After consideration of the sensitivity, specificity, and temporal stability of each validated case definition, a diagnosis of IBD was assigned to individuals who experienced at least two hospitalizations or had four physician claims, or two medical contacts in the Ambulatory Care Classification System database with an IBD diagnostic code within a 2-year period (specificity 99.8%; sensitivity 83.4%; positive predictive value 97.4%; negative predictive value 98.5%). An alternative case definition was developed for regions without access to the Ambulatory Care Classification System database. A novel scoring system was developed that detected Crohn's disease and ulcerative colitis patients with a specificity of >99% and a sensitivity of 99.1% and 86.3%, respectively^13^. Those not meeting either criteria were labeled inflammatory bowel disease undetermined (IBDU). Similar algorithms have been reliably used by various IBD researchers all over the world^47^.

### Extraintestinal manifestations (EIM) and associated autoimmune disorders (AID)

Historically, EIM and AID were thought to be limited to a very small number of inflammatory skin (i.e. psoriasis), joint (“arthopathy”,/“arthritis”), eye (i.e. uveitis), and liver diseases (i.e. PSC). However, our understanding of the systemic nature of Crohn’s disease and ulcerative colitis has substantially evolved due to the study of the genome, microbiome, and more recently metabolome^8^. This has impacted the most recent professional society treatment guidelines^9^. There they recognize the following conditions: musculoskeletal, ocular, oral, aural, nasal, skin, urogenital, hepato-pancreatico-biliary, neurological, cardiovascular, pulmonary, hematological, and endocrine. All of these conditions are grouped broadly into multifocal inflammation^8^ or extraintestinal manifestations (EIM) and associated immune disorders (AID). EIM has a clearly established molecular mechanistic basis while the mechanisms of AID have not been fully elucidated. Some of these conditions may also be disease complications and/or aggravated by their treatment. It is important to recognize that we have not tried to look at all comorbidities of IBD or make any claims about the incidence or prevalence of these conditions compared with the average population, which would be an epidemiological study and a very different research goal.

### Ethics approval

The study did not require informed consent and the protocol was approved by the Health Research Ethics Board of the University of Alberta Institutional Review Board (Pro00093304).

#### Statistical analyses

For descriptive statistics, medians, and interquartile ranges (IQR) were reported where applicable. Statistical significance was tested using the Chi-Square test for binary variables. All analyses are based on available data. Patient demographic variables (age, gender, disease type [CD, UC, IBD], medication exposure [main drug classes and individual compounds], and healthcare utilization [number of hospitalizations per year, number of ER visits per year] are reported.

### Louvain community detection network analysis

To identify and visualize communities of EIM and AID in relation to CD, UC, and IBD (CD + UC + IBDU) as defined above^13^, we deployed a previously described method to extract the community structure of large networks. It is a heuristic method based on modularity optimization, which was previously shown to outperform all other known community detection methods in terms of computation time, quality of the community detection, and accuracy validated in a network with 118 million nodes and more than one billion edges (links)^48,49^. This graph-theoretic method is a systems medicine approach to the clinical challenge of EIM and AID and if performed according to recent recommendations is robust to reliably reveal and visualize their associations with either CD or UC^50^.

The visualization consisting of nodes and edges is based on vis-network^51^. Vis-network uses HTML canvas for rendering. Therefore, the interactive figures should be viewed in the online appendix with a modern web browser. In this unbiased analysis, the *size* of an ICD code-derived *node* is proportional to √*n* × 0.3 + 2.5, where *n* is the number of unique patients assigned with that ICD. The thickness of each edge for a pair of ICDs = *m* × 0.01, where *m* is the number of patients having both of those ICDs, i.e. the strength of the disease relationship. The *colors of the nodes and edges* identify nodes in the detected community (cluster). The *node positions* are randomly set, governed by the force-atlas-2-based physics of the vis-network.

When one hovers over a *node*, it shows the tooltip with: the ICD code, ICD full name, and the absolute number and percentage of patients with that ICD code per disease, respectively. When one hovers over an *edge*, it shows the tooltip with: ICD codes of the nodes at the ends of the edge, and the number of patients having both codes, where —>, == and <— indicate the order of occurrence (timeline) of their first diagnoses. This allows one to determine for any ICD-coded condition whether it precedes or succeeds the condition it is linked to. One can drag and rearrange nodes to enable reading the edge tooltips. One can also set the minimum number of patient pairs for edges or the maximum length of the ICD code, to display a sub-network of interest.

#### Louvain community detection robustness evaluation

Overlap of ICD codes between corresponding communities identified by consecutive runs of the Louvain algorithm was scored using the Dice-Sørensen coefficient.

To evaluate *variability arising from the greedy nature of the Louvain algorithm* we performed consecutive runs of Louvain on all patient data but shuffled the order of ICD code co-occurrences input to each run. In each experiment, we scored the average overlap in ICD codes between corresponding communities detected by consecutive runs and averaged that value over all runs.

To evaluate *variability arising from patient sampling* we generated community detection results on two equally sized, mutually exclusive, random partitions of patients, we created an ICD co-occurrence graph and performed community detection for each partition, and calculated community overlap. When studying variability arising from differences in the patient samples we controlled algorithmic variability by inputting ICD co-occurrences into the Louvain algorithm in fixed alphabetical order. To assess whether the most frequently observed ICD codes form more consistent community associations than infrequent ones we calculated overlap scores using ICD codes that appeared in either the zeroth, first, second, or third quartiles of ICD code frequencies in the patient population.

We compared the results with a null distribution of overlap scores generated by randomly assigning ICD codes to communities and reported the mean and its standard error (SEM) over 1000 repeated trials.

## Supplementary information

Supplementary Information

## Data Availability

Our online supplementary information includes data tables, interactive html online figures, and our app allows for virtually unlimited opportunities to further explore the data set. The raw, patient-level source data that support the findings of this study were provided by Alberta Health Services and the Alberta Real World Evidence Consortium and are not publicly available. Researchers may file reasonable requests with these entities and data availability is governed by applicable law.

## Code availability

The underlying code for this study is not publicly available but may be made available to qualified researchers on reasonable request from the corresponding author.
