# Supplementary material for: Network analysis of extraintestinal manifestations and associated autoimmune disorders in Crohn’s disease and ulcerative colitis
Source: NPJ Digit Med. 2025 Apr 15;8:209. doi: 10.1038/s41746-025-01504-6 (PMC12000450; doi:10.1038/s41746-025-01504-6)
Supplement: Supplementary file 1 — Supplementary Information [file 41746_2025_1504_MOESM1_ESM.pdf]

Supplementary Tables

Supplementary Table 1: Mental. Behavioral and Neurodevelopmental disorders 2
Supplementary Table 2: Diseases of the musculoskeletal system and connective tissue 4
Supplementary Table 3: Diseases of the genitourinary system 50
Supplementary Table 4: Cerebrovascular diseases 53
Supplementary Table 5: Diseases of the circulatory system 68
Supplementary Table 6: Diseases of the respiratory system 73
Supplementary Table 7: Symptoms. signs. and abnormal clinical and laboratory findings. not elsewhere classified 77
Supplementary Table 8: Diseases of the digestive system 78
Supplementary Table 9: Diseases of the blood and blood-forming organs and certain disorders involving the immune mechanism 81
Supplementary Table 10: Diseases of the skin and subcutaneous tissue 87
Supplementary Table 11: Diseases of the nervous system 90
Supplementary Table 12: Endocrine. nutritional. and metabolic diseases 92
Supplementary Table 13: Diseases of the eye and adnexa 97
Supplementary Table 14: Diseases of the ear and mastoid process 102

Supplementary Table 1: Mental. Behavioral and Neurodevelopmental disorders

Organ System	Extraintestinal Manifestations and Associated Immune Disorders	ICD-10	Crohn's Disease		Ulcerative Colitis		IBD-Unclassified		IBD-Total (CD + UC + IBDU)		Comparison
			N = 15924		N = 11718		N = 2692		N = 30334		UC vs. CD
			n	%	n	%	n	%	n	%	p value ≤
**Mental. Behavioral and Neurodevelopmental disorders**											
	**Depressive episode**	**F32**	0	0.000	0	0.000	0	0.000	0	0.000	0.0000
	Major depressive disorder. single episode. mild	F32.0	9	0.057	8	0.068	0	0.000	17	0.056	0.8855
	Major depressive disorder. single episode. moderate	F32.1	35	0.220	29	0.247	4	0.149	68	0.224	0.7288
	Major depressive disorder. single episode. severe without psychotic features	F32.2	403	2.531	197	1.681	76	2.823	676	2.229	0.0000
	Major depressive disorder. single episode. severe with psychotic features	F32.3	46	0.289	28	0.239	10	0.371	84	0.277	0.4990
	Major depressive disorder. single episode. in partial remission	F32.4	0	0.000	0	0.000	0	0.000	0	0.000	0.0000
	Major depressive disorder. single episode. in full remission	F32.5	0	0.000	0	0.000	0	0.000	0	0.000	0.0000
	Other depressive episodes	F32.8	59	0.371	25	0.213	10	0.371	94	0.310	0.0254
	Premenstrual dysphoric disorder	F32.81	0	0.000	0	0.000	0	0.000	0	0.000	0.0000
	Other specified depressive episodes	F32.89	0	0.000	0	0.000	0	0.000	0	0.000	0.0000
	Major depressive disorder. single episode. unspecified	F32.9	1568	9.847	877	7.484	296	10.996	2741	9.036	0.0000
	Depression. unspecified	F32.A	0	0.000	0	0.000	0	0.000	0	0.000	0.0000
	**Major depressive disorder. recurrent**	**F33**	0	0.000	0	0.000	0	0.000	0	0.000	0.0000
	Major depressive disorder. recurrent. mild	F33.0	15	0.094	7	0.060	2	0.074	24	0.079	0.4306
	Major depressive disorder. recurrent. moderate	F33.1	102	0.641	78	0.666	21	0.780	201	0.663	0.8566
	Major depressive disorder. recurrent severe without psychotic features	F33.2	113	0.710	48	0.410	14	0.520	175	0.577	0.0016
	Major depressive disorder. recurrent. severe with psychotic symptoms	F33.3	13	0.082	7	0.060	0	0.000	20	0.066	0.6579
	Major depressive disorder. recurrent. in remission	F33.4	34	0.214	15	0.128	7	0.260	56	0.185	0.1272
	Major depressive disorder. recurrent. in remission. unspecified	F33.40	0	0.000	0	0.000	0	0.000	0	0.000	0.0000

	Major depressive disorder. recurrent. in partial remission	F33.41	0	0.000	0	0.000	0	0.000	0	0.000	0.0000
	Major depressive disorder. recurrent. in full remission	F33.42	0	0.000	0	0.000	0	0.000	0	0.000	0.0000
	Other recurrent depressive disorders	F33.8	35	0.220	28	0.239	3	0.111	66	0.218	0.8396
	Major depressive disorder. recurrent. unspecified	F33.9	296	1.859	152	1.297	53	1.969	501	1.652	0.0003
	**Persistent mood [affective] disorders**	**F34**	0	0.000	0	0.000	0	0.000	0	0.000	0.0000
	Cyclothymic disorder	F34.0	3	0.019	1	0.009	2	0.074	6	0.020	0.8430
	Dysthymic disorder	F34.1	204	1.281	115	0.981	35	1.300	354	1.167	0.0245
	Other persistent mood [affective] disorders	F34.8	1	0.006	2	0.017	0	0.000	3	0.010	0.7897
	Disruptive mood dysregulation disorder	F34.81	0	0.000	0	0.000	0	0.000	0	0.000	0.0000
	Other specified persistent mood disorders	F34.89	0	0.000	0	0.000	0	0.000	0	0.000	0.0000
	Persistent mood [affective] disorder. unspecified	F34.9	3	0.019	0	0.000	0	0.000	3	0.010	0.3672
	Unspecified mood [affective] disorder	F39	144	0.904	83	0.708	28	1.040	255	0.841	0.0860
	**Other anxiety disorders**	**F41**	0	0.000	0	0.000	0	0.000	0	0.000	0.0000
	Panic disorder [episodic paroxysmal anxiety]	F41.0	289	1.815	168	1.434	50	1.857	507	1.671	0.0160
	Generalized anxiety disorder	F41.1	451	2.832	280	2.389	80	2.972	811	2.674	0.0258
	Other mixed anxiety disorders	F41.3	351	2.204	176	1.502	75	2.786	602	1.985	0.0000
	Other specified anxiety disorders	F41.8	65	0.408	39	0.333	12	0.446	116	0.382	0.3617
	Anxiety disorder. unspecified	F41.9	1602	10.060	987	8.423	303	11.256	2892	9.534	0.0000
**Total**			3095	19.436	1869	15.950	537	19.948	5501	18.135	0.0000

Supplementary Table 2: Diseases of the musculoskeletal system and connective tissue

Organ System	Extraintestinal Manifestations and Associated Immune Disorders	ICD-10	Crohn's Disease		Ulcerative Colitis		IBD-Unclassified		IBD-Total (CD + UC + IBDU)		Comparison
			N = 15924		N = 11718		N = 2692		N = 30334		UC vs. CD
			n		n		n		n		p value
				%		%		%		%	≤
**Diseases of the musculoskeletal system and connective tissue**											
	**Postinfective and reactive arthropathies**	**M02**	0	0.000	0	0.000	0	0.000	0	0.000	0.0000
	Arthropathy following intestinal bypass	M02.0	0	0.000	0	0.000	0	0.000	0	0.000	0.0000
	Arthropathy following intestinal bypass. unspecified site	M02.00	0	0.000	0	0.000	1	0.037	1	0.003	0.0000
	Arthropathy following intestinal bypass. shoulder	M02.01	0	0.000	0	0.000	0	0.000	0	0.000	0.0000
	Arthropathy following intestinal bypass. right shoulder	M02.011	0	0.000	0	0.000	0	0.000	0	0.000	0.0000
	Arthropathy following intestinal bypass. left shoulder	M02.012	0	0.000	0	0.000	0	0.000	0	0.000	0.0000
	Arthropathy following intestinal bypass. unspecified shoulder	M02.019	0	0.000	0	0.000	0	0.000	0	0.000	0.0000
	Arthropathy following intestinal bypass. elbow	M02.02	0	0.000	0	0.000	0	0.000	0	0.000	0.0000
	Arthropathy following intestinal bypass. right elbow	M02.021	0	0.000	0	0.000	0	0.000	0	0.000	0.0000
	Arthropathy following intestinal bypass. left elbow	M02.022	0	0.000	0	0.000	0	0.000	0	0.000	0.0000
	Arthropathy following intestinal bypass. unspecified elbow	M02.029	0	0.000	0	0.000	0	0.000	0	0.000	0.0000
	Arthropathy following intestinal bypass. wrist	M02.03	0	0.000	0	0.000	0	0.000	0	0.000	0.0000
	Arthropathy following intestinal bypass. right wrist	M02.031	0	0.000	0	0.000	0	0.000	0	0.000	0.0000
	Arthropathy following intestinal bypass. left wrist	M02.032	0	0.000	0	0.000	0	0.000	0	0.000	0.0000
	Arthropathy following intestinal bypass. unspecified wrist	M02.039	0	0.000	0	0.000	0	0.000	0	0.000	0.0000
	Arthropathy following intestinal bypass. hand	M02.04	0	0.000	0	0.000	0	0.000	0	0.000	0.0000
	Arthropathy following intestinal bypass. right hand	M02.041	0	0.000	0	0.000	0	0.000	0	0.000	0.0000
	Arthropathy following intestinal bypass. left hand	M02.042	0	0.000	0	0.000	0	0.000	0	0.000	0.0000
	Arthropathy following intestinal bypass. unspecified hand	M02.049	0	0.000	0	0.000	0	0.000	0	0.000	0.0000
	Arthropathy following intestinal bypass. hip	M02.05	0	0.000	0	0.000	0	0.000	0	0.000	0.0000

	Arthropathy following intestinal bypass. right hip	M02.051	0	0.000	0	0.000	0	0.000	0	0.000	0.0000
	Arthropathy following intestinal bypass. left hip	M02.052	0	0.000	0	0.000	0	0.000	0	0.000	0.0000
	Arthropathy following intestinal bypass. unspecified hip	M02.059	0	0.000	0	0.000	0	0.000	0	0.000	0.0000
	Arthropathy following intestinal bypass. knee	M02.06	0	0.000	0	0.000	0	0.000	0	0.000	0.0000
	Arthropathy following intestinal bypass. right knee	M02.061	0	0.000	0	0.000	0	0.000	0	0.000	0.0000
	Arthropathy following intestinal bypass. left knee	M02.062	0	0.000	0	0.000	0	0.000	0	0.000	0.0000
	Arthropathy following intestinal bypass. unspecified knee	M02.069	0	0.000	0	0.000	0	0.000	0	0.000	0.0000
	Arthropathy following intestinal bypass. ankle and foot	M02.07	0	0.000	0	0.000	0	0.000	0	0.000	0.0000
	Arthropathy following intestinal bypass. right ankle and foot	M02.071	0	0.000	0	0.000	0	0.000	0	0.000	0.0000
	Arthropathy following intestinal bypass. left ankle and foot	M02.072	0	0.000	0	0.000	0	0.000	0	0.000	0.0000
	Arthropathy following intestinal bypass. unspecified ankle and foot	M02.079	0	0.000	0	0.000	0	0.000	0	0.000	0.0000
	Arthropathy following intestinal bypass. vertebrae	M02.08	0	0.000	0	0.000	0	0.000	0	0.000	0.0000
	Arthropathy following intestinal bypass. multiple sites	M02.09	0	0.000	0	0.000	0	0.000	0	0.000	0.0000
	Postdysenteric arthropathy	M02.1	0	0.000	0	0.000	0	0.000	0	0.000	0.0000
	Postdysenteric arthropathy. unspecified site	M02.10	0	0.000	0	0.000	0	0.000	0	0.000	0.0000
	Postdysenteric arthropathy. shoulder	M02.11	0	0.000	0	0.000	0	0.000	0	0.000	0.0000
	Postdysenteric arthropathy. right shoulder	M02.111	0	0.000	0	0.000	0	0.000	0	0.000	0.0000
	Postdysenteric arthropathy. left shoulder	M02.112	0	0.000	0	0.000	0	0.000	0	0.000	0.0000
	Postdysenteric arthropathy. unspecified shoulder	M02.119	0	0.000	0	0.000	0	0.000	0	0.000	0.0000
	Postdysenteric arthropathy. elbow	M02.12	0	0.000	0	0.000	0	0.000	0	0.000	0.0000
	Postdysenteric arthropathy. right elbow	M02.121	0	0.000	0	0.000	0	0.000	0	0.000	0.0000
	Postdysenteric arthropathy. left elbow	M02.122	0	0.000	0	0.000	0	0.000	0	0.000	0.0000
	Postdysenteric arthropathy. unspecified elbow	M02.129	0	0.000	0	0.000	0	0.000	0	0.000	0.0000
	Postdysenteric arthropathy. wrist	M02.13	0	0.000	0	0.000	0	0.000	0	0.000	0.0000
	Postdysenteric arthropathy. right wrist	M02.131	0	0.000	0	0.000	0	0.000	0	0.000	0.0000
	Postdysenteric arthropathy. left wrist	M02.132	0	0.000	0	0.000	0	0.000	0	0.000	0.0000
	Postdysenteric arthropathy. unspecified wrist	M02.139	0	0.000	0	0.000	0	0.000	0	0.000	0.0000
	Postdysenteric arthropathy. hand	M02.14	0	0.000	0	0.000	0	0.000	0	0.000	0.0000
	Postdysenteric arthropathy. right hand	M02.141	0	0.000	0	0.000	0	0.000	0	0.000	0.0000

	Postdysenteric arthropathy. left hand	M02.142	0	0.000	0	0.000	0	0.000	0	0.000	0.0000
	Postdysenteric arthropathy. unspecified hand	M02.149	0	0.000	0	0.000	0	0.000	0	0.000	0.0000
	Postdysenteric arthropathy. hip	M02.15	0	0.000	0	0.000	0	0.000	0	0.000	0.0000
	Postdysenteric arthropathy. right hip	M02.151	0	0.000	0	0.000	0	0.000	0	0.000	0.0000
	Postdysenteric arthropathy. left hip	M02.152	0	0.000	0	0.000	0	0.000	0	0.000	0.0000
	Postdysenteric arthropathy. unspecified hip	M02.159	0	0.000	0	0.000	0	0.000	0	0.000	0.0000
	Postdysenteric arthropathy. knee	M02.16	0	0.000	0	0.000	0	0.000	0	0.000	0.0000
	Postdysenteric arthropathy. right knee	M02.161	0	0.000	0	0.000	0	0.000	0	0.000	0.0000
	Postdysenteric arthropathy. left knee	M02.162	0	0.000	0	0.000	0	0.000	0	0.000	0.0000
	Postdysenteric arthropathy. unspecified knee	M02.169	0	0.000	0	0.000	0	0.000	0	0.000	0.0000
	Postdysenteric arthropathy. ankle and foot	M02.17	0	0.000	0	0.000	0	0.000	0	0.000	0.0000
	Postdysenteric arthropathy. right ankle and foot	M02.171	0	0.000	0	0.000	0	0.000	0	0.000	0.0000
	Postdysenteric arthropathy. left ankle and foot	M02.172	0	0.000	0	0.000	0	0.000	0	0.000	0.0000
	Postdysenteric arthropathy. unspecified ankle and foot	M02.179	0	0.000	0	0.000	0	0.000	0	0.000	0.0000
	Postdysenteric arthropathy. vertebrae	M02.18	0	0.000	0	0.000	0	0.000	0	0.000	0.0000
	Postdysenteric arthropathy. multiple sites	M02.19	0	0.000	0	0.000	0	0.000	0	0.000	0.0000
	Postimmunization arthropathy	M02.2	0	0.000	0	0.000	0	0.000	0	0.000	0.0000
	Postimmunization arthropathy. unspecified site	M02.20	0	0.000	0	0.000	0	0.000	0	0.000	0.0000
	Postimmunization arthropathy. shoulder	M02.21	0	0.000	0	0.000	0	0.000	0	0.000	0.0000
	Postimmunization arthropathy. right shoulder	M02.211	0	0.000	0	0.000	0	0.000	0	0.000	0.0000
	Postimmunization arthropathy. left shoulder	M02.212	0	0.000	0	0.000	0	0.000	0	0.000	0.0000
	Postimmunization arthropathy. unspecified shoulder	M02.219	0	0.000	0	0.000	0	0.000	0	0.000	0.0000
	Postimmunization arthropathy. elbow	M02.22	0	0.000	0	0.000	0	0.000	0	0.000	0.0000
	Postimmunization arthropathy. right elbow	M02.221	0	0.000	0	0.000	0	0.000	0	0.000	0.0000
	Postimmunization arthropathy. left elbow	M02.222	0	0.000	0	0.000	0	0.000	0	0.000	0.0000
	Postimmunization arthropathy. unspecified elbow	M02.229	0	0.000	0	0.000	0	0.000	0	0.000	0.0000
	Postimmunization arthropathy. wrist	M02.23	0	0.000	0	0.000	0	0.000	0	0.000	0.0000
	Postimmunization arthropathy. right wrist	M02.231	0	0.000	0	0.000	0	0.000	0	0.000	0.0000
	Postimmunization arthropathy. left wrist	M02.232	0	0.000	0	0.000	0	0.000	0	0.000	0.0000

	Postimmunization arthropathy. unspecified wrist	M02.239	0	0.000	0	0.000	0	0.000	0	0.000	0.0000
	Postimmunization arthropathy. hand	M02.24	0	0.000	0	0.000	0	0.000	0	0.000	0.0000
	Postimmunization arthropathy. right hand	M02.241	0	0.000	0	0.000	0	0.000	0	0.000	0.0000
	Postimmunization arthropathy. left hand	M02.242	0	0.000	0	0.000	0	0.000	0	0.000	0.0000
	Postimmunization arthropathy. unspecified hand	M02.249	0	0.000	0	0.000	0	0.000	0	0.000	0.0000
	Postimmunization arthropathy. hip	M02.25	0	0.000	0	0.000	0	0.000	0	0.000	0.0000
	Postimmunization arthropathy. right hip	M02.251	0	0.000	0	0.000	0	0.000	0	0.000	0.0000
	Postimmunization arthropathy. left hip	M02.252	0	0.000	0	0.000	0	0.000	0	0.000	0.0000
	Postimmunization arthropathy. unspecified hip	M02.259	0	0.000	0	0.000	0	0.000	0	0.000	0.0000
	Postimmunization arthropathy. knee	M02.26	0	0.000	0	0.000	0	0.000	0	0.000	0.0000
	Postimmunization arthropathy. right knee	M02.261	0	0.000	0	0.000	0	0.000	0	0.000	0.0000
	Postimmunization arthropathy. left knee	M02.262	0	0.000	0	0.000	0	0.000	0	0.000	0.0000
	Postimmunization arthropathy. unspecified knee	M02.269	0	0.000	0	0.000	0	0.000	0	0.000	0.0000
	Postimmunization arthropathy. ankle and foot	M02.27	0	0.000	0	0.000	0	0.000	0	0.000	0.0000
	Postimmunization arthropathy. right ankle and foot	M02.271	0	0.000	0	0.000	0	0.000	0	0.000	0.0000
	Postimmunization arthropathy. left ankle and foot	M02.272	0	0.000	0	0.000	0	0.000	0	0.000	0.0000
	Postimmunization arthropathy. unspecified ankle and foot	M02.279	0	0.000	0	0.000	0	0.000	0	0.000	0.0000
	Postimmunization arthropathy. vertebrae	M02.28	0	0.000	0	0.000	0	0.000	0	0.000	0.0000
	Postimmunization arthropathy. multiple sites	M02.29	0	0.000	0	0.000	0	0.000	0	0.000	0.0000
	Reiter's disease	M02.3	0	0.000	0	0.000	0	0.000	0	0.000	0.0000
	Reiter's disease. unspecified site	M02.30	0	0.000	0	0.000	0	0.000	0	0.000	0.0000
	Reiter's disease. shoulder	M02.31	0	0.000	0	0.000	0	0.000	0	0.000	0.0000
	Reiter's disease. right shoulder	M02.311	0	0.000	0	0.000	0	0.000	0	0.000	0.0000
	Reiter's disease. left shoulder	M02.312	0	0.000	0	0.000	0	0.000	0	0.000	0.0000
	Reiter's disease. unspecified shoulder	M02.319	0	0.000	0	0.000	0	0.000	0	0.000	0.0000
	Reiter's disease. elbow	M02.32	0	0.000	0	0.000	0	0.000	0	0.000	0.0000
	Reiter's disease. right elbow	M02.321	0	0.000	0	0.000	0	0.000	0	0.000	0.0000
	Reiter's disease. left elbow	M02.322	0	0.000	0	0.000	0	0.000	0	0.000	0.0000
	Reiter's disease. unspecified elbow	M02.329	0	0.000	0	0.000	0	0.000	0	0.000	0.0000

	Reiter's disease. wrist	M02.33	0	0.000	0	0.000	0	0.000	0	0.000	0.0000
	Reiter's disease. right wrist	M02.331	0	0.000	0	0.000	0	0.000	0	0.000	0.0000
	Reiter's disease. left wrist	M02.332	0	0.000	0	0.000	0	0.000	0	0.000	0.0000
	Reiter's disease. unspecified wrist	M02.339	0	0.000	0	0.000	0	0.000	0	0.000	0.0000
	Reiter's disease. hand	M02.34	0	0.000	0	0.000	0	0.000	0	0.000	0.0000
	Reiter's disease. right hand	M02.341	0	0.000	0	0.000	0	0.000	0	0.000	0.0000
	Reiter's disease. left hand	M02.342	0	0.000	0	0.000	0	0.000	0	0.000	0.0000
	Reiter's disease. unspecified hand	M02.349	0	0.000	0	0.000	0	0.000	0	0.000	0.0000
	Reiter's disease. hip	M02.35	0	0.000	0	0.000	0	0.000	0	0.000	0.0000
	Reiter's disease. right hip	M02.351	0	0.000	0	0.000	0	0.000	0	0.000	0.0000
	Reiter's disease. left hip	M02.352	0	0.000	0	0.000	0	0.000	0	0.000	0.0000
	Reiter's disease. unspecified hip	M02.359	0	0.000	0	0.000	0	0.000	0	0.000	0.0000
	Reiter's disease. knee	M02.36	1	0.006	0	0.000	0	0.000	1	0.003	0.8776
	Reiter's disease. right knee	M02.361	0	0.000	0	0.000	0	0.000	0	0.000	0.0000
	Reiter's disease. left knee	M02.362	0	0.000	0	0.000	0	0.000	0	0.000	0.0000
	Reiter's disease. unspecified knee	M02.369	0	0.000	0	0.000	0	0.000	0	0.000	0.0000
	Reiter's disease. ankle and foot	M02.37	2	0.013	0	0.000	0	0.000	2	0.007	0.6187
	Reiter's disease. right ankle and foot	M02.371	0	0.000	0	0.000	0	0.000	0	0.000	0.0000
	Reiter's disease. left ankle and foot	M02.372	0	0.000	0	0.000	0	0.000	0	0.000	0.0000
	Reiter's disease. unspecified ankle and foot	M02.379	0	0.000	0	0.000	0	0.000	0	0.000	0.0000
	Reiter's disease. vertebrae	M02.38	0	0.000	1	0.009	0	0.000	1	0.003	0.8776
	Reiter's disease. multiple sites	M02.39	9	0.057	4	0.034	2	0.074	15	0.049	0.5704
	Other reactive arthropathies	M02.8	0	0.000	0	0.000	0	0.000	0	0.000	0.0000
	Other reactive arthropathies. unspecified site	M02.80	2	0.013	1	0.009	0	0.000	3	0.010	0.7897
	Other reactive arthropathies. shoulder	M02.81	0	0.000	0	0.000	0	0.000	0	0.000	0.0000
	Other reactive arthropathies. right shoulder	M02.811	0	0.000	0	0.000	0	0.000	0	0.000	0.0000
	Other reactive arthropathies. left shoulder	M02.812	0	0.000	0	0.000	0	0.000	0	0.000	0.0000
	Other reactive arthropathies. unspecified shoulder	M02.819	0	0.000	0	0.000	0	0.000	0	0.000	0.0000
	Other reactive arthropathies. elbow	M02.82	0	0.000	0	0.000	0	0.000	0	0.000	0.0000

	Other reactive arthropathies. right elbow	M02.821	0	0.000	0	0.000	0	0.000	0	0.000	0.0000
	Other reactive arthropathies. left elbow	M02.822	0	0.000	0	0.000	0	0.000	0	0.000	0.0000
	Other reactive arthropathies. unspecified elbow	M02.829	0	0.000	0	0.000	0	0.000	0	0.000	0.0000
	Other reactive arthropathies. wrist	M02.83	0	0.000	2	0.017	0	0.000	2	0.007	0.3507
	Other reactive arthropathies. right wrist	M02.831	0	0.000	0	0.000	0	0.000	0	0.000	0.0000
	Other reactive arthropathies. left wrist	M02.832	0	0.000	0	0.000	0	0.000	0	0.000	0.0000
	Other reactive arthropathies. unspecified wrist	M02.839	0	0.000	0	0.000	0	0.000	0	0.000	0.0000
	Other reactive arthropathies. hand	M02.84	0	0.000	0	0.000	0	0.000	0	0.000	0.0000
	Other reactive arthropathies. right hand	M02.841	0	0.000	0	0.000	0	0.000	0	0.000	0.0000
	Other reactive arthropathies. left hand	M02.842	0	0.000	0	0.000	0	0.000	0	0.000	0.0000
	Other reactive arthropathies. unspecified hand	M02.849	0	0.000	0	0.000	0	0.000	0	0.000	0.0000
	Other reactive arthropathies. hip	M02.85	0	0.000	0	0.000	0	0.000	0	0.000	0.0000
	Other reactive arthropathies. right hip	M02.851	0	0.000	0	0.000	0	0.000	0	0.000	0.0000
	Other reactive arthropathies. left hip	M02.852	0	0.000	0	0.000	0	0.000	0	0.000	0.0000
	Other reactive arthropathies. unspecified hip	M02.859	0	0.000	0	0.000	0	0.000	0	0.000	0.0000
	Other reactive arthropathies. knee	M02.86	0	0.000	0	0.000	0	0.000	0	0.000	0.0000
	Other reactive arthropathies. right knee	M02.861	0	0.000	0	0.000	0	0.000	0	0.000	0.0000
	Other reactive arthropathies. left knee	M02.862	0	0.000	0	0.000	0	0.000	0	0.000	0.0000
	Other reactive arthropathies. unspecified knee	M02.869	0	0.000	0	0.000	0	0.000	0	0.000	0.0000
	Other reactive arthropathies. ankle and foot	M02.87	1	0.006	0	0.000	0	0.000	1	0.003	0.8776
	Other reactive arthropathies. right ankle and foot	M02.871	0	0.000	0	0.000	0	0.000	0	0.000	0.0000
	Other reactive arthropathies. left ankle and foot	M02.872	0	0.000	0	0.000	0	0.000	0	0.000	0.0000
	Other reactive arthropathies. unspecified ankle and foot	M02.879	0	0.000	0	0.000	0	0.000	0	0.000	0.0000
	Other reactive arthropathies. vertebrae	M02.88	1	0.006	0	0.000	0	0.000	1	0.003	0.8776
	Other reactive arthropathies. multiple sites	M02.89	0	0.000	0	0.000	0	0.000	0	0.000	0.0000
	Reactive arthropathy. unspecified	M02.9	0	0.000	0	0.000	0	0.000	0	0.000	0.0000
	**Autoinflammatory syndromes**	**M04**	0	0.000	0	0.000	0	0.000	0	0.000	0.0000
	Periodic fever syndromes	M04.1	0	0.000	0	0.000	0	0.000	0	0.000	0.0000
	Cryopyrin-associated periodic syndromes	M04.2	0	0.000	0	0.000	0	0.000	0	0.000	0.0000

	Other autoinflammatory syndromes	M04.8	0	0.000	0	0.000	0	0.000	0	0.000	0.0000
	Autoinflammatory syndrome. unspecified	M04.9	0	0.000	0	0.000	0	0.000	0	0.000	0.0000
	**Rheumatoid arthritis with rheumatoid factor**	**M05**	0	0.000	0	0.000	0	0.000	0	0.000	0.0000
	Felty's syndrome	M05.0	1	0.006	0	0.000	0	0.000	1	0.003	0.8776
	Felty's syndrome. unspecified site	M05.00	0	0.000	0	0.000	0	0.000	0	0.000	0.0000
	Felty's syndrome. shoulder	M05.01	0	0.000	0	0.000	0	0.000	0	0.000	0.0000
	Felty's syndrome. right shoulder	M05.011	0	0.000	0	0.000	0	0.000	0	0.000	0.0000
	Felty's syndrome. left shoulder	M05.012	0	0.000	0	0.000	0	0.000	0	0.000	0.0000
	Felty's syndrome. unspecified shoulder	M05.019	0	0.000	0	0.000	0	0.000	0	0.000	0.0000
	Felty's syndrome. elbow	M05.02	0	0.000	0	0.000	0	0.000	0	0.000	0.0000
	Felty's syndrome. right elbow	M05.021	0	0.000	0	0.000	0	0.000	0	0.000	0.0000
	Felty's syndrome. left elbow	M05.022	0	0.000	0	0.000	0	0.000	0	0.000	0.0000
	Felty's syndrome. unspecified elbow	M05.029	0	0.000	0	0.000	0	0.000	0	0.000	0.0000
	Felty's syndrome. wrist	M05.03	0	0.000	0	0.000	0	0.000	0	0.000	0.0000
	Felty's syndrome. right wrist	M05.031	0	0.000	0	0.000	0	0.000	0	0.000	0.0000
	Felty's syndrome. left wrist	M05.032	0	0.000	0	0.000	0	0.000	0	0.000	0.0000
	Felty's syndrome. unspecified wrist	M05.039	0	0.000	0	0.000	0	0.000	0	0.000	0.0000
	Felty's syndrome. hand	M05.04	0	0.000	0	0.000	0	0.000	0	0.000	0.0000
	Felty's syndrome. right hand	M05.041	0	0.000	0	0.000	0	0.000	0	0.000	0.0000
	Felty's syndrome. left hand	M05.042	0	0.000	0	0.000	0	0.000	0	0.000	0.0000
	Felty's syndrome. unspecified hand	M05.049	0	0.000	0	0.000	0	0.000	0	0.000	0.0000
	Felty's syndrome. hip	M05.05	0	0.000	0	0.000	0	0.000	0	0.000	0.0000
	Felty's syndrome. right hip	M05.051	0	0.000	0	0.000	0	0.000	0	0.000	0.0000
	Felty's syndrome. left hip	M05.052	0	0.000	0	0.000	0	0.000	0	0.000	0.0000
	Felty's syndrome. unspecified hip	M05.059	0	0.000	0	0.000	0	0.000	0	0.000	0.0000
	Felty's syndrome. knee	M05.06	0	0.000	0	0.000	0	0.000	0	0.000	0.0000
	Felty's syndrome. right knee	M05.061	0	0.000	0	0.000	0	0.000	0	0.000	0.0000
	Felty's syndrome. left knee	M05.062	0	0.000	0	0.000	0	0.000	0	0.000	0.0000
	Felty's syndrome. unspecified knee	M05.069	0	0.000	0	0.000	0	0.000	0	0.000	0.0000

	Felty's syndrome. ankle and foot	M05.07	0	0.000	0	0.000	0	0.000	0	0.000	0.0000
	Felty's syndrome. right ankle and foot	M05.071	0	0.000	0	0.000	0	0.000	0	0.000	0.0000
	Felty's syndrome. left ankle and foot	M05.072	0	0.000	0	0.000	0	0.000	0	0.000	0.0000
	Felty's syndrome. unspecified ankle and foot	M05.079	0	0.000	0	0.000	0	0.000	0	0.000	0.0000
	Felty's syndrome. multiple sites	M05.09	0	0.000	0	0.000	0	0.000	0	0.000	0.0000
	Rheumatoid lung disease with rheumatoid arthritis	M05.1	0	0.000	1	0.009	1	0.037	2	0.007	0.8776
	Rheumatoid lung disease with rheumatoid arthritis of unspecified site	M05.10	0	0.000	0	0.000	0	0.000	0	0.000	0.0000
	Rheumatoid lung disease with rheumatoid arthritis of shoulder	M05.11	0	0.000	0	0.000	0	0.000	0	0.000	0.0000
	Rheumatoid lung disease with rheumatoid arthritis of right shoulder	M05.111	0	0.000	0	0.000	0	0.000	0	0.000	0.0000
	Rheumatoid lung disease with rheumatoid arthritis of left shoulder	M05.112	0	0.000	0	0.000	0	0.000	0	0.000	0.0000
	Rheumatoid lung disease with rheumatoid arthritis of unspecified shoulder	M05.119	0	0.000	0	0.000	0	0.000	0	0.000	0.0000
	Rheumatoid lung disease with rheumatoid arthritis of elbow	M05.12	0	0.000	0	0.000	0	0.000	0	0.000	0.0000
	Rheumatoid lung disease with rheumatoid arthritis of right elbow	M05.121	0	0.000	0	0.000	0	0.000	0	0.000	0.0000
	Rheumatoid lung disease with rheumatoid arthritis of left elbow	M05.122	0	0.000	0	0.000	0	0.000	0	0.000	0.0000
	Rheumatoid lung disease with rheumatoid arthritis of unspecified elbow	M05.129	0	0.000	0	0.000	0	0.000	0	0.000	0.0000
	Rheumatoid lung disease with rheumatoid arthritis of wrist	M05.13	0	0.000	0	0.000	0	0.000	0	0.000	0.0000
	Rheumatoid lung disease with rheumatoid arthritis of right wrist	M05.131	0	0.000	0	0.000	0	0.000	0	0.000	0.0000
	Rheumatoid lung disease with rheumatoid arthritis of left wrist	M05.132	0	0.000	0	0.000	0	0.000	0	0.000	0.0000
	Rheumatoid lung disease with rheumatoid arthritis of unspecified wrist	M05.139	0	0.000	0	0.000	0	0.000	0	0.000	0.0000
	Rheumatoid lung disease with rheumatoid arthritis of hand	M05.14	0	0.000	0	0.000	0	0.000	0	0.000	0.0000
	Rheumatoid lung disease with rheumatoid arthritis of right hand	M05.141	0	0.000	0	0.000	0	0.000	0	0.000	0.0000
	Rheumatoid lung disease with rheumatoid arthritis of left hand	M05.142	0	0.000	0	0.000	0	0.000	0	0.000	0.0000
	Rheumatoid lung disease with rheumatoid arthritis of unspecified hand	M05.149	0	0.000	0	0.000	0	0.000	0	0.000	0.0000
	Rheumatoid lung disease with rheumatoid arthritis of hip	M05.15	0	0.000	0	0.000	0	0.000	0	0.000	0.0000
	Rheumatoid lung disease with rheumatoid arthritis of right hip	M05.151	0	0.000	0	0.000	0	0.000	0	0.000	0.0000
	Rheumatoid lung disease with rheumatoid arthritis of left hip	M05.152	0	0.000	0	0.000	0	0.000	0	0.000	0.0000

	Rheumatoid lung disease with rheumatoid arthritis of unspecified hip	M05.159	0	0.000	0	0.000	0	0.000	0	0.000	0.0000
	Rheumatoid lung disease with rheumatoid arthritis of knee	M05.16	0	0.000	0	0.000	0	0.000	0	0.000	0.0000
	Rheumatoid lung disease with rheumatoid arthritis of right knee	M05.161	0	0.000	0	0.000	0	0.000	0	0.000	0.0000
	Rheumatoid lung disease with rheumatoid arthritis of left knee	M05.162	0	0.000	0	0.000	0	0.000	0	0.000	0.0000
	Rheumatoid lung disease with rheumatoid arthritis of unspecified knee	M05.169	0	0.000	0	0.000	0	0.000	0	0.000	0.0000
	Rheumatoid lung disease with rheumatoid arthritis of ankle and foot	M05.17	0	0.000	0	0.000	0	0.000	0	0.000	0.0000
	Rheumatoid lung disease with rheumatoid arthritis of right ankle and foot	M05.171	0	0.000	0	0.000	0	0.000	0	0.000	0.0000
	Rheumatoid lung disease with rheumatoid arthritis of left ankle and foot	M05.172	0	0.000	0	0.000	0	0.000	0	0.000	0.0000
	Rheumatoid lung disease with rheumatoid arthritis of unspecified ankle and foot	M05.179	0	0.000	0	0.000	0	0.000	0	0.000	0.0000
	Rheumatoid lung disease with rheumatoid arthritis of multiple sites	M05.19	0	0.000	0	0.000	0	0.000	0	0.000	0.0000
	Rheumatoid vasculitis with rheumatoid arthritis	M05.2	0	0.000	3	0.026	1	0.037	4	0.013	0.1513
	Rheumatoid vasculitis with rheumatoid arthritis of unspecified site	M05.20	0	0.000	0	0.000	0	0.000	0	0.000	0.0000
	Rheumatoid vasculitis with rheumatoid arthritis of shoulder	M05.21	0	0.000	0	0.000	0	0.000	0	0.000	0.0000
	Rheumatoid vasculitis with rheumatoid arthritis of right shoulder	M05.211	0	0.000	0	0.000	0	0.000	0	0.000	0.0000
	Rheumatoid vasculitis with rheumatoid arthritis of left shoulder	M05.212	0	0.000	0	0.000	0	0.000	0	0.000	0.0000
	Rheumatoid vasculitis with rheumatoid arthritis of unspecified shoulder	M05.219	0	0.000	0	0.000	0	0.000	0	0.000	0.0000
	Rheumatoid vasculitis with rheumatoid arthritis of elbow	M05.22	0	0.000	0	0.000	0	0.000	0	0.000	0.0000
	Rheumatoid vasculitis with rheumatoid arthritis of right elbow	M05.221	0	0.000	0	0.000	0	0.000	0	0.000	0.0000
	Rheumatoid vasculitis with rheumatoid arthritis of left elbow	M05.222	0	0.000	0	0.000	0	0.000	0	0.000	0.0000
	Rheumatoid vasculitis with rheumatoid arthritis of unspecified elbow	M05.229	0	0.000	0	0.000	0	0.000	0	0.000	0.0000
	Rheumatoid vasculitis with rheumatoid arthritis of wrist	M05.23	0	0.000	0	0.000	0	0.000	0	0.000	0.0000
	Rheumatoid vasculitis with rheumatoid arthritis of right wrist	M05.231	0	0.000	0	0.000	0	0.000	0	0.000	0.0000
	Rheumatoid vasculitis with rheumatoid arthritis of left wrist	M05.232	0	0.000	0	0.000	0	0.000	0	0.000	0.0000
	Rheumatoid vasculitis with rheumatoid arthritis of unspecified wrist	M05.239	0	0.000	0	0.000	0	0.000	0	0.000	0.0000
	Rheumatoid vasculitis with rheumatoid arthritis of hand	M05.24	0	0.000	0	0.000	0	0.000	0	0.000	0.0000

	Rheumatoid vasculitis with rheumatoid arthritis of right hand	M05.241	0	0.000	0	0.000	0	0.000	0	0.000	0.0000
	Rheumatoid vasculitis with rheumatoid arthritis of left hand	M05.242	0	0.000	0	0.000	0	0.000	0	0.000	0.0000
	Rheumatoid vasculitis with rheumatoid arthritis of unspecified hand	M05.249	0	0.000	0	0.000	0	0.000	0	0.000	0.0000
	Rheumatoid vasculitis with rheumatoid arthritis of hip	M05.25	0	0.000	0	0.000	0	0.000	0	0.000	0.0000
	Rheumatoid vasculitis with rheumatoid arthritis of right hip	M05.251	0	0.000	0	0.000	0	0.000	0	0.000	0.0000
	Rheumatoid vasculitis with rheumatoid arthritis of left hip	M05.252	0	0.000	0	0.000	0	0.000	0	0.000	0.0000
	Rheumatoid vasculitis with rheumatoid arthritis of unspecified hip	M05.259	0	0.000	0	0.000	0	0.000	0	0.000	0.0000
	Rheumatoid vasculitis with rheumatoid arthritis of knee	M05.26	0	0.000	0	0.000	0	0.000	0	0.000	0.0000
	Rheumatoid vasculitis with rheumatoid arthritis of right knee	M05.261	0	0.000	0	0.000	0	0.000	0	0.000	0.0000
	Rheumatoid vasculitis with rheumatoid arthritis of left knee	M05.262	0	0.000	0	0.000	0	0.000	0	0.000	0.0000
	Rheumatoid vasculitis with rheumatoid arthritis of unspecified knee	M05.269	0	0.000	0	0.000	0	0.000	0	0.000	0.0000
	Rheumatoid vasculitis with rheumatoid arthritis of ankle and foot	M05.27	0	0.000	0	0.000	0	0.000	0	0.000	0.0000
	Rheumatoid vasculitis with rheumatoid arthritis of right ankle and foot	M05.271	0	0.000	0	0.000	0	0.000	0	0.000	0.0000
	Rheumatoid vasculitis with rheumatoid arthritis of left ankle and foot	M05.272	0	0.000	0	0.000	0	0.000	0	0.000	0.0000
	Rheumatoid vasculitis with rheumatoid arthritis of unspecified ankle and foot	M05.279	0	0.000	0	0.000	0	0.000	0	0.000	0.0000
	Rheumatoid vasculitis with rheumatoid arthritis of multiple sites	M05.29	0	0.000	0	0.000	0	0.000	0	0.000	0.0000
	Rheumatoid heart disease with rheumatoid arthritis	M05.3	0	0.000	0	0.000	0	0.000	0	0.000	0.0000
	Rheumatoid heart disease with rheumatoid arthritis of unspecified site	M05.30	0	0.000	0	0.000	0	0.000	0	0.000	0.0000
	Rheumatoid heart disease with rheumatoid arthritis of shoulder	M05.31	0	0.000	0	0.000	0	0.000	0	0.000	0.0000
	Rheumatoid heart disease with rheumatoid arthritis of right shoulder	M05.311	0	0.000	0	0.000	0	0.000	0	0.000	0.0000
	Rheumatoid heart disease with rheumatoid arthritis of left shoulder	M05.312	0	0.000	0	0.000	0	0.000	0	0.000	0.0000
	Rheumatoid heart disease with rheumatoid arthritis of unspecified shoulder	M05.319	0	0.000	0	0.000	0	0.000	0	0.000	0.0000
	Rheumatoid heart disease with rheumatoid arthritis of elbow	M05.32	0	0.000	0	0.000	0	0.000	0	0.000	0.0000
	Rheumatoid heart disease with rheumatoid arthritis of right elbow	M05.321	0	0.000	0	0.000	0	0.000	0	0.000	0.0000
	Rheumatoid heart disease with rheumatoid arthritis of left elbow	M05.322	0	0.000	0	0.000	0	0.000	0	0.000	0.0000
	Rheumatoid heart disease with rheumatoid arthritis of	M05.329	0	0.000	0	0.000	0	0.000	0	0.000	0.0000

	unspecified elbow										
	Rheumatoid heart disease with rheumatoid arthritis of wrist	M05.33	0	0.000	0	0.000	0	0.000	0	0.000	0.0000
	Rheumatoid heart disease with rheumatoid arthritis of right wrist	M05.331	0	0.000	0	0.000	0	0.000	0	0.000	0.0000
	Rheumatoid heart disease with rheumatoid arthritis of left wrist	M05.332	0	0.000	0	0.000	0	0.000	0	0.000	0.0000
	Rheumatoid heart disease with rheumatoid arthritis of unspecified wrist	M05.339	0	0.000	0	0.000	0	0.000	0	0.000	0.0000
	Rheumatoid heart disease with rheumatoid arthritis of hand	M05.34	0	0.000	0	0.000	0	0.000	0	0.000	0.0000
	Rheumatoid heart disease with rheumatoid arthritis of right hand	M05.341	0	0.000	0	0.000	0	0.000	0	0.000	0.0000
	Rheumatoid heart disease with rheumatoid arthritis of left hand	M05.342	0	0.000	0	0.000	0	0.000	0	0.000	0.0000
	Rheumatoid heart disease with rheumatoid arthritis of unspecified hand	M05.349	0	0.000	0	0.000	0	0.000	0	0.000	0.0000
	Rheumatoid heart disease with rheumatoid arthritis of hip	M05.35	0	0.000	0	0.000	0	0.000	0	0.000	0.0000
	Rheumatoid heart disease with rheumatoid arthritis of right hip	M05.351	0	0.000	0	0.000	0	0.000	0	0.000	0.0000
	Rheumatoid heart disease with rheumatoid arthritis of left hip	M05.352	0	0.000	0	0.000	0	0.000	0	0.000	0.0000
	Rheumatoid heart disease with rheumatoid arthritis of unspecified hip	M05.359	0	0.000	0	0.000	0	0.000	0	0.000	0.0000
	Rheumatoid heart disease with rheumatoid arthritis of knee	M05.36	0	0.000	0	0.000	0	0.000	0	0.000	0.0000
	Rheumatoid heart disease with rheumatoid arthritis of right knee	M05.361	0	0.000	0	0.000	0	0.000	0	0.000	0.0000
	Rheumatoid heart disease with rheumatoid arthritis of left knee	M05.362	0	0.000	0	0.000	0	0.000	0	0.000	0.0000
	Rheumatoid heart disease with rheumatoid arthritis of unspecified knee	M05.369	0	0.000	0	0.000	0	0.000	0	0.000	0.0000
	Rheumatoid heart disease with rheumatoid arthritis of ankle and foot	M05.37	0	0.000	0	0.000	0	0.000	0	0.000	0.0000
	Rheumatoid heart disease with rheumatoid arthritis of right ankle and foot	M05.371	0	0.000	0	0.000	0	0.000	0	0.000	0.0000
	Rheumatoid heart disease with rheumatoid arthritis of left ankle and foot	M05.372	0	0.000	0	0.000	0	0.000	0	0.000	0.0000
	Rheumatoid heart disease with rheumatoid arthritis of unspecified ankle and foot	M05.379	0	0.000	0	0.000	0	0.000	0	0.000	0.0000
	Rheumatoid heart disease with rheumatoid arthritis of multiple sites	M05.39	0	0.000	0	0.000	0	0.000	0	0.000	0.0000
	Rheumatoid myopathy with rheumatoid arthritis	M05.4	0	0.000	0	0.000	0	0.000	0	0.000	0.0000
	Rheumatoid myopathy with rheumatoid arthritis of unspecified site	M05.40	0	0.000	0	0.000	0	0.000	0	0.000	0.0000
	Rheumatoid myopathy with rheumatoid arthritis of shoulder	M05.41	0	0.000	0	0.000	0	0.000	0	0.000	0.0000

	Rheumatoid myopathy with rheumatoid arthritis of right shoulder	M05.411	0	0.000	0	0.000	0	0.000	0	0.000	0.0000
	Rheumatoid myopathy with rheumatoid arthritis of left shoulder	M05.412	0	0.000	0	0.000	0	0.000	0	0.000	0.0000
	Rheumatoid myopathy with rheumatoid arthritis of unspecified shoulder	M05.419	0	0.000	0	0.000	0	0.000	0	0.000	0.0000
	Rheumatoid myopathy with rheumatoid arthritis of elbow	M05.42	0	0.000	0	0.000	0	0.000	0	0.000	0.0000
	Rheumatoid myopathy with rheumatoid arthritis of right elbow	M05.421	0	0.000	0	0.000	0	0.000	0	0.000	0.0000
	Rheumatoid myopathy with rheumatoid arthritis of left elbow	M05.422	0	0.000	0	0.000	0	0.000	0	0.000	0.0000
	Rheumatoid myopathy with rheumatoid arthritis of unspecified elbow	M05.429	0	0.000	0	0.000	0	0.000	0	0.000	0.0000
	Rheumatoid myopathy with rheumatoid arthritis of wrist	M05.43	0	0.000	0	0.000	0	0.000	0	0.000	0.0000
	Rheumatoid myopathy with rheumatoid arthritis of right wrist	M05.431	0	0.000	0	0.000	0	0.000	0	0.000	0.0000
	Rheumatoid myopathy with rheumatoid arthritis of left wrist	M05.432	0	0.000	0	0.000	0	0.000	0	0.000	0.0000
	Rheumatoid myopathy with rheumatoid arthritis of unspecified wrist	M05.439	0	0.000	0	0.000	0	0.000	0	0.000	0.0000
	Rheumatoid myopathy with rheumatoid arthritis of hand	M05.44	0	0.000	0	0.000	0	0.000	0	0.000	0.0000
	Rheumatoid myopathy with rheumatoid arthritis of right hand	M05.441	0	0.000	0	0.000	0	0.000	0	0.000	0.0000
	Rheumatoid myopathy with rheumatoid arthritis of left hand	M05.442	0	0.000	0	0.000	0	0.000	0	0.000	0.0000
	Rheumatoid myopathy with rheumatoid arthritis of unspecified hand	M05.449	0	0.000	0	0.000	0	0.000	0	0.000	0.0000
	Rheumatoid myopathy with rheumatoid arthritis of hip	M05.45	0	0.000	0	0.000	0	0.000	0	0.000	0.0000
	Rheumatoid myopathy with rheumatoid arthritis of right hip	M05.451	0	0.000	0	0.000	0	0.000	0	0.000	0.0000
	Rheumatoid myopathy with rheumatoid arthritis of left hip	M05.452	0	0.000	0	0.000	0	0.000	0	0.000	0.0000
	Rheumatoid myopathy with rheumatoid arthritis of unspecified hip	M05.459	0	0.000	0	0.000	0	0.000	0	0.000	0.0000
	Rheumatoid myopathy with rheumatoid arthritis of knee	M05.46	0	0.000	0	0.000	0	0.000	0	0.000	0.0000
	Rheumatoid myopathy with rheumatoid arthritis of right knee	M05.461	0	0.000	0	0.000	0	0.000	0	0.000	0.0000
	Rheumatoid myopathy with rheumatoid arthritis of left knee	M05.462	0	0.000	0	0.000	0	0.000	0	0.000	0.0000
	Rheumatoid myopathy with rheumatoid arthritis of unspecified knee	M05.469	0	0.000	0	0.000	0	0.000	0	0.000	0.0000
	Rheumatoid myopathy with rheumatoid arthritis of ankle and foot	M05.47	0	0.000	0	0.000	0	0.000	0	0.000	0.0000
	Rheumatoid myopathy with rheumatoid arthritis of right ankle and foot	M05.471	0	0.000	0	0.000	0	0.000	0	0.000	0.0000
	Rheumatoid myopathy with rheumatoid arthritis of left ankle and foot	M05.472	0	0.000	0	0.000	0	0.000	0	0.000	0.0000

	Rheumatoid myopathy with rheumatoid arthritis of unspecified ankle and foot	M05.479	0	0.000	0	0.000	0	0.000	0	0.000	0.0000
	Rheumatoid myopathy with rheumatoid arthritis of multiple sites	M05.49	0	0.000	0	0.000	0	0.000	0	0.000	0.0000
	Rheumatoid polyneuropathy with rheumatoid arthritis	M05.5	0	0.000	0	0.000	0	0.000	0	0.000	0.0000
	Rheumatoid polyneuropathy with rheumatoid arthritis of unspecified site	M05.50	0	0.000	0	0.000	0	0.000	0	0.000	0.0000
	Rheumatoid polyneuropathy with rheumatoid arthritis of shoulder	M05.51	0	0.000	0	0.000	0	0.000	0	0.000	0.0000
	Rheumatoid polyneuropathy with rheumatoid arthritis of right shoulder	M05.511	0	0.000	0	0.000	0	0.000	0	0.000	0.0000
	Rheumatoid polyneuropathy with rheumatoid arthritis of left shoulder	M05.512	0	0.000	0	0.000	0	0.000	0	0.000	0.0000
	Rheumatoid polyneuropathy with rheumatoid arthritis of unspecified shoulder	M05.519	0	0.000	0	0.000	0	0.000	0	0.000	0.0000
	Rheumatoid polyneuropathy with rheumatoid arthritis of elbow	M05.52	0	0.000	0	0.000	0	0.000	0	0.000	0.0000
	Rheumatoid polyneuropathy with rheumatoid arthritis of right elbow	M05.521	0	0.000	0	0.000	0	0.000	0	0.000	0.0000
	Rheumatoid polyneuropathy with rheumatoid arthritis of left elbow	M05.522	0	0.000	0	0.000	0	0.000	0	0.000	0.0000
	Rheumatoid polyneuropathy with rheumatoid arthritis of unspecified elbow	M05.529	0	0.000	0	0.000	0	0.000	0	0.000	0.0000
	Rheumatoid polyneuropathy with rheumatoid arthritis of wrist	M05.53	0	0.000	0	0.000	0	0.000	0	0.000	0.0000
	Rheumatoid polyneuropathy with rheumatoid arthritis of right wrist	M05.531	0	0.000	0	0.000	0	0.000	0	0.000	0.0000
	Rheumatoid polyneuropathy with rheumatoid arthritis of left wrist	M05.532	0	0.000	0	0.000	0	0.000	0	0.000	0.0000
	Rheumatoid polyneuropathy with rheumatoid arthritis of unspecified wrist	M05.539	0	0.000	0	0.000	0	0.000	0	0.000	0.0000
	Rheumatoid polyneuropathy with rheumatoid arthritis of hand	M05.54	0	0.000	0	0.000	0	0.000	0	0.000	0.0000
	Rheumatoid polyneuropathy with rheumatoid arthritis of right hand	M05.541	0	0.000	0	0.000	0	0.000	0	0.000	0.0000
	Rheumatoid polyneuropathy with rheumatoid arthritis of left hand	M05.542	0	0.000	0	0.000	0	0.000	0	0.000	0.0000
	Rheumatoid polyneuropathy with rheumatoid arthritis of unspecified hand	M05.549	0	0.000	0	0.000	0	0.000	0	0.000	0.0000
	Rheumatoid polyneuropathy with rheumatoid arthritis of hip	M05.55	0	0.000	0	0.000	0	0.000	0	0.000	0.0000
	Rheumatoid polyneuropathy with rheumatoid arthritis of right hip	M05.551	0	0.000	0	0.000	0	0.000	0	0.000	0.0000
	Rheumatoid polyneuropathy with rheumatoid arthritis of left hip	M05.552	0	0.000	0	0.000	0	0.000	0	0.000	0.0000
	Rheumatoid polyneuropathy with rheumatoid arthritis of unspecified hip	M05.559	0	0.000	0	0.000	0	0.000	0	0.000	0.0000

	Rheumatoid polyneuropathy with rheumatoid arthritis of knee	M05.56	0	0.000	0	0.000	0	0.000	0	0.000	0.0000
	Rheumatoid polyneuropathy with rheumatoid arthritis of right knee	M05.561	0	0.000	0	0.000	0	0.000	0	0.000	0.0000
	Rheumatoid polyneuropathy with rheumatoid arthritis of left knee	M05.562	0	0.000	0	0.000	0	0.000	0	0.000	0.0000
	Rheumatoid polyneuropathy with rheumatoid arthritis of unspecified knee	M05.569	0	0.000	0	0.000	0	0.000	0	0.000	0.0000
	Rheumatoid polyneuropathy with rheumatoid arthritis of ankle and foot	M05.57	0	0.000	0	0.000	0	0.000	0	0.000	0.0000
	Rheumatoid polyneuropathy with rheumatoid arthritis of right ankle and foot	M05.571	0	0.000	0	0.000	0	0.000	0	0.000	0.0000
	Rheumatoid polyneuropathy with rheumatoid arthritis of left ankle and foot	M05.572	0	0.000	0	0.000	0	0.000	0	0.000	0.0000
	Rheumatoid polyneuropathy with rheumatoid arthritis of unspecified ankle and foot	M05.579	0	0.000	0	0.000	0	0.000	0	0.000	0.0000
	Rheumatoid polyneuropathy with rheumatoid arthritis of multiple sites	M05.59	0	0.000	0	0.000	0	0.000	0	0.000	0.0000
	Rheumatoid arthritis with involvement of other organs and systems	M05.6	0	0.000	0	0.000	0	0.000	0	0.000	0.0000
	Rheumatoid arthritis of unspecified site with involvement of other organs and systems	M05.60	0	0.000	0	0.000	0	0.000	0	0.000	0.0000
	Rheumatoid arthritis of shoulder with involvement of other organs and systems	M05.61	0	0.000	0	0.000	0	0.000	0	0.000	0.0000
	Rheumatoid arthritis of right shoulder with involvement of other organs and systems	M05.611	0	0.000	0	0.000	0	0.000	0	0.000	0.0000
	Rheumatoid arthritis of left shoulder with involvement of other organs and systems	M05.612	0	0.000	0	0.000	0	0.000	0	0.000	0.0000
	Rheumatoid arthritis of unspecified shoulder with involvement of other organs and systems	M05.619	0	0.000	0	0.000	0	0.000	0	0.000	0.0000
	Rheumatoid arthritis of elbow with involvement of other organs and systems	M05.62	0	0.000	0	0.000	0	0.000	0	0.000	0.0000
	Rheumatoid arthritis of right elbow with involvement of other organs and systems	M05.621	0	0.000	0	0.000	0	0.000	0	0.000	0.0000
	Rheumatoid arthritis of left elbow with involvement of other organs and systems	M05.622	0	0.000	0	0.000	0	0.000	0	0.000	0.0000
	Rheumatoid arthritis of unspecified elbow with involvement of other organs and systems	M05.629	0	0.000	0	0.000	0	0.000	0	0.000	0.0000
	Rheumatoid arthritis of wrist with involvement of other organs and systems	M05.63	0	0.000	0	0.000	0	0.000	0	0.000	0.0000
	Rheumatoid arthritis of right wrist with involvement of other organs and systems	M05.631	0	0.000	0	0.000	0	0.000	0	0.000	0.0000
	Rheumatoid arthritis of left wrist with involvement of other organs and systems	M05.632	0	0.000	0	0.000	0	0.000	0	0.000	0.0000
	Rheumatoid arthritis of unspecified wrist with involvement of other organs and systems	M05.639	0	0.000	0	0.000	0	0.000	0	0.000	0.0000
	Rheumatoid arthritis of hand with involvement of other	M05.64	0	0.000	0	0.000	0	0.000	0	0.000	0.0000

	organs and systems										
	Rheumatoid arthritis of right hand with involvement of other organs and systems	M05.641	0	0.000	0	0.000	0	0.000	0	0.000	0.0000
	Rheumatoid arthritis of left hand with involvement of other organs and systems	M05.642	0	0.000	0	0.000	0	0.000	0	0.000	0.0000
	Rheumatoid arthritis of unspecified hand with involvement of other organs and systems	M05.649	0	0.000	0	0.000	0	0.000	0	0.000	0.0000
	Rheumatoid arthritis of hip with involvement of other organs and systems	M05.65	0	0.000	0	0.000	0	0.000	0	0.000	0.0000
	Rheumatoid arthritis of right hip with involvement of other organs and systems	M05.651	0	0.000	0	0.000	0	0.000	0	0.000	0.0000
	Rheumatoid arthritis of left hip with involvement of other organs and systems	M05.652	0	0.000	0	0.000	0	0.000	0	0.000	0.0000
	Rheumatoid arthritis of unspecified hip with involvement of other organs and systems	M05.659	0	0.000	0	0.000	0	0.000	0	0.000	0.0000
	Rheumatoid arthritis of knee with involvement of other organs and systems	M05.66	0	0.000	0	0.000	0	0.000	0	0.000	0.0000
	Rheumatoid arthritis of right knee with involvement of other organs and systems	M05.661	0	0.000	0	0.000	0	0.000	0	0.000	0.0000
	Rheumatoid arthritis of left knee with involvement of other organs and systems	M05.662	0	0.000	0	0.000	0	0.000	0	0.000	0.0000
	Rheumatoid arthritis of unspecified knee with involvement of other organs and systems	M05.669	0	0.000	0	0.000	0	0.000	0	0.000	0.0000
	Rheumatoid arthritis of ankle and foot with involvement of other organs and systems	M05.67	0	0.000	0	0.000	0	0.000	0	0.000	0.0000
	Rheumatoid arthritis of right ankle and foot with involvement of other organs and systems	M05.671	0	0.000	0	0.000	0	0.000	0	0.000	0.0000
	Rheumatoid arthritis of left ankle and foot with involvement of other organs and systems	M05.672	0	0.000	0	0.000	0	0.000	0	0.000	0.0000
	Rheumatoid arthritis of unspecified ankle and foot with involvement of other organs and systems	M05.679	0	0.000	0	0.000	0	0.000	0	0.000	0.0000
	Rheumatoid arthritis of multiple sites with involvement of other organs and systems	M05.69	0	0.000	0	0.000	0	0.000	0	0.000	0.0000
	Rheumatoid arthritis with rheumatoid factor without organ or systems involvement	M05.7	0	0.000	0	0.000	0	0.000	0	0.000	0.0000
	Rheumatoid arthritis with rheumatoid factor of unspecified site without organ or systems involvement	M05.70	0	0.000	0	0.000	0	0.000	0	0.000	0.0000
	Rheumatoid arthritis with rheumatoid factor of shoulder without organ or systems involvement	M05.71	0	0.000	0	0.000	0	0.000	0	0.000	0.0000
	Rheumatoid arthritis with rheumatoid factor of right shoulder without organ or systems involvement	M05.711	0	0.000	0	0.000	0	0.000	0	0.000	0.0000
	Rheumatoid arthritis with rheumatoid factor of left shoulder without organ or systems involvement	M05.712	0	0.000	0	0.000	0	0.000	0	0.000	0.0000
	Rheumatoid arthritis with rheumatoid factor of unspecified shoulder without organ or systems involvement	M05.719	0	0.000	0	0.000	0	0.000	0	0.000	0.0000
	Rheumatoid arthritis with rheumatoid factor of elbow without organ or systems involvement	M05.72	0	0.000	0	0.000	0	0.000	0	0.000	0.0000

	Rheumatoid arthritis with rheumatoid factor of right elbow without organ or systems involvement	M05.721	0	0.000	0	0.000	0	0.000	0	0.000	0.0000
	Rheumatoid arthritis with rheumatoid factor of left elbow without organ or systems involvement	M05.722	0	0.000	0	0.000	0	0.000	0	0.000	0.0000
	Rheumatoid arthritis with rheumatoid factor of unspecified elbow without organ or systems involvement	M05.729	0	0.000	0	0.000	0	0.000	0	0.000	0.0000
	Rheumatoid arthritis with rheumatoid factor of wrist without organ or systems involvement	M05.73	0	0.000	0	0.000	0	0.000	0	0.000	0.0000
	Rheumatoid arthritis with rheumatoid factor of right wrist without organ or systems involvement	M05.731	0	0.000	0	0.000	0	0.000	0	0.000	0.0000
	Rheumatoid arthritis with rheumatoid factor of left wrist without organ or systems involvement	M05.732	0	0.000	0	0.000	0	0.000	0	0.000	0.0000
	Rheumatoid arthritis with rheumatoid factor of unspecified wrist without organ or systems involvement	M05.739	0	0.000	0	0.000	0	0.000	0	0.000	0.0000
	Rheumatoid arthritis with rheumatoid factor of hand without organ or systems involvement	M05.74	0	0.000	0	0.000	0	0.000	0	0.000	0.0000
	Rheumatoid arthritis with rheumatoid factor of right hand without organ or systems involvement	M05.741	0	0.000	0	0.000	0	0.000	0	0.000	0.0000
	Rheumatoid arthritis with rheumatoid factor of left hand without organ or systems involvement	M05.742	0	0.000	0	0.000	0	0.000	0	0.000	0.0000
	Rheumatoid arthritis with rheumatoid factor of unspecified hand without organ or systems involvement	M05.749	0	0.000	0	0.000	0	0.000	0	0.000	0.0000
	Rheumatoid arthritis with rheumatoid factor of hip without organ or systems involvement	M05.75	0	0.000	0	0.000	0	0.000	0	0.000	0.0000
	Rheumatoid arthritis with rheumatoid factor of right hip without organ or systems involvement	M05.751	0	0.000	0	0.000	0	0.000	0	0.000	0.0000
	Rheumatoid arthritis with rheumatoid factor of left hip without organ or systems involvement	M05.752	0	0.000	0	0.000	0	0.000	0	0.000	0.0000
	Rheumatoid arthritis with rheumatoid factor of unspecified hip without organ or systems involvement	M05.759	0	0.000	0	0.000	0	0.000	0	0.000	0.0000
	Rheumatoid arthritis with rheumatoid factor of knee without organ or systems involvement	M05.76	0	0.000	0	0.000	0	0.000	0	0.000	0.0000
	Rheumatoid arthritis with rheumatoid factor of right knee without organ or systems involvement	M05.761	0	0.000	0	0.000	0	0.000	0	0.000	0.0000
	Rheumatoid arthritis with rheumatoid factor of left knee without organ or systems involvement	M05.762	0	0.000	0	0.000	0	0.000	0	0.000	0.0000
	Rheumatoid arthritis with rheumatoid factor of unspecified knee without organ or systems involvement	M05.769	0	0.000	0	0.000	0	0.000	0	0.000	0.0000
	Rheumatoid arthritis with rheumatoid factor of ankle and foot without organ or systems involvement	M05.77	0	0.000	0	0.000	0	0.000	0	0.000	0.0000
	Rheumatoid arthritis with rheumatoid factor of right ankle and foot without organ or systems involvement	M05.771	0	0.000	0	0.000	0	0.000	0	0.000	0.0000
	Rheumatoid arthritis with rheumatoid factor of left ankle and foot without organ or systems involvement	M05.772	0	0.000	0	0.000	0	0.000	0	0.000	0.0000
	Rheumatoid arthritis with rheumatoid factor of unspecified ankle and foot without organ or systems involvement	M05.779	0	0.000	0	0.000	0	0.000	0	0.000	0.0000
	Rheumatoid arthritis with rheumatoid factor of multiple sites	M05.79	0	0.000	0	0.000	0	0.000	0	0.000	0.0000

	without organ or systems involvement										
	Rheumatoid arthritis with rheumatoid factor of other specified site without organ or systems involvement	M05.7A	0	0.000	0	0.000	0	0.000	0	0.000	0.0000
	Other rheumatoid arthritis with rheumatoid factor	M05.8	0	0.000	0	0.000	2	0.074	2	0.007	0.0000
	Other rheumatoid arthritis with rheumatoid factor of unspecified site	M05.80	0	0.000	0	0.000	0	0.000	0	0.000	0.0000
	Other rheumatoid arthritis with rheumatoid factor of shoulder	M05.81	0	0.000	0	0.000	0	0.000	0	0.000	0.0000
	Other rheumatoid arthritis with rheumatoid factor of right shoulder	M05.811	0	0.000	0	0.000	0	0.000	0	0.000	0.0000
	Other rheumatoid arthritis with rheumatoid factor of left shoulder	M05.812	0	0.000	0	0.000	0	0.000	0	0.000	0.0000
	Other rheumatoid arthritis with rheumatoid factor of unspecified shoulder	M05.819	0	0.000	0	0.000	0	0.000	0	0.000	0.0000
	Other rheumatoid arthritis with rheumatoid factor of elbow	M05.82	0	0.000	0	0.000	0	0.000	0	0.000	0.0000
	Other rheumatoid arthritis with rheumatoid factor of right elbow	M05.821	0	0.000	0	0.000	0	0.000	0	0.000	0.0000
	Other rheumatoid arthritis with rheumatoid factor of left elbow	M05.822	0	0.000	0	0.000	0	0.000	0	0.000	0.0000
	Other rheumatoid arthritis with rheumatoid factor of unspecified elbow	M05.829	0	0.000	0	0.000	0	0.000	0	0.000	0.0000
	Other rheumatoid arthritis with rheumatoid factor of wrist	M05.83	0	0.000	0	0.000	0	0.000	0	0.000	0.0000
	Other rheumatoid arthritis with rheumatoid factor of right wrist	M05.831	0	0.000	0	0.000	0	0.000	0	0.000	0.0000
	Other rheumatoid arthritis with rheumatoid factor of left wrist	M05.832	0	0.000	0	0.000	0	0.000	0	0.000	0.0000
	Other rheumatoid arthritis with rheumatoid factor of unspecified wrist	M05.839	0	0.000	0	0.000	0	0.000	0	0.000	0.0000
	Other rheumatoid arthritis with rheumatoid factor of hand	M05.84	0	0.000	0	0.000	0	0.000	0	0.000	0.0000
	Other rheumatoid arthritis with rheumatoid factor of right hand	M05.841	0	0.000	0	0.000	0	0.000	0	0.000	0.0000
	Other rheumatoid arthritis with rheumatoid factor of left hand	M05.842	0	0.000	0	0.000	0	0.000	0	0.000	0.0000
	Other rheumatoid arthritis with rheumatoid factor of unspecified hand	M05.849	0	0.000	0	0.000	0	0.000	0	0.000	0.0000
	Other rheumatoid arthritis with rheumatoid factor of hip	M05.85	0	0.000	0	0.000	0	0.000	0	0.000	0.0000
	Other rheumatoid arthritis with rheumatoid factor of right hip	M05.851	0	0.000	0	0.000	0	0.000	0	0.000	0.0000
	Other rheumatoid arthritis with rheumatoid factor of left hip	M05.852	0	0.000	0	0.000	0	0.000	0	0.000	0.0000
	Other rheumatoid arthritis with rheumatoid factor of unspecified hip	M05.859	0	0.000	0	0.000	0	0.000	0	0.000	0.0000
	Other rheumatoid arthritis with rheumatoid factor of knee	M05.86	0	0.000	0	0.000	0	0.000	0	0.000	0.0000
	Other rheumatoid arthritis with rheumatoid factor of right knee	M05.861	0	0.000	0	0.000	0	0.000	0	0.000	0.0000

	Other rheumatoid arthritis with rheumatoid factor of left knee	M05.862	0	0.000	0	0.000	0	0.000	0	0.000	0.0000
	Other rheumatoid arthritis with rheumatoid factor of unspecified knee	M05.869	0	0.000	0	0.000	0	0.000	0	0.000	0.0000
	Other rheumatoid arthritis with rheumatoid factor of ankle and foot	M05.87	0	0.000	0	0.000	0	0.000	0	0.000	0.0000
	Other rheumatoid arthritis with rheumatoid factor of right ankle and foot	M05.871	0	0.000	0	0.000	0	0.000	0	0.000	0.0000
	Other rheumatoid arthritis with rheumatoid factor of left ankle and foot	M05.872	0	0.000	0	0.000	0	0.000	0	0.000	0.0000
	Other rheumatoid arthritis with rheumatoid factor of unspecified ankle and foot	M05.879	0	0.000	0	0.000	0	0.000	0	0.000	0.0000
	Other rheumatoid arthritis with rheumatoid factor of multiple sites	M05.89	0	0.000	0	0.000	0	0.000	0	0.000	0.0000
	Other rheumatoid arthritis with rheumatoid factor of other specified site	M05.8A	0	0.000	0	0.000	0	0.000	0	0.000	0.0000
	Rheumatoid arthritis with rheumatoid factor. unspecified	M05.9	4	0.025	6	0.051	2	0.074	12	0.040	0.4197
	**Other rheumatoid arthritis**	**M06**	0	0.000	0	0.000	0	0.000	0	0.000	0.0000
	Rheumatoid arthritis without rheumatoid factor	M06.0	11	0.069	7	0.060	3	0.111	21	0.069	0.9503
	Rheumatoid arthritis without rheumatoid factor. unspecified site	M06.00	0	0.000	0	0.000	0	0.000	0	0.000	0.0000
	Rheumatoid arthritis without rheumatoid factor. shoulder	M06.01	0	0.000	0	0.000	0	0.000	0	0.000	0.0000
	Rheumatoid arthritis without rheumatoid factor. right shoulder	M06.011	0	0.000	0	0.000	0	0.000	0	0.000	0.0000
	Rheumatoid arthritis without rheumatoid factor. left shoulder	M06.012	0	0.000	0	0.000	0	0.000	0	0.000	0.0000
	Rheumatoid arthritis without rheumatoid factor. unspecified shoulder	M06.019	0	0.000	0	0.000	0	0.000	0	0.000	0.0000
	Rheumatoid arthritis without rheumatoid factor. elbow	M06.02	0	0.000	0	0.000	0	0.000	0	0.000	0.0000
	Rheumatoid arthritis without rheumatoid factor. right elbow	M06.021	0	0.000	0	0.000	0	0.000	0	0.000	0.0000
	Rheumatoid arthritis without rheumatoid factor. left elbow	M06.022	0	0.000	0	0.000	0	0.000	0	0.000	0.0000
	Rheumatoid arthritis without rheumatoid factor. unspecified elbow	M06.029	0	0.000	0	0.000	0	0.000	0	0.000	0.0000
	Rheumatoid arthritis without rheumatoid factor. wrist	M06.03	0	0.000	0	0.000	0	0.000	0	0.000	0.0000
	Rheumatoid arthritis without rheumatoid factor. right wrist	M06.031	0	0.000	0	0.000	0	0.000	0	0.000	0.0000
	Rheumatoid arthritis without rheumatoid factor. left wrist	M06.032	0	0.000	0	0.000	0	0.000	0	0.000	0.0000
	Rheumatoid arthritis without rheumatoid factor. unspecified wrist	M06.039	0	0.000	0	0.000	0	0.000	0	0.000	0.0000
	Rheumatoid arthritis without rheumatoid factor. hand	M06.04	0	0.000	0	0.000	0	0.000	0	0.000	0.0000
	Rheumatoid arthritis without rheumatoid factor. right hand	M06.041	0	0.000	0	0.000	0	0.000	0	0.000	0.0000

	Rheumatoid arthritis without rheumatoid factor. left hand	M06.042	0	0.000	0	0.000	0	0.000	0	0.000	0.0000
	Rheumatoid arthritis without rheumatoid factor. unspecified hand	M06.049	0	0.000	0	0.000	0	0.000	0	0.000	0.0000
	Rheumatoid arthritis without rheumatoid factor. hip	M06.05	0	0.000	0	0.000	0	0.000	0	0.000	0.0000
	Rheumatoid arthritis without rheumatoid factor. right hip	M06.051	0	0.000	0	0.000	0	0.000	0	0.000	0.0000
	Rheumatoid arthritis without rheumatoid factor. left hip	M06.052	0	0.000	0	0.000	0	0.000	0	0.000	0.0000
	Rheumatoid arthritis without rheumatoid factor. unspecified hip	M06.059	0	0.000	0	0.000	0	0.000	0	0.000	0.0000
	Rheumatoid arthritis without rheumatoid factor. knee	M06.06	0	0.000	0	0.000	0	0.000	0	0.000	0.0000
	Rheumatoid arthritis without rheumatoid factor. right knee	M06.061	0	0.000	0	0.000	0	0.000	0	0.000	0.0000
	Rheumatoid arthritis without rheumatoid factor. left knee	M06.062	0	0.000	0	0.000	0	0.000	0	0.000	0.0000
	Rheumatoid arthritis without rheumatoid factor. unspecified knee	M06.069	0	0.000	0	0.000	0	0.000	0	0.000	0.0000
	Rheumatoid arthritis without rheumatoid factor. ankle and foot	M06.07	0	0.000	0	0.000	0	0.000	0	0.000	0.0000
	Rheumatoid arthritis without rheumatoid factor. right ankle and foot	M06.071	0	0.000	0	0.000	0	0.000	0	0.000	0.0000
	Rheumatoid arthritis without rheumatoid factor. left ankle and foot	M06.072	0	0.000	0	0.000	0	0.000	0	0.000	0.0000
	Rheumatoid arthritis without rheumatoid factor. unspecified ankle and foot	M06.079	0	0.000	0	0.000	0	0.000	0	0.000	0.0000
	Rheumatoid arthritis without rheumatoid factor. vertebrae	M06.08	0	0.000	0	0.000	0	0.000	0	0.000	0.0000
	Rheumatoid arthritis without rheumatoid factor. multiple sites	M06.09	0	0.000	0	0.000	0	0.000	0	0.000	0.0000
	Rheumatoid arthritis without rheumatoid factor. other specified site	M06.0A	0	0.000	0	0.000	0	0.000	0	0.000	0.0000
	Adult-onset Still's disease	M06.1	0	0.000	0	0.000	0	0.000	0	0.000	0.0000
	Rheumatoid bursitis	M06.2	0	0.000	0	0.000	0	0.000	0	0.000	0.0000
	Rheumatoid bursitis. unspecified site	M06.20	0	0.000	0	0.000	0	0.000	0	0.000	0.0000
	Rheumatoid bursitis. shoulder	M06.21	0	0.000	0	0.000	0	0.000	0	0.000	0.0000
	Rheumatoid bursitis. right shoulder	M06.211	0	0.000	0	0.000	0	0.000	0	0.000	0.0000
	Rheumatoid bursitis. left shoulder	M06.212	0	0.000	0	0.000	0	0.000	0	0.000	0.0000
	Rheumatoid bursitis. unspecified shoulder	M06.219	0	0.000	0	0.000	0	0.000	0	0.000	0.0000
	Rheumatoid bursitis. elbow	M06.22	0	0.000	0	0.000	0	0.000	0	0.000	0.0000
	Rheumatoid bursitis. right elbow	M06.221	0	0.000	0	0.000	0	0.000	0	0.000	0.0000
	Rheumatoid bursitis. left elbow	M06.222	0	0.000	0	0.000	0	0.000	0	0.000	0.0000

	Rheumatoid bursitis. unspecified elbow	M06.229	0	0.000	0	0.000	0	0.000	0	0.000	0.0000
	Rheumatoid bursitis. wrist	M06.23	0	0.000	0	0.000	0	0.000	0	0.000	0.0000
	Rheumatoid bursitis. right wrist	M06.231	0	0.000	0	0.000	0	0.000	0	0.000	0.0000
	Rheumatoid bursitis. left wrist	M06.232	0	0.000	0	0.000	0	0.000	0	0.000	0.0000
	Rheumatoid bursitis. unspecified wrist	M06.239	0	0.000	0	0.000	0	0.000	0	0.000	0.0000
	Rheumatoid bursitis. hand	M06.24	0	0.000	0	0.000	0	0.000	0	0.000	0.0000
	Rheumatoid bursitis. right hand	M06.241	0	0.000	0	0.000	0	0.000	0	0.000	0.0000
	Rheumatoid bursitis. left hand	M06.242	0	0.000	0	0.000	0	0.000	0	0.000	0.0000
	Rheumatoid bursitis. unspecified hand	M06.249	0	0.000	0	0.000	0	0.000	0	0.000	0.0000
	Rheumatoid bursitis. hip	M06.25	0	0.000	0	0.000	0	0.000	0	0.000	0.0000
	Rheumatoid bursitis. right hip	M06.251	0	0.000	0	0.000	0	0.000	0	0.000	0.0000
	Rheumatoid bursitis. left hip	M06.252	0	0.000	0	0.000	0	0.000	0	0.000	0.0000
	Rheumatoid bursitis. unspecified hip	M06.259	0	0.000	0	0.000	0	0.000	0	0.000	0.0000
	Rheumatoid bursitis. knee	M06.26	0	0.000	0	0.000	0	0.000	0	0.000	0.0000
	Rheumatoid bursitis. right knee	M06.261	0	0.000	0	0.000	0	0.000	0	0.000	0.0000
	Rheumatoid bursitis. left knee	M06.262	0	0.000	0	0.000	0	0.000	0	0.000	0.0000
	Rheumatoid bursitis. unspecified knee	M06.269	0	0.000	0	0.000	0	0.000	0	0.000	0.0000
	Rheumatoid bursitis. ankle and foot	M06.27	0	0.000	0	0.000	0	0.000	0	0.000	0.0000
	Rheumatoid bursitis. right ankle and foot	M06.271	0	0.000	0	0.000	0	0.000	0	0.000	0.0000
	Rheumatoid bursitis. left ankle and foot	M06.272	0	0.000	0	0.000	0	0.000	0	0.000	0.0000
	Rheumatoid bursitis. unspecified ankle and foot	M06.279	0	0.000	0	0.000	0	0.000	0	0.000	0.0000
	Rheumatoid bursitis. vertebrae	M06.28	0	0.000	0	0.000	0	0.000	0	0.000	0.0000
	Rheumatoid bursitis. multiple sites	M06.29	0	0.000	0	0.000	0	0.000	0	0.000	0.0000
	Rheumatoid nodule	M06.3	5	0.031	6	0.051	2	0.074	13	0.043	0.6096
	Rheumatoid nodule. unspecified site	M06.30	0	0.000	0	0.000	0	0.000	0	0.000	0.0000
	Rheumatoid nodule. shoulder	M06.31	0	0.000	0	0.000	0	0.000	0	0.000	0.0000
	Rheumatoid nodule. right shoulder	M06.311	0	0.000	0	0.000	0	0.000	0	0.000	0.0000
	Rheumatoid nodule. left shoulder	M06.312	0	0.000	0	0.000	0	0.000	0	0.000	0.0000
	Rheumatoid nodule. unspecified shoulder	M06.319	0	0.000	0	0.000	0	0.000	0	0.000	0.0000

	Rheumatoid nodule. elbow	M06.32	0	0.000	0	0.000	0	0.000	0	0.000	0.0000
	Rheumatoid nodule. right elbow	M06.321	0	0.000	0	0.000	0	0.000	0	0.000	0.0000
	Rheumatoid nodule. left elbow	M06.322	0	0.000	0	0.000	0	0.000	0	0.000	0.0000
	Rheumatoid nodule. unspecified elbow	M06.329	0	0.000	0	0.000	0	0.000	0	0.000	0.0000
	Rheumatoid nodule. wrist	M06.33	0	0.000	0	0.000	0	0.000	0	0.000	0.0000
	Rheumatoid nodule. right wrist	M06.331	0	0.000	0	0.000	0	0.000	0	0.000	0.0000
	Rheumatoid nodule. left wrist	M06.332	0	0.000	0	0.000	0	0.000	0	0.000	0.0000
	Rheumatoid nodule. unspecified wrist	M06.339	0	0.000	0	0.000	0	0.000	0	0.000	0.0000
	Rheumatoid nodule. hand	M06.34	0	0.000	0	0.000	0	0.000	0	0.000	0.0000
	Rheumatoid nodule. right hand	M06.341	0	0.000	0	0.000	0	0.000	0	0.000	0.0000
	Rheumatoid nodule. left hand	M06.342	0	0.000	0	0.000	0	0.000	0	0.000	0.0000
	Rheumatoid nodule. unspecified hand	M06.349	0	0.000	0	0.000	0	0.000	0	0.000	0.0000
	Rheumatoid nodule. hip	M06.35	0	0.000	0	0.000	0	0.000	0	0.000	0.0000
	Rheumatoid nodule. right hip	M06.351	0	0.000	0	0.000	0	0.000	0	0.000	0.0000
	Rheumatoid nodule. left hip	M06.352	0	0.000	0	0.000	0	0.000	0	0.000	0.0000
	Rheumatoid nodule. unspecified hip	M06.359	0	0.000	0	0.000	0	0.000	0	0.000	0.0000
	Rheumatoid nodule. knee	M06.36	0	0.000	0	0.000	0	0.000	0	0.000	0.0000
	Rheumatoid nodule. right knee	M06.361	0	0.000	0	0.000	0	0.000	0	0.000	0.0000
	Rheumatoid nodule. left knee	M06.362	0	0.000	0	0.000	0	0.000	0	0.000	0.0000
	Rheumatoid nodule. unspecified knee	M06.369	0	0.000	0	0.000	0	0.000	0	0.000	0.0000
	Rheumatoid nodule. ankle and foot	M06.37	0	0.000	0	0.000	0	0.000	0	0.000	0.0000
	Rheumatoid nodule. right ankle and foot	M06.371	0	0.000	0	0.000	0	0.000	0	0.000	0.0000
	Rheumatoid nodule. left ankle and foot	M06.372	0	0.000	0	0.000	0	0.000	0	0.000	0.0000
	Rheumatoid nodule. unspecified ankle and foot	M06.379	0	0.000	0	0.000	0	0.000	0	0.000	0.0000
	Rheumatoid nodule. vertebrae	M06.38	0	0.000	0	0.000	0	0.000	0	0.000	0.0000
	Rheumatoid nodule. multiple sites	M06.39	0	0.000	0	0.000	0	0.000	0	0.000	0.0000
	Inflammatory polyarthropathy	M06.4	20	0.126	15	0.128	5	0.186	40	0.132	0.9081
	Other specified rheumatoid arthritis	M06.8	13	0.082	11	0.094	2	0.074	26	0.086	0.8929
	Other specified rheumatoid arthritis. unspecified site	M06.80	0	0.000	0	0.000	0	0.000	0	0.000	0.0000

	Other specified rheumatoid arthritis. shoulder	M06.81	0	0.000	0	0.000	0	0.000	0	0.000	0.0000
	Other specified rheumatoid arthritis. right shoulder	M06.811	0	0.000	0	0.000	0	0.000	0	0.000	0.0000
	Other specified rheumatoid arthritis. left shoulder	M06.812	0	0.000	0	0.000	0	0.000	0	0.000	0.0000
	Other specified rheumatoid arthritis. unspecified shoulder	M06.819	0	0.000	0	0.000	0	0.000	0	0.000	0.0000
	Other specified rheumatoid arthritis. elbow	M06.82	0	0.000	0	0.000	0	0.000	0	0.000	0.0000
	Other specified rheumatoid arthritis. right elbow	M06.821	0	0.000	0	0.000	0	0.000	0	0.000	0.0000
	Other specified rheumatoid arthritis. left elbow	M06.822	0	0.000	0	0.000	0	0.000	0	0.000	0.0000
	Other specified rheumatoid arthritis. unspecified elbow	M06.829	0	0.000	0	0.000	0	0.000	0	0.000	0.0000
	Other specified rheumatoid arthritis. wrist	M06.83	0	0.000	0	0.000	0	0.000	0	0.000	0.0000
	Other specified rheumatoid arthritis. right wrist	M06.831	0	0.000	0	0.000	0	0.000	0	0.000	0.0000
	Other specified rheumatoid arthritis. left wrist	M06.832	0	0.000	0	0.000	0	0.000	0	0.000	0.0000
	Other specified rheumatoid arthritis. unspecified wrist	M06.839	0	0.000	0	0.000	0	0.000	0	0.000	0.0000
	Other specified rheumatoid arthritis. hand	M06.84	0	0.000	0	0.000	0	0.000	0	0.000	0.0000
	Other specified rheumatoid arthritis. right hand	M06.841	0	0.000	0	0.000	0	0.000	0	0.000	0.0000
	Other specified rheumatoid arthritis. left hand	M06.842	0	0.000	0	0.000	0	0.000	0	0.000	0.0000
	Other specified rheumatoid arthritis. unspecified hand	M06.849	0	0.000	0	0.000	0	0.000	0	0.000	0.0000
	Other specified rheumatoid arthritis. hip	M06.85	0	0.000	0	0.000	0	0.000	0	0.000	0.0000
	Other specified rheumatoid arthritis. right hip	M06.851	0	0.000	0	0.000	0	0.000	0	0.000	0.0000
	Other specified rheumatoid arthritis. left hip	M06.852	0	0.000	0	0.000	0	0.000	0	0.000	0.0000
	Other specified rheumatoid arthritis. unspecified hip	M06.859	0	0.000	0	0.000	0	0.000	0	0.000	0.0000
	Other specified rheumatoid arthritis. knee	M06.86	0	0.000	0	0.000	0	0.000	0	0.000	0.0000
	Other specified rheumatoid arthritis. right knee	M06.861	0	0.000	0	0.000	0	0.000	0	0.000	0.0000
	Other specified rheumatoid arthritis. left knee	M06.862	0	0.000	0	0.000	0	0.000	0	0.000	0.0000
	Other specified rheumatoid arthritis. unspecified knee	M06.869	0	0.000	0	0.000	0	0.000	0	0.000	0.0000
	Other specified rheumatoid arthritis. ankle and foot	M06.87	0	0.000	0	0.000	0	0.000	0	0.000	0.0000
	Other specified rheumatoid arthritis. right ankle and foot	M06.871	0	0.000	0	0.000	0	0.000	0	0.000	0.0000
	Other specified rheumatoid arthritis. left ankle and foot	M06.872	0	0.000	0	0.000	0	0.000	0	0.000	0.0000
	Other specified rheumatoid arthritis. unspecified ankle and foot	M06.879	0	0.000	0	0.000	0	0.000	0	0.000	0.0000
	Other specified rheumatoid arthritis. vertebrae	M06.88	0	0.000	0	0.000	0	0.000	0	0.000	0.0000

	Other specified rheumatoid arthritis. multiple sites	M06.89	0	0.000	0	0.000	0	0.000	0	0.000	0.0000
	Other specified rheumatoid arthritis. other specified site	M06.8A	0	0.000	0	0.000	0	0.000	0	0.000	0.0000
	Rheumatoid arthritis. unspecified	M06.9	1010	6.343	493	4.207	128	4.755	1631	5.377	0.0000
	Distal interphalangeal psoriatic arthropathy	**M07.0**	0	0.000	1	0.009	1	0.037	2	0.007	0.8776
	Other psoriatic arthropathies	**M07.3**	98	0.615	54	0.461	22	0.817	174	0.574	0.1020
	Arthropathy in Crohn disease [regional enteritis]	**M07.4**	270	1.696	18	0.154	4	0.149	292	0.963	0.0000
	Arthropathy in ulcerative colitis	**M07.5**	9	0.057	72	0.614	3	0.111	84	0.277	0.0000
	**Enteropathic arthropathies**	**M07**	0	0.000	0	0.000	0	0.000	0	0.000	0.0000
	Enteropathic arthropathies	M07.6	371	2.330	343	2.927	74	2.749	788	2.598	0.0022
	Enteropathic arthropathies. unspecified site	M07.60	0	0.000	0	0.000	0	0.000	0	0.000	0.0000
	Enteropathic arthropathies. shoulder	M07.61	0	0.000	0	0.000	0	0.000	0	0.000	0.0000
	Enteropathic arthropathies. right shoulder	M07.611	0	0.000	0	0.000	0	0.000	0	0.000	0.0000
	Enteropathic arthropathies. left shoulder	M07.612	0	0.000	0	0.000	0	0.000	0	0.000	0.0000
	Enteropathic arthropathies. unspecified shoulder	M07.619	0	0.000	0	0.000	0	0.000	0	0.000	0.0000
	Enteropathic arthropathies. elbow	M07.62	0	0.000	0	0.000	0	0.000	0	0.000	0.0000
	Enteropathic arthropathies. right elbow	M07.621	0	0.000	0	0.000	0	0.000	0	0.000	0.0000
	Enteropathic arthropathies. left elbow	M07.622	0	0.000	0	0.000	0	0.000	0	0.000	0.0000
	Enteropathic arthropathies. unspecified elbow	M07.629	0	0.000	0	0.000	0	0.000	0	0.000	0.0000
	Enteropathic arthropathies. wrist	M07.63	0	0.000	0	0.000	0	0.000	0	0.000	0.0000
	Enteropathic arthropathies. right wrist	M07.631	0	0.000	0	0.000	0	0.000	0	0.000	0.0000
	Enteropathic arthropathies. left wrist	M07.632	0	0.000	0	0.000	0	0.000	0	0.000	0.0000
	Enteropathic arthropathies. unspecified wrist	M07.639	0	0.000	0	0.000	0	0.000	0	0.000	0.0000
	Enteropathic arthropathies. hand	M07.64	0	0.000	0	0.000	0	0.000	0	0.000	0.0000
	Enteropathic arthropathies. right hand	M07.641	0	0.000	0	0.000	0	0.000	0	0.000	0.0000
	Enteropathic arthropathies. left hand	M07.642	0	0.000	0	0.000	0	0.000	0	0.000	0.0000
	Enteropathic arthropathies. unspecified hand	M07.649	0	0.000	0	0.000	0	0.000	0	0.000	0.0000
	Enteropathic arthropathies. hip	M07.65	0	0.000	0	0.000	0	0.000	0	0.000	0.0000
	Enteropathic arthropathies. right hip	M07.651	0	0.000	0	0.000	0	0.000	0	0.000	0.0000
	Enteropathic arthropathies. left hip	M07.652	0	0.000	0	0.000	0	0.000	0	0.000	0.0000

	Enteropathic arthropathies. unspecified hip	M07.659	0	0.000	0	0.000	0	0.000	0	0.000	0.0000
	Enteropathic arthropathies. knee	M07.66	0	0.000	0	0.000	0	0.000	0	0.000	0.0000
	Enteropathic arthropathies. right knee	M07.661	0	0.000	0	0.000	0	0.000	0	0.000	0.0000
	Enteropathic arthropathies. left knee	M07.662	0	0.000	0	0.000	0	0.000	0	0.000	0.0000
	Enteropathic arthropathies. unspecified knee	M07.669	0	0.000	0	0.000	0	0.000	0	0.000	0.0000
	Enteropathic arthropathies. ankle and foot	M07.67	0	0.000	0	0.000	0	0.000	0	0.000	0.0000
	Enteropathic arthropathies. right ankle and foot	M07.671	0	0.000	0	0.000	0	0.000	0	0.000	0.0000
	Enteropathic arthropathies. left ankle and foot	M07.672	0	0.000	0	0.000	0	0.000	0	0.000	0.0000
	Enteropathic arthropathies. unspecified ankle and foot	M07.679	0	0.000	0	0.000	0	0.000	0	0.000	0.0000
	Enteropathic arthropathies. vertebrae	M07.68	0	0.000	0	0.000	0	0.000	0	0.000	0.0000
	Enteropathic arthropathies. multiple sites	M07.69	0	0.000	0	0.000	0	0.000	0	0.000	0.0000
	**Juvenile arthritis**	**M08**	0	0.000	0	0.000	0	0.000	0	0.000	0.0000
	Unspecified juvenile rheumatoid arthritis	M08.0	123	0.772	50	0.427	5	0.186	178	0.587	0.0004
	Unspecified juvenile rheumatoid arthritis of unspecified site	M08.00	0	0.000	0	0.000	0	0.000	0	0.000	0.0000
	Unspecified juvenile rheumatoid arthritis. shoulder	M08.01	0	0.000	0	0.000	0	0.000	0	0.000	0.0000
	Unspecified juvenile rheumatoid arthritis. right shoulder	M08.011	0	0.000	0	0.000	0	0.000	0	0.000	0.0000
	Unspecified juvenile rheumatoid arthritis. left shoulder	M08.012	0	0.000	0	0.000	0	0.000	0	0.000	0.0000
	Unspecified juvenile rheumatoid arthritis. unspecified shoulder	M08.019	0	0.000	0	0.000	0	0.000	0	0.000	0.0000
	Unspecified juvenile rheumatoid arthritis of elbow	M08.02	0	0.000	0	0.000	0	0.000	0	0.000	0.0000
	Unspecified juvenile rheumatoid arthritis. right elbow	M08.021	0	0.000	0	0.000	0	0.000	0	0.000	0.0000
	Unspecified juvenile rheumatoid arthritis. left elbow	M08.022	0	0.000	0	0.000	0	0.000	0	0.000	0.0000
	Unspecified juvenile rheumatoid arthritis. unspecified elbow	M08.029	0	0.000	0	0.000	0	0.000	0	0.000	0.0000
	Unspecified juvenile rheumatoid arthritis. wrist	M08.03	0	0.000	0	0.000	0	0.000	0	0.000	0.0000
	Unspecified juvenile rheumatoid arthritis. right wrist	M08.031	0	0.000	0	0.000	0	0.000	0	0.000	0.0000
	Unspecified juvenile rheumatoid arthritis. left wrist	M08.032	0	0.000	0	0.000	0	0.000	0	0.000	0.0000
	Unspecified juvenile rheumatoid arthritis. unspecified wrist	M08.039	0	0.000	0	0.000	0	0.000	0	0.000	0.0000
	Unspecified juvenile rheumatoid arthritis. hand	M08.04	0	0.000	0	0.000	0	0.000	0	0.000	0.0000
	Unspecified juvenile rheumatoid arthritis. right hand	M08.041	0	0.000	0	0.000	0	0.000	0	0.000	0.0000
	Unspecified juvenile rheumatoid arthritis. left hand	M08.042	0	0.000	0	0.000	0	0.000	0	0.000	0.0000

	Unspecified juvenile rheumatoid arthritis. unspecified hand	M08.049	0	0.000	0	0.000	0	0.000	0	0.000	0.0000
	Unspecified juvenile rheumatoid arthritis. hip	M08.05	0	0.000	0	0.000	0	0.000	0	0.000	0.0000
	Unspecified juvenile rheumatoid arthritis. right hip	M08.051	0	0.000	0	0.000	0	0.000	0	0.000	0.0000
	Unspecified juvenile rheumatoid arthritis. left hip	M08.052	0	0.000	0	0.000	0	0.000	0	0.000	0.0000
	Unspecified juvenile rheumatoid arthritis. unspecified hip	M08.059	0	0.000	0	0.000	0	0.000	0	0.000	0.0000
	Unspecified juvenile rheumatoid arthritis. knee	M08.06	0	0.000	0	0.000	0	0.000	0	0.000	0.0000
	Unspecified juvenile rheumatoid arthritis. right knee	M08.061	0	0.000	0	0.000	0	0.000	0	0.000	0.0000
	Unspecified juvenile rheumatoid arthritis. left knee	M08.062	0	0.000	0	0.000	0	0.000	0	0.000	0.0000
	Unspecified juvenile rheumatoid arthritis. unspecified knee	M08.069	0	0.000	0	0.000	0	0.000	0	0.000	0.0000
	Unspecified juvenile rheumatoid arthritis. ankle and foot	M08.07	0	0.000	0	0.000	0	0.000	0	0.000	0.0000
	Unspecified juvenile rheumatoid arthritis. right ankle and foot	M08.071	0	0.000	0	0.000	0	0.000	0	0.000	0.0000
	Unspecified juvenile rheumatoid arthritis. left ankle and foot	M08.072	0	0.000	0	0.000	0	0.000	0	0.000	0.0000
	Unspecified juvenile rheumatoid arthritis. unspecified ankle and foot	M08.079	0	0.000	0	0.000	0	0.000	0	0.000	0.0000
	Unspecified juvenile rheumatoid arthritis. vertebrae	M08.08	0	0.000	0	0.000	0	0.000	0	0.000	0.0000
	Unspecified juvenile rheumatoid arthritis. multiple sites	M08.09	0	0.000	0	0.000	0	0.000	0	0.000	0.0000
	Unspecified juvenile rheumatoid arthritis. other specified site	M08.0A	0	0.000	0	0.000	0	0.000	0	0.000	0.0000
	Juvenile ankylosing spondylitis	M08.1	5	0.031	2	0.017	1	0.037	8	0.026	0.7207
	Juvenile rheumatoid arthritis with systemic onset	M08.2	7	0.044	1	0.009	0	0.000	8	0.026	0.1759
	Juvenile rheumatoid arthritis with systemic onset. unspecified site	M08.20	0	0.000	0	0.000	0	0.000	0	0.000	0.0000
	Juvenile rheumatoid arthritis with systemic onset. shoulder	M08.21	0	0.000	0	0.000	0	0.000	0	0.000	0.0000
	Juvenile rheumatoid arthritis with systemic onset. right shoulder	M08.211	0	0.000	0	0.000	0	0.000	0	0.000	0.0000
	Juvenile rheumatoid arthritis with systemic onset. left shoulder	M08.212	0	0.000	0	0.000	0	0.000	0	0.000	0.0000
	Juvenile rheumatoid arthritis with systemic onset. unspecified shoulder	M08.219	0	0.000	0	0.000	0	0.000	0	0.000	0.0000
	Juvenile rheumatoid arthritis with systemic onset. elbow	M08.22	0	0.000	0	0.000	0	0.000	0	0.000	0.0000
	Juvenile rheumatoid arthritis with systemic onset. right elbow	M08.221	0	0.000	0	0.000	0	0.000	0	0.000	0.0000
	Juvenile rheumatoid arthritis with systemic onset. left elbow	M08.222	0	0.000	0	0.000	0	0.000	0	0.000	0.0000
	Juvenile rheumatoid arthritis with systemic onset. unspecified elbow	M08.229	0	0.000	0	0.000	0	0.000	0	0.000	0.0000
	Juvenile rheumatoid arthritis with systemic onset. wrist	M08.23	0	0.000	0	0.000	0	0.000	0	0.000	0.0000

	Juvenile rheumatoid arthritis with systemic onset. right wrist	M08.231	0	0.000	0	0.000	0	0.000	0	0.000	0.0000
	Juvenile rheumatoid arthritis with systemic onset. left wrist	M08.232	0	0.000	0	0.000	0	0.000	0	0.000	0.0000
	Juvenile rheumatoid arthritis with systemic onset. unspecified wrist	M08.239	0	0.000	0	0.000	0	0.000	0	0.000	0.0000
	Juvenile rheumatoid arthritis with systemic onset. hand	M08.24	0	0.000	0	0.000	0	0.000	0	0.000	0.0000
	Juvenile rheumatoid arthritis with systemic onset. right hand	M08.241	0	0.000	0	0.000	0	0.000	0	0.000	0.0000
	Juvenile rheumatoid arthritis with systemic onset. left hand	M08.242	0	0.000	0	0.000	0	0.000	0	0.000	0.0000
	Juvenile rheumatoid arthritis with systemic onset. unspecified hand	M08.249	0	0.000	0	0.000	0	0.000	0	0.000	0.0000
	Juvenile rheumatoid arthritis with systemic onset. hip	M08.25	0	0.000	0	0.000	0	0.000	0	0.000	0.0000
	Juvenile rheumatoid arthritis with systemic onset. right hip	M08.251	0	0.000	0	0.000	0	0.000	0	0.000	0.0000
	Juvenile rheumatoid arthritis with systemic onset. left hip	M08.252	0	0.000	0	0.000	0	0.000	0	0.000	0.0000
	Juvenile rheumatoid arthritis with systemic onset. unspecified hip	M08.259	0	0.000	0	0.000	0	0.000	0	0.000	0.0000
	Juvenile rheumatoid arthritis with systemic onset. knee	M08.26	0	0.000	0	0.000	0	0.000	0	0.000	0.0000
	Juvenile rheumatoid arthritis with systemic onset. right knee	M08.261	0	0.000	0	0.000	0	0.000	0	0.000	0.0000
	Juvenile rheumatoid arthritis with systemic onset. left knee	M08.262	0	0.000	0	0.000	0	0.000	0	0.000	0.0000
	Juvenile rheumatoid arthritis with systemic onset. unspecified knee	M08.269	0	0.000	0	0.000	0	0.000	0	0.000	0.0000
	Juvenile rheumatoid arthritis with systemic onset. ankle and foot	M08.27	0	0.000	0	0.000	0	0.000	0	0.000	0.0000
	Juvenile rheumatoid arthritis with systemic onset. right ankle and foot	M08.271	0	0.000	0	0.000	0	0.000	0	0.000	0.0000
	Juvenile rheumatoid arthritis with systemic onset. left ankle and foot	M08.272	0	0.000	0	0.000	0	0.000	0	0.000	0.0000
	Juvenile rheumatoid arthritis with systemic onset. unspecified ankle and foot	M08.279	0	0.000	0	0.000	0	0.000	0	0.000	0.0000
	Juvenile rheumatoid arthritis with systemic onset. vertebrae	M08.28	0	0.000	0	0.000	0	0.000	0	0.000	0.0000
	Juvenile rheumatoid arthritis with systemic onset. multiple sites	M08.29	0	0.000	0	0.000	0	0.000	0	0.000	0.0000
	Juvenile rheumatoid arthritis with systemic onset. other specified site	M08.2A	0	0.000	0	0.000	0	0.000	0	0.000	0.0000
	Juvenile rheumatoid polyarthritis	M08.3	10	0.063	2	0.017	0	0.000	12	0.040	0.1306
	Pauciarticular juvenile rheumatoid arthritis	M08.4	9	0.057	1	0.009	0	0.000	10	0.033	0.0796
	Pauciarticular juvenile rheumatoid arthritis. unspecified site	M08.40	0	0.000	0	0.000	0	0.000	0	0.000	0.0000
	Pauciarticular juvenile rheumatoid arthritis. shoulder	M08.41	0	0.000	0	0.000	0	0.000	0	0.000	0.0000
	Pauciarticular juvenile rheumatoid arthritis. right shoulder	M08.411	0	0.000	0	0.000	0	0.000	0	0.000	0.0000

	Pauciarticular juvenile rheumatoid arthritis. left shoulder	M08.412	0	0.000	0	0.000	0	0.000	0	0.000	0.0000
	Pauciarticular juvenile rheumatoid arthritis. unspecified shoulder	M08.419	0	0.000	0	0.000	0	0.000	0	0.000	0.0000
	Pauciarticular juvenile rheumatoid arthritis. elbow	M08.42	0	0.000	0	0.000	0	0.000	0	0.000	0.0000
	Pauciarticular juvenile rheumatoid arthritis. right elbow	M08.421	0	0.000	0	0.000	0	0.000	0	0.000	0.0000
	Pauciarticular juvenile rheumatoid arthritis. left elbow	M08.422	0	0.000	0	0.000	0	0.000	0	0.000	0.0000
	Pauciarticular juvenile rheumatoid arthritis. unspecified elbow	M08.429	0	0.000	0	0.000	0	0.000	0	0.000	0.0000
	Pauciarticular juvenile rheumatoid arthritis. wrist	M08.43	0	0.000	0	0.000	0	0.000	0	0.000	0.0000
	Pauciarticular juvenile rheumatoid arthritis. right wrist	M08.431	0	0.000	0	0.000	0	0.000	0	0.000	0.0000
	Pauciarticular juvenile rheumatoid arthritis. left wrist	M08.432	0	0.000	0	0.000	0	0.000	0	0.000	0.0000
	Pauciarticular juvenile rheumatoid arthritis. unspecified wrist	M08.439	0	0.000	0	0.000	0	0.000	0	0.000	0.0000
	Pauciarticular juvenile rheumatoid arthritis. hand	M08.44	0	0.000	0	0.000	0	0.000	0	0.000	0.0000
	Pauciarticular juvenile rheumatoid arthritis. right hand	M08.441	0	0.000	0	0.000	0	0.000	0	0.000	0.0000
	Pauciarticular juvenile rheumatoid arthritis. left hand	M08.442	0	0.000	0	0.000	0	0.000	0	0.000	0.0000
	Pauciarticular juvenile rheumatoid arthritis. unspecified hand	M08.449	0	0.000	0	0.000	0	0.000	0	0.000	0.0000
	Pauciarticular juvenile rheumatoid arthritis. hip	M08.45	0	0.000	0	0.000	0	0.000	0	0.000	0.0000
	Pauciarticular juvenile rheumatoid arthritis. right hip	M08.451	0	0.000	0	0.000	0	0.000	0	0.000	0.0000
	Pauciarticular juvenile rheumatoid arthritis. left hip	M08.452	0	0.000	0	0.000	0	0.000	0	0.000	0.0000
	Pauciarticular juvenile rheumatoid arthritis. unspecified hip	M08.459	0	0.000	0	0.000	0	0.000	0	0.000	0.0000
	Pauciarticular juvenile rheumatoid arthritis. knee	M08.46	0	0.000	0	0.000	0	0.000	0	0.000	0.0000
	Pauciarticular juvenile rheumatoid arthritis. right knee	M08.461	0	0.000	0	0.000	0	0.000	0	0.000	0.0000
	Pauciarticular juvenile rheumatoid arthritis. left knee	M08.462	0	0.000	0	0.000	0	0.000	0	0.000	0.0000
	Pauciarticular juvenile rheumatoid arthritis. unspecified knee	M08.469	0	0.000	0	0.000	0	0.000	0	0.000	0.0000
	Pauciarticular juvenile rheumatoid arthritis. ankle and foot	M08.47	0	0.000	0	0.000	0	0.000	0	0.000	0.0000
	Pauciarticular juvenile rheumatoid arthritis. right ankle and foot	M08.471	0	0.000	0	0.000	0	0.000	0	0.000	0.0000
	Pauciarticular juvenile rheumatoid arthritis. left ankle and foot	M08.472	0	0.000	0	0.000	0	0.000	0	0.000	0.0000
	Pauciarticular juvenile rheumatoid arthritis. unspecified ankle and foot	M08.479	0	0.000	0	0.000	0	0.000	0	0.000	0.0000
	Pauciarticular juvenile rheumatoid arthritis. vertebrae	M08.48	0	0.000	0	0.000	0	0.000	0	0.000	0.0000
	Pauciarticular juvenile rheumatoid arthritis. other specified site	M08.4A	0	0.000	0	0.000	0	0.000	0	0.000	0.0000

	Other juvenile arthritis	M08.8	15	0.094	4	0.034	0	0.000	19	0.063	0.0988
	Other juvenile arthritis. unspecified site	M08.80	0	0.000	0	0.000	0	0.000	0	0.000	0.0000
	Other juvenile arthritis. shoulder	M08.81	0	0.000	0	0.000	0	0.000	0	0.000	0.0000
	Other juvenile arthritis. right shoulder	M08.811	0	0.000	0	0.000	0	0.000	0	0.000	0.0000
	Other juvenile arthritis. left shoulder	M08.812	0	0.000	0	0.000	0	0.000	0	0.000	0.0000
	Other juvenile arthritis. unspecified shoulder	M08.819	0	0.000	0	0.000	0	0.000	0	0.000	0.0000
	Other juvenile arthritis. elbow	M08.82	0	0.000	0	0.000	0	0.000	0	0.000	0.0000
	Other juvenile arthritis. right elbow	M08.821	0	0.000	0	0.000	0	0.000	0	0.000	0.0000
	Other juvenile arthritis. left elbow	M08.822	0	0.000	0	0.000	0	0.000	0	0.000	0.0000
	Other juvenile arthritis. unspecified elbow	M08.829	0	0.000	0	0.000	0	0.000	0	0.000	0.0000
	Other juvenile arthritis. wrist	M08.83	0	0.000	0	0.000	0	0.000	0	0.000	0.0000
	Other juvenile arthritis. right wrist	M08.831	0	0.000	0	0.000	0	0.000	0	0.000	0.0000
	Other juvenile arthritis. left wrist	M08.832	0	0.000	0	0.000	0	0.000	0	0.000	0.0000
	Other juvenile arthritis. unspecified wrist	M08.839	0	0.000	0	0.000	0	0.000	0	0.000	0.0000
	Other juvenile arthritis. hand	M08.84	0	0.000	0	0.000	0	0.000	0	0.000	0.0000
	Other juvenile arthritis. right hand	M08.841	0	0.000	0	0.000	0	0.000	0	0.000	0.0000
	Other juvenile arthritis. left hand	M08.842	0	0.000	0	0.000	0	0.000	0	0.000	0.0000
	Other juvenile arthritis. unspecified hand	M08.849	0	0.000	0	0.000	0	0.000	0	0.000	0.0000
	Other juvenile arthritis. hip	M08.85	0	0.000	0	0.000	0	0.000	0	0.000	0.0000
	Other juvenile arthritis. right hip	M08.851	0	0.000	0	0.000	0	0.000	0	0.000	0.0000
	Other juvenile arthritis. left hip	M08.852	0	0.000	0	0.000	0	0.000	0	0.000	0.0000
	Other juvenile arthritis. unspecified hip	M08.859	0	0.000	0	0.000	0	0.000	0	0.000	0.0000
	Other juvenile arthritis. knee	M08.86	0	0.000	0	0.000	0	0.000	0	0.000	0.0000
	Other juvenile arthritis. right knee	M08.861	0	0.000	0	0.000	0	0.000	0	0.000	0.0000
	Other juvenile arthritis. left knee	M08.862	0	0.000	0	0.000	0	0.000	0	0.000	0.0000
	Other juvenile arthritis. unspecified knee	M08.869	0	0.000	0	0.000	0	0.000	0	0.000	0.0000
	Other juvenile arthritis. ankle and foot	M08.87	0	0.000	0	0.000	0	0.000	0	0.000	0.0000
	Other juvenile arthritis. right ankle and foot	M08.871	0	0.000	0	0.000	0	0.000	0	0.000	0.0000
	Other juvenile arthritis. left ankle and foot	M08.872	0	0.000	0	0.000	0	0.000	0	0.000	0.0000

	Other juvenile arthritis. unspecified ankle and foot	M08.879	0	0.000	0	0.000	0	0.000	0	0.000	0.0000
	Other juvenile arthritis. other specified site	M08.88	0	0.000	0	0.000	0	0.000	0	0.000	0.0000
	Other juvenile arthritis. multiple sites	M08.89	0	0.000	0	0.000	0	0.000	0	0.000	0.0000
	Juvenile arthritis. unspecified	M08.9	49	0.308	16	0.137	2	0.074	67	0.221	0.0055
	Juvenile arthritis. unspecified. unspecified site	M08.90	0	0.000	0	0.000	0	0.000	0	0.000	0.0000
	Juvenile arthritis. unspecified. shoulder	M08.91	0	0.000	0	0.000	0	0.000	0	0.000	0.0000
	Juvenile arthritis. unspecified. right shoulder	M08.911	0	0.000	0	0.000	0	0.000	0	0.000	0.0000
	Juvenile arthritis. unspecified. left shoulder	M08.912	0	0.000	0	0.000	0	0.000	0	0.000	0.0000
	Juvenile arthritis. unspecified. unspecified shoulder	M08.919	0	0.000	0	0.000	0	0.000	0	0.000	0.0000
	Juvenile arthritis. unspecified. elbow	M08.92	0	0.000	0	0.000	0	0.000	0	0.000	0.0000
	Juvenile arthritis. unspecified. right elbow	M08.921	0	0.000	0	0.000	0	0.000	0	0.000	0.0000
	Juvenile arthritis. unspecified. left elbow	M08.922	0	0.000	0	0.000	0	0.000	0	0.000	0.0000
	Juvenile arthritis. unspecified. unspecified elbow	M08.929	0	0.000	0	0.000	0	0.000	0	0.000	0.0000
	Juvenile arthritis. unspecified. wrist	M08.93	0	0.000	0	0.000	0	0.000	0	0.000	0.0000
	Juvenile arthritis. unspecified. right wrist	M08.931	0	0.000	0	0.000	0	0.000	0	0.000	0.0000
	Juvenile arthritis. unspecified. left wrist	M08.932	0	0.000	0	0.000	0	0.000	0	0.000	0.0000
	Juvenile arthritis. unspecified. unspecified wrist	M08.939	0	0.000	0	0.000	0	0.000	0	0.000	0.0000
	Juvenile arthritis. unspecified. hand	M08.94	0	0.000	0	0.000	0	0.000	0	0.000	0.0000
	Juvenile arthritis. unspecified. right hand	M08.941	0	0.000	0	0.000	0	0.000	0	0.000	0.0000
	Juvenile arthritis. unspecified. left hand	M08.942	0	0.000	0	0.000	0	0.000	0	0.000	0.0000
	Juvenile arthritis. unspecified. unspecified hand	M08.949	0	0.000	0	0.000	0	0.000	0	0.000	0.0000
	Juvenile arthritis. unspecified. hip	M08.95	0	0.000	0	0.000	0	0.000	0	0.000	0.0000
	Juvenile arthritis. unspecified. right hip	M08.951	0	0.000	0	0.000	0	0.000	0	0.000	0.0000
	Juvenile arthritis. unspecified. left hip	M08.952	0	0.000	0	0.000	0	0.000	0	0.000	0.0000
	Juvenile arthritis. unspecified. unspecified hip	M08.959	0	0.000	0	0.000	0	0.000	0	0.000	0.0000
	Juvenile arthritis. unspecified. knee	M08.96	0	0.000	0	0.000	0	0.000	0	0.000	0.0000
	Juvenile arthritis. unspecified. right knee	M08.961	0	0.000	0	0.000	0	0.000	0	0.000	0.0000
	Juvenile arthritis. unspecified. left knee	M08.962	0	0.000	0	0.000	0	0.000	0	0.000	0.0000
	Juvenile arthritis. unspecified. unspecified knee	M08.969	0	0.000	0	0.000	0	0.000	0	0.000	0.0000

	Juvenile arthritis. unspecified. ankle and foot	M08.97	0	0.000	0	0.000	0	0.000	0	0.000	0.0000
	Juvenile arthritis. unspecified. right ankle and foot	M08.971	0	0.000	0	0.000	0	0.000	0	0.000	0.0000
	Juvenile arthritis. unspecified. left ankle and foot	M08.972	0	0.000	0	0.000	0	0.000	0	0.000	0.0000
	Juvenile arthritis. unspecified. unspecified ankle and foot	M08.979	0	0.000	0	0.000	0	0.000	0	0.000	0.0000
	Juvenile arthritis. unspecified. vertebrae	M08.98	0	0.000	0	0.000	0	0.000	0	0.000	0.0000
	Juvenile arthritis. unspecified. multiple sites	M08.99	0	0.000	0	0.000	0	0.000	0	0.000	0.0000
	Juvenile arthritis. unspecified. other specified site	M08.9A	0	0.000	0	0.000	0	0.000	0	0.000	0.0000
	Juvenile arthritis in Crohn disease [regional enteritis]	**M09.1**	21	0.132	4	0.034	0	0.000	25	0.082	0.0135
	Juvenile arthritis in ulcerative colitis	**M09.2**	2	0.013	6	0.051	0	0.000	8	0.026	0.1313
	**Other and unspecified arthropathy**	**M12**	0	0.000	0	0.000	0	0.000	0	0.000	0.0000
	Chronic postrheumatic arthropathy [Jaccoud]	M12.0	0	0.000	0	0.000	0	0.000	0	0.000	0.0000
	Chronic postrheumatic arthropathy [Jaccoud]. unspecified site	M12.00	0	0.000	0	0.000	0	0.000	0	0.000	0.0000
	Chronic postrheumatic arthropathy [Jaccoud]. shoulder	M12.01	0	0.000	0	0.000	0	0.000	0	0.000	0.0000
	Chronic postrheumatic arthropathy [Jaccoud]. right shoulder	M12.011	0	0.000	0	0.000	0	0.000	0	0.000	0.0000
	Chronic postrheumatic arthropathy [Jaccoud]. left shoulder	M12.012	0	0.000	0	0.000	0	0.000	0	0.000	0.0000
	Chronic postrheumatic arthropathy [Jaccoud]. unspecified shoulder	M12.019	0	0.000	0	0.000	0	0.000	0	0.000	0.0000
	Chronic postrheumatic arthropathy [Jaccoud]. elbow	M12.02	0	0.000	0	0.000	0	0.000	0	0.000	0.0000
	Chronic postrheumatic arthropathy [Jaccoud]. right elbow	M12.021	0	0.000	0	0.000	0	0.000	0	0.000	0.0000
	Chronic postrheumatic arthropathy [Jaccoud]. left elbow	M12.022	0	0.000	0	0.000	0	0.000	0	0.000	0.0000
	Chronic postrheumatic arthropathy [Jaccoud]. unspecified elbow	M12.029	0	0.000	0	0.000	0	0.000	0	0.000	0.0000
	Chronic postrheumatic arthropathy [Jaccoud]. wrist	M12.03	0	0.000	0	0.000	0	0.000	0	0.000	0.0000
	Chronic postrheumatic arthropathy [Jaccoud]. right wrist	M12.031	0	0.000	0	0.000	0	0.000	0	0.000	0.0000
	Chronic postrheumatic arthropathy [Jaccoud]. left wrist	M12.032	0	0.000	0	0.000	0	0.000	0	0.000	0.0000
	Chronic postrheumatic arthropathy [Jaccoud]. unspecified wrist	M12.039	0	0.000	0	0.000	0	0.000	0	0.000	0.0000
	Chronic postrheumatic arthropathy [Jaccoud]. hand	M12.04	0	0.000	0	0.000	0	0.000	0	0.000	0.0000
	Chronic postrheumatic arthropathy [Jaccoud]. right hand	M12.041	0	0.000	0	0.000	0	0.000	0	0.000	0.0000
	Chronic postrheumatic arthropathy [Jaccoud]. left hand	M12.042	0	0.000	0	0.000	0	0.000	0	0.000	0.0000
	Chronic postrheumatic arthropathy [Jaccoud]. unspecified hand	M12.049	0	0.000	0	0.000	0	0.000	0	0.000	0.0000

	Chronic postrheumatic arthropathy [Jaccoud]. hip	M12.05	0	0.000	0	0.000	0	0.000	0	0.000	0.0000
	Chronic postrheumatic arthropathy [Jaccoud]. right hip	M12.051	0	0.000	0	0.000	0	0.000	0	0.000	0.0000
	Chronic postrheumatic arthropathy [Jaccoud]. left hip	M12.052	0	0.000	0	0.000	0	0.000	0	0.000	0.0000
	Chronic postrheumatic arthropathy [Jaccoud]. unspecified hip	M12.059	0	0.000	0	0.000	0	0.000	0	0.000	0.0000
	Chronic postrheumatic arthropathy [Jaccoud]. knee	M12.06	0	0.000	0	0.000	0	0.000	0	0.000	0.0000
	Chronic postrheumatic arthropathy [Jaccoud]. right knee	M12.061	0	0.000	0	0.000	0	0.000	0	0.000	0.0000
	Chronic postrheumatic arthropathy [Jaccoud]. left knee	M12.062	0	0.000	0	0.000	0	0.000	0	0.000	0.0000
	Chronic postrheumatic arthropathy [Jaccoud]. unspecified knee	M12.069	0	0.000	0	0.000	0	0.000	0	0.000	0.0000
	Chronic postrheumatic arthropathy [Jaccoud]. ankle and foot	M12.07	0	0.000	0	0.000	0	0.000	0	0.000	0.0000
	Chronic postrheumatic arthropathy [Jaccoud]. right ankle and foot	M12.071	0	0.000	0	0.000	0	0.000	0	0.000	0.0000
	Chronic postrheumatic arthropathy [Jaccoud]. left ankle and foot	M12.072	0	0.000	0	0.000	0	0.000	0	0.000	0.0000
	Chronic postrheumatic arthropathy [Jaccoud]. unspecified ankle and foot	M12.079	0	0.000	0	0.000	0	0.000	0	0.000	0.0000
	Chronic postrheumatic arthropathy [Jaccoud]. other specified site	M12.08	0	0.000	0	0.000	0	0.000	0	0.000	0.0000
	Chronic postrheumatic arthropathy [Jaccoud]. multiple sites	M12.09	0	0.000	0	0.000	0	0.000	0	0.000	0.0000
	Villonodular synovitis	M12.2	0	0.000	0	0.000	0	0.000	0	0.000	0.0000
	Villonodular synovitis	M12.20	0	0.000	0	0.000	0	0.000	0	0.000	0.0000
	Villonodular synovitis	M12.21	0	0.000	0	0.000	0	0.000	0	0.000	0.0000
	Villonodular synovitis	M12.211	0	0.000	0	0.000	0	0.000	0	0.000	0.0000
	Villonodular synovitis	M12.212	0	0.000	0	0.000	0	0.000	0	0.000	0.0000
	Villonodular synovitis	M12.219	0	0.000	0	0.000	0	0.000	0	0.000	0.0000
	Villonodular synovitis	M12.22	0	0.000	0	0.000	0	0.000	0	0.000	0.0000
	Villonodular synovitis	M12.221	0	0.000	0	0.000	0	0.000	0	0.000	0.0000
	Villonodular synovitis	M12.222	0	0.000	0	0.000	0	0.000	0	0.000	0.0000
	Villonodular synovitis	M12.229	0	0.000	0	0.000	0	0.000	0	0.000	0.0000
	Villonodular synovitis	M12.23	0	0.000	0	0.000	0	0.000	0	0.000	0.0000
	Villonodular synovitis	M12.231	0	0.000	0	0.000	0	0.000	0	0.000	0.0000
	Villonodular synovitis	M12.232	0	0.000	0	0.000	0	0.000	0	0.000	0.0000
	Villonodular synovitis	M12.239	0	0.000	0	0.000	0	0.000	0	0.000	0.0000

	Villonodular synovitis	M12.24	0	0.000	0	0.000	0	0.000	0	0.000	0.0000
	Villonodular synovitis	M12.241	0	0.000	0	0.000	0	0.000	0	0.000	0.0000
	Villonodular synovitis	M12.242	0	0.000	0	0.000	0	0.000	0	0.000	0.0000
	Villonodular synovitis	M12.249	0	0.000	0	0.000	0	0.000	0	0.000	0.0000
	Villonodular synovitis	M12.25	0	0.000	0	0.000	0	0.000	0	0.000	0.0000
	Villonodular synovitis	M12.251	0	0.000	0	0.000	0	0.000	0	0.000	0.0000
	Villonodular synovitis	M12.252	0	0.000	0	0.000	0	0.000	0	0.000	0.0000
	Villonodular synovitis	M12.259	0	0.000	0	0.000	0	0.000	0	0.000	0.0000
	Villonodular synovitis	M12.26	0	0.000	2	0.017	0	0.000	2	0.007	0.3507
	Villonodular synovitis	M12.261	0	0.000	0	0.000	0	0.000	0	0.000	0.0000
	Villonodular synovitis	M12.262	0	0.000	0	0.000	0	0.000	0	0.000	0.0000
	Villonodular synovitis	M12.269	0	0.000	0	0.000	0	0.000	0	0.000	0.0000
	Villonodular synovitis	M12.27	1	0.006	0	0.000	0	0.000	1	0.003	0.8776
	Villonodular synovitis	M12.271	0	0.000	0	0.000	0	0.000	0	0.000	0.0000
	Villonodular synovitis	M12.272	0	0.000	0	0.000	0	0.000	0	0.000	0.0000
	Villonodular synovitis	M12.279	0	0.000	0	0.000	0	0.000	0	0.000	0.0000
	Villonodular synovitis	M12.28	1	0.006	0	0.000	0	0.000	1	0.003	0.8776
	Villonodular synovitis	M12.29	0	0.000	0	0.000	0	0.000	0	0.000	0.0000
	Palindromic rheumatism	M12.3	0	0.000	0	0.000	0	0.000	0	0.000	0.0000
	Palindromic rheumatism. unspecified site	M12.30	0	0.000	3	0.026	1	0.037	4	0.013	0.1513
	Palindromic rheumatism. shoulder	M12.31	0	0.000	0	0.000	0	0.000	0	0.000	0.0000
	Palindromic rheumatism. right shoulder	M12.311	0	0.000	0	0.000	0	0.000	0	0.000	0.0000
	Palindromic rheumatism. left shoulder	M12.312	0	0.000	0	0.000	0	0.000	0	0.000	0.0000
	Palindromic rheumatism. unspecified shoulder	M12.319	0	0.000	0	0.000	0	0.000	0	0.000	0.0000
	Palindromic rheumatism. elbow	M12.32	0	0.000	0	0.000	0	0.000	0	0.000	0.0000
	Palindromic rheumatism. right elbow	M12.321	0	0.000	0	0.000	0	0.000	0	0.000	0.0000
	Palindromic rheumatism. left elbow	M12.322	0	0.000	0	0.000	0	0.000	0	0.000	0.0000
	Palindromic rheumatism. unspecified elbow	M12.329	0	0.000	0	0.000	0	0.000	0	0.000	0.0000
	Palindromic rheumatism. wrist	M12.33	0	0.000	2	0.017	0	0.000	2	0.007	0.3507

	Palindromic rheumatism. right wrist	M12.331	0	0.000	0	0.000	0	0.000	0	0.000	0.0000
	Palindromic rheumatism. left wrist	M12.332	0	0.000	0	0.000	0	0.000	0	0.000	0.0000
	Palindromic rheumatism. unspecified wrist	M12.339	0	0.000	0	0.000	0	0.000	0	0.000	0.0000
	Palindromic rheumatism. hand	M12.34	0	0.000	1	0.009	0	0.000	1	0.003	0.8776
	Palindromic rheumatism. right hand	M12.341	0	0.000	0	0.000	0	0.000	0	0.000	0.0000
	Palindromic rheumatism. left hand	M12.342	0	0.000	0	0.000	0	0.000	0	0.000	0.0000
	Palindromic rheumatism. unspecified hand	M12.349	0	0.000	0	0.000	0	0.000	0	0.000	0.0000
	Palindromic rheumatism. hip	M12.35	0	0.000	0	0.000	0	0.000	0	0.000	0.0000
	Palindromic rheumatism. right hip	M12.351	0	0.000	0	0.000	0	0.000	0	0.000	0.0000
	Palindromic rheumatism. left hip	M12.352	0	0.000	0	0.000	0	0.000	0	0.000	0.0000
	Palindromic rheumatism. unspecified hip	M12.359	0	0.000	0	0.000	0	0.000	0	0.000	0.0000
	Palindromic rheumatism. knee	M12.36	0	0.000	0	0.000	0	0.000	0	0.000	0.0000
	Palindromic rheumatism. right knee	M12.361	0	0.000	0	0.000	0	0.000	0	0.000	0.0000
	Palindromic rheumatism. left knee	M12.362	0	0.000	0	0.000	0	0.000	0	0.000	0.0000
	Palindromic rheumatism. unspecified knee	M12.369	0	0.000	0	0.000	0	0.000	0	0.000	0.0000
	Palindromic rheumatism. ankle and foot	M12.37	0	0.000	0	0.000	0	0.000	0	0.000	0.0000
	Palindromic rheumatism. right ankle and foot	M12.371	0	0.000	0	0.000	0	0.000	0	0.000	0.0000
	Palindromic rheumatism. left ankle and foot	M12.372	0	0.000	0	0.000	0	0.000	0	0.000	0.0000
	Palindromic rheumatism. unspecified ankle and foot	M12.379	0	0.000	0	0.000	0	0.000	0	0.000	0.0000
	Palindromic rheumatism. other specified site	M12.38	1	0.006	0	0.000	0	0.000	1	0.003	0.8776
	Palindromic rheumatism. multiple sites	M12.39	7	0.044	7	0.060	1	0.037	15	0.049	0.7598
	Intermittent hydrarthrosis	M12.4	0	0.000	0	0.000	0	0.000	0	0.000	0.0000
	Intermittent hydrarthrosis. unspecified site	M12.40	0	0.000	0	0.000	0	0.000	0	0.000	0.0000
	Intermittent hydrarthrosis. shoulder	M12.41	0	0.000	0	0.000	0	0.000	0	0.000	0.0000
	Intermittent hydrarthrosis. right shoulder	M12.411	0	0.000	0	0.000	0	0.000	0	0.000	0.0000
	Intermittent hydrarthrosis. left shoulder	M12.412	0	0.000	0	0.000	0	0.000	0	0.000	0.0000
	Intermittent hydrarthrosis. unspecified shoulder	M12.419	0	0.000	0	0.000	0	0.000	0	0.000	0.0000
	Intermittent hydrarthrosis. elbow	M12.42	0	0.000	0	0.000	0	0.000	0	0.000	0.0000
	Intermittent hydrarthrosis. right elbow	M12.421	0	0.000	0	0.000	0	0.000	0	0.000	0.0000

	Intermittent hydrarthrosis. left elbow	M12.422	0	0.000	0	0.000	0	0.000	0	0.000	0.0000
	Intermittent hydrarthrosis. unspecified elbow	M12.429	0	0.000	0	0.000	0	0.000	0	0.000	0.0000
	Intermittent hydrarthrosis. wrist	M12.43	0	0.000	0	0.000	0	0.000	0	0.000	0.0000
	Intermittent hydrarthrosis. right wrist	M12.431	0	0.000	0	0.000	0	0.000	0	0.000	0.0000
	Intermittent hydrarthrosis. left wrist	M12.432	0	0.000	0	0.000	0	0.000	0	0.000	0.0000
	Intermittent hydrarthrosis. unspecified wrist	M12.439	0	0.000	0	0.000	0	0.000	0	0.000	0.0000
	Intermittent hydrarthrosis. hand	M12.44	0	0.000	0	0.000	0	0.000	0	0.000	0.0000
	Intermittent hydrarthrosis. right hand	M12.441	0	0.000	0	0.000	0	0.000	0	0.000	0.0000
	Intermittent hydrarthrosis. left hand	M12.442	0	0.000	0	0.000	0	0.000	0	0.000	0.0000
	Intermittent hydrarthrosis. unspecified hand	M12.449	0	0.000	0	0.000	0	0.000	0	0.000	0.0000
	Intermittent hydrarthrosis. hip	M12.45	0	0.000	0	0.000	0	0.000	0	0.000	0.0000
	Intermittent hydrarthrosis. right hip	M12.451	0	0.000	0	0.000	0	0.000	0	0.000	0.0000
	Intermittent hydrarthrosis. left hip	M12.452	0	0.000	0	0.000	0	0.000	0	0.000	0.0000
	Intermittent hydrarthrosis. unspecified hip	M12.459	0	0.000	0	0.000	0	0.000	0	0.000	0.0000
	Intermittent hydrarthrosis. knee	M12.46	0	0.000	0	0.000	0	0.000	0	0.000	0.0000
	Intermittent hydrarthrosis. right knee	M12.461	0	0.000	0	0.000	0	0.000	0	0.000	0.0000
	Intermittent hydrarthrosis. left knee	M12.462	0	0.000	0	0.000	0	0.000	0	0.000	0.0000
	Intermittent hydrarthrosis. unspecified knee	M12.469	0	0.000	0	0.000	0	0.000	0	0.000	0.0000
	Intermittent hydrarthrosis. ankle and foot	M12.47	0	0.000	0	0.000	0	0.000	0	0.000	0.0000
	Intermittent hydrarthrosis. right ankle and foot	M12.471	0	0.000	0	0.000	0	0.000	0	0.000	0.0000
	Intermittent hydrarthrosis. left ankle and foot	M12.472	0	0.000	0	0.000	0	0.000	0	0.000	0.0000
	Intermittent hydrarthrosis. unspecified ankle and foot	M12.479	0	0.000	0	0.000	0	0.000	0	0.000	0.0000
	Intermittent hydrarthrosis. other site	M12.48	0	0.000	0	0.000	0	0.000	0	0.000	0.0000
	Intermittent hydrarthrosis. multiple sites	M12.49	0	0.000	0	0.000	0	0.000	0	0.000	0.0000
	Other specific arthropathies. not elsewhere classified	M12.8	0	0.000	0	0.000	0	0.000	0	0.000	0.0000
	Other specific arthropathies. not elsewhere classified. unspecified site	M12.80	5	0.031	3	0.026	0	0.000	8	0.026	0.9380
	Other specific arthropathies. not elsewhere classified. shoulder	M12.81	1	0.006	1	0.009	2	0.074	4	0.013	0.6187
	Other specific arthropathies. not elsewhere classified. right shoulder	M12.811	0	0.000	0	0.000	0	0.000	0	0.000	0.0000
	Other specific arthropathies. not elsewhere classified. left	M12.812	0	0.000	0	0.000	0	0.000	0	0.000	0.0000

	shoulder										
	Other specific arthropathies. not elsewhere classified. unspecified shoulder	M12.819	0	0.000	0	0.000	0	0.000	0	0.000	0.0000
	Other specific arthropathies. not elsewhere classified. elbow	M12.82	0	0.000	0	0.000	0	0.000	0	0.000	0.0000
	Other specific arthropathies. not elsewhere classified. right elbow	M12.821	0	0.000	0	0.000	0	0.000	0	0.000	0.0000
	Other specific arthropathies. not elsewhere classified. left elbow	M12.822	0	0.000	0	0.000	0	0.000	0	0.000	0.0000
	Other specific arthropathies. not elsewhere classified. unspecified elbow	M12.829	0	0.000	0	0.000	0	0.000	0	0.000	0.0000
	Other specific arthropathies. not elsewhere classified. wrist	M12.83	0	0.000	0	0.000	0	0.000	0	0.000	0.0000
	Other specific arthropathies. not elsewhere classified. right wrist	M12.831	0	0.000	0	0.000	0	0.000	0	0.000	0.0000
	Other specific arthropathies. not elsewhere classified. left wrist	M12.832	0	0.000	0	0.000	0	0.000	0	0.000	0.0000
	Other specific arthropathies. not elsewhere classified. unspecified wrist	M12.839	0	0.000	0	0.000	0	0.000	0	0.000	0.0000
	Other specific arthropathies. not elsewhere classified. hand	M12.84	0	0.000	0	0.000	0	0.000	0	0.000	0.0000
	Other specific arthropathies. not elsewhere classified. right hand	M12.841	0	0.000	0	0.000	0	0.000	0	0.000	0.0000
	Other specific arthropathies. not elsewhere classified. left hand	M12.842	0	0.000	0	0.000	0	0.000	0	0.000	0.0000
	Other specific arthropathies. not elsewhere classified. unspecified hand	M12.849	0	0.000	0	0.000	0	0.000	0	0.000	0.0000
	Other specific arthropathies. not elsewhere classified. hip	M12.85	0	0.000	1	0.009	1	0.037	2	0.007	0.8776
	Other specific arthropathies. not elsewhere classified. right hip	M12.851	0	0.000	0	0.000	0	0.000	0	0.000	0.0000
	Other specific arthropathies. not elsewhere classified. left hip	M12.852	0	0.000	0	0.000	0	0.000	0	0.000	0.0000
	Other specific arthropathies. not elsewhere classified. unspecified hip	M12.859	0	0.000	0	0.000	0	0.000	0	0.000	0.0000
	Other specific arthropathies. not elsewhere classified. knee	M12.86	3	0.019	0	0.000	1	0.037	4	0.013	0.3672
	Other specific arthropathies. not elsewhere classified. right knee	M12.861	0	0.000	0	0.000	0	0.000	0	0.000	0.0000
	Other specific arthropathies. not elsewhere classified. left knee	M12.862	0	0.000	0	0.000	0	0.000	0	0.000	0.0000
	Other specific arthropathies. not elsewhere classified. unspecified knee	M12.869	0	0.000	0	0.000	0	0.000	0	0.000	0.0000
	Other specific arthropathies. not elsewhere classified. ankle and foot	M12.87	2	0.013	5	0.043	0	0.000	7	0.023	0.2411
	Other specific arthropathies. not elsewhere classified. right ankle and foot	M12.871	0	0.000	0	0.000	0	0.000	0	0.000	0.0000
	Other specific arthropathies. not elsewhere classified. left ankle and foot	M12.872	0	0.000	0	0.000	0	0.000	0	0.000	0.0000

	Other specific arthropathies. not elsewhere classified. unspecified ankle and foot	M12.879	0	0.000	0	0.000	0	0.000	0	0.000	0.0000
	Other specific arthropathies. not elsewhere classified. other specified site	M12.88	2	0.013	1	0.009	0	0.000	3	0.010	0.7897
	Other specific arthropathies. not elsewhere classified. multiple sites	M12.89	9	0.057	7	0.060	0	0.000	16	0.053	0.8862
	Arthropathy. unspecified	M12.9	0	0.000	0	0.000	0	0.000	0	0.000	0.0000
	**Other arthritis**	**M13**	0	0.000	0	0.000	0	0.000	0	0.000	0.0000
	Polyarthritis. unspecified	M13.0	63	0.396	26	0.222	8	0.297	97	0.320	0.0158
	Monoarthritis. not elsewhere classified	M13.1	0	0.000	0	0.000	0	0.000	0	0.000	0.0000
	Monoarthritis. not elsewhere classified. unspecified site	M13.10	0	0.000	0	0.000	0	0.000	0	0.000	0.0000
	Monoarthritis. not elsewhere classified. shoulder	M13.11	2	0.013	2	0.017	0	0.000	4	0.013	0.8430
	Monoarthritis. not elsewhere classified. right shoulder	M13.111	0	0.000	0	0.000	0	0.000	0	0.000	0.0000
	Monoarthritis. not elsewhere classified. left shoulder	M13.112	0	0.000	0	0.000	0	0.000	0	0.000	0.0000
	Monoarthritis. not elsewhere classified. unspecified shoulder	M13.119	0	0.000	0	0.000	0	0.000	0	0.000	0.0000
	Monoarthritis. not elsewhere classified. elbow	M13.12	0	0.000	0	0.000	0	0.000	0	0.000	0.0000
	Monoarthritis. not elsewhere classified. right elbow	M13.121	0	0.000	0	0.000	0	0.000	0	0.000	0.0000
	Monoarthritis. not elsewhere classified. left elbow	M13.122	0	0.000	0	0.000	0	0.000	0	0.000	0.0000
	Monoarthritis. not elsewhere classified. unspecified elbow	M13.129	0	0.000	0	0.000	0	0.000	0	0.000	0.0000
	Monoarthritis. not elsewhere classified. wrist	M13.13	3	0.019	1	0.009	0	0.000	4	0.013	0.8430
	Monoarthritis. not elsewhere classified. right wrist	M13.131	0	0.000	0	0.000	0	0.000	0	0.000	0.0000
	Monoarthritis. not elsewhere classified. left wrist	M13.132	0	0.000	0	0.000	0	0.000	0	0.000	0.0000
	Monoarthritis. not elsewhere classified. unspecified wrist	M13.139	0	0.000	0	0.000	0	0.000	0	0.000	0.0000
	Monoarthritis. not elsewhere classified. hand	M13.14	7	0.044	2	0.017	0	0.000	9	0.030	0.3749
	Monoarthritis. not elsewhere classified. right hand	M13.141	0	0.000	0	0.000	0	0.000	0	0.000	0.0000
	Monoarthritis. not elsewhere classified. left hand	M13.142	0	0.000	0	0.000	0	0.000	0	0.000	0.0000
	Monoarthritis. not elsewhere classified. unspecified hand	M13.149	0	0.000	0	0.000	0	0.000	0	0.000	0.0000
	Monoarthritis. not elsewhere classified. hip	M13.15	0	0.000	0	0.000	0	0.000	0	0.000	0.0000
	Monoarthritis. not elsewhere classified. right hip	M13.151	0	0.000	0	0.000	0	0.000	0	0.000	0.0000
	Monoarthritis. not elsewhere classified. left hip	M13.152	0	0.000	0	0.000	0	0.000	0	0.000	0.0000
	Monoarthritis. not elsewhere classified. unspecified hip	M13.159	0	0.000	0	0.000	0	0.000	0	0.000	0.0000
	Monoarthritis. not elsewhere classified. knee	M13.16	16	0.100	7	0.060	4	0.149	27	0.089	0.3422

	Monoarthritis. not elsewhere classified. right knee	M13.161	0	0.000	0	0.000	0	0.000	0	0.000	0.0000
	Monoarthritis. not elsewhere classified. left knee	M13.162	0	0.000	0	0.000	0	0.000	0	0.000	0.0000
	Monoarthritis. not elsewhere classified. unspecified knee	M13.169	0	0.000	0	0.000	0	0.000	0	0.000	0.0000
	Monoarthritis. not elsewhere classified. ankle and foot	M13.17	9	0.057	5	0.043	2	0.074	16	0.053	0.8140
	Monoarthritis. not elsewhere classified. right ankle and foot	M13.171	0	0.000	0	0.000	0	0.000	0	0.000	0.0000
	Monoarthritis. not elsewhere classified. left ankle and foot	M13.172	0	0.000	0	0.000	0	0.000	0	0.000	0.0000
	Monoarthritis. not elsewhere classified. unspecified ankle and foot	M13.179	0	0.000	0	0.000	0	0.000	0	0.000	0.0000
	Other specified arthritis	M13.8	0	0.000	0	0.000	0	0.000	0	0.000	0.0000
	Other specified arthritis. unspecified site	M13.80	12	0.075	10	0.085	2	0.074	24	0.079	0.9402
	Other specified arthritis. shoulder	M13.81	3	0.019	1	0.009	0	0.000	4	0.013	0.8430
	Other specified arthritis. right shoulder	M13.811	0	0.000	0	0.000	0	0.000	0	0.000	0.0000
	Other specified arthritis. left shoulder	M13.812	0	0.000	0	0.000	0	0.000	0	0.000	0.0000
	Other specified arthritis. unspecified shoulder	M13.819	0	0.000	0	0.000	0	0.000	0	0.000	0.0000
	Other specified arthritis. elbow	M13.82	1	0.006	1	0.009	0	0.000	2	0.007	0.6187
	Other specified arthritis. right elbow	M13.821	0	0.000	0	0.000	0	0.000	0	0.000	0.0000
	Other specified arthritis. left elbow	M13.822	0	0.000	0	0.000	0	0.000	0	0.000	0.0000
	Other specified arthritis. unspecified elbow	M13.829	0	0.000	0	0.000	0	0.000	0	0.000	0.0000
	Other specified arthritis. wrist	M13.83	7	0.044	2	0.017	1	0.037	10	0.033	0.3749
	Other specified arthritis. right wrist	M13.831	0	0.000	0	0.000	0	0.000	0	0.000	0.0000
	Other specified arthritis. left wrist	M13.832	0	0.000	0	0.000	0	0.000	0	0.000	0.0000
	Other specified arthritis. unspecified wrist	M13.839	0	0.000	0	0.000	0	0.000	0	0.000	0.0000
	Other specified arthritis. hand	M13.84	6	0.038	5	0.043	1	0.037	12	0.040	0.9207
	Other specified arthritis. right hand	M13.841	0	0.000	0	0.000	0	0.000	0	0.000	0.0000
	Other specified arthritis. left hand	M13.842	0	0.000	0	0.000	0	0.000	0	0.000	0.0000
	Other specified arthritis. unspecified hand	M13.849	0	0.000	0	0.000	0	0.000	0	0.000	0.0000
	Other specified arthritis. hip	M13.85	5	0.031	3	0.026	1	0.037	9	0.030	0.9380
	Other specified arthritis. right hip	M13.851	0	0.000	0	0.000	0	0.000	0	0.000	0.0000
	Other specified arthritis. left hip	M13.852	0	0.000	0	0.000	0	0.000	0	0.000	0.0000
	Other specified arthritis. unspecified hip	M13.859	0	0.000	0	0.000	0	0.000	0	0.000	0.0000

	Other specified arthritis. knee	M13.86	28	0.176	10	0.085	2	0.074	40	0.132	0.0654
	Other specified arthritis. right knee	M13.861	0	0.000	0	0.000	0	0.000	0	0.000	0.0000
	Other specified arthritis. left knee	M13.862	0	0.000	0	0.000	0	0.000	0	0.000	0.0000
	Other specified arthritis. unspecified knee	M13.869	0	0.000	0	0.000	0	0.000	0	0.000	0.0000
	Other specified arthritis. ankle and foot	M13.87	16	0.100	7	0.060	4	0.149	27	0.089	0.3422
	Other specified arthritis. right ankle and foot	M13.871	0	0.000	0	0.000	0	0.000	0	0.000	0.0000
	Other specified arthritis. left ankle and foot	M13.872	0	0.000	0	0.000	0	0.000	0	0.000	0.0000
	Other specified arthritis. unspecified ankle and foot	M13.879	0	0.000	0	0.000	0	0.000	0	0.000	0.0000
	Other specified arthritis. other site	M13.88	7	0.044	3	0.026	0	0.000	10	0.033	0.6361
	Other specified arthritis. multiple sites	M13.89	23	0.144	8	0.068	1	0.037	32	0.105	0.0914
	Arthropathies in other specified diseases classified elsewhere	M14.8	15	0.094	3	0.026	0	0.000	18	0.059	0.0488
	Arthropathies in other specified diseases classified elsewhere. unspecified site	M14.80	0	0.000	0	0.000	0	0.000	0	0.000	0.0000
	Arthropathies in other specified diseases classified elsewhere. shoulder	M14.81	0	0.000	0	0.000	0	0.000	0	0.000	0.0000
	Arthropathies in other specified diseases classified elsewhere. right shoulder	M14.811	0	0.000	0	0.000	0	0.000	0	0.000	0.0000
	Arthropathies in other specified diseases classified elsewhere. left shoulder	M14.812	0	0.000	0	0.000	0	0.000	0	0.000	0.0000
	Arthropathies in other specified diseases classified elsewhere. unspecified shoulder	M14.819	0	0.000	0	0.000	0	0.000	0	0.000	0.0000
	Arthropathies in other specified diseases classified elsewhere. elbow	M14.82	0	0.000	0	0.000	0	0.000	0	0.000	0.0000
	Arthropathies in other specified diseases classified elsewhere. right elbow	M14.821	0	0.000	0	0.000	0	0.000	0	0.000	0.0000
	Arthropathies in other specified diseases classified elsewhere. left elbow	M14.822	0	0.000	0	0.000	0	0.000	0	0.000	0.0000
	Arthropathies in other specified diseases classified elsewhere. unspecified elbow	M14.829	0	0.000	0	0.000	0	0.000	0	0.000	0.0000
	Arthropathies in other specified diseases classified elsewhere. wrist	M14.83	0	0.000	0	0.000	0	0.000	0	0.000	0.0000
	Arthropathies in other specified diseases classified elsewhere. right wrist	M14.831	0	0.000	0	0.000	0	0.000	0	0.000	0.0000
	Arthropathies in other specified diseases classified elsewhere. left wrist	M14.832	0	0.000	0	0.000	0	0.000	0	0.000	0.0000
	Arthropathies in other specified diseases classified elsewhere. unspecified wrist	M14.839	0	0.000	0	0.000	0	0.000	0	0.000	0.0000
	Arthropathies in other specified diseases classified elsewhere. hand	M14.84	0	0.000	0	0.000	0	0.000	0	0.000	0.0000
	Arthropathies in other specified diseases classified elsewhere. right hand	M14.841	0	0.000	0	0.000	0	0.000	0	0.000	0.0000

	Arthropathies in other specified diseases classified elsewhere. left hand	M14.842	0	0.000	0	0.000	0	0.000	0	0.000	0.0000
	Arthropathies in other specified diseases classified elsewhere. unspecified hand	M14.849	0	0.000	0	0.000	0	0.000	0	0.000	0.0000
	Arthropathies in other specified diseases classified elsewhere. hip	M14.85	0	0.000	0	0.000	0	0.000	0	0.000	0.0000
	Arthropathies in other specified diseases classified elsewhere. right hip	M14.851	0	0.000	0	0.000	0	0.000	0	0.000	0.0000
	Arthropathies in other specified diseases classified elsewhere. left hip	M14.852	0	0.000	0	0.000	0	0.000	0	0.000	0.0000
	Arthropathies in other specified diseases classified elsewhere. unspecified hip	M14.859	0	0.000	0	0.000	0	0.000	0	0.000	0.0000
	Arthropathies in other specified diseases classified elsewhere. knee	M14.86	0	0.000	0	0.000	0	0.000	0	0.000	0.0000
	Arthropathies in other specified diseases classified elsewhere. right knee	M14.861	0	0.000	0	0.000	0	0.000	0	0.000	0.0000
	Arthropathies in other specified diseases classified elsewhere. left knee	M14.862	0	0.000	0	0.000	0	0.000	0	0.000	0.0000
	Arthropathies in other specified diseases classified elsewhere. unspecified knee	M14.869	0	0.000	0	0.000	0	0.000	0	0.000	0.0000
	Arthropathies in other specified diseases classified elsewhere. ankle and foot	M14.87	0	0.000	0	0.000	0	0.000	0	0.000	0.0000
	Arthropathies in other specified diseases classified elsewhere. right ankle and foot	M14.871	0	0.000	0	0.000	0	0.000	0	0.000	0.0000
	Arthropathies in other specified diseases classified elsewhere. left ankle and foot	M14.872	0	0.000	0	0.000	0	0.000	0	0.000	0.0000
	Arthropathies in other specified diseases classified elsewhere. unspecified ankle and foot	M14.879	0	0.000	0	0.000	0	0.000	0	0.000	0.0000
	Arthropathies in other specified diseases classified elsewhere. vertebrae	M14.88	0	0.000	0	0.000	0	0.000	0	0.000	0.0000
	Arthropathies in other specified diseases classified elsewhere. multiple sites	M14.89	0	0.000	0	0.000	0	0.000	0	0.000	0.0000
	**Polyarteritis nodosa and related conditions**	**M30**	0	0.000	0	0.000	0	0.000	0	0.000	0.0000
	Polyarteritis nodosa	M30.0	7	0.044	2	0.017	1	0.037	10	0.033	0.3749
	Polyarteritis with lung involvement [Churg-Strauss]	M30.1	3	0.019	3	0.026	0	0.000	6	0.020	0.9713
	Juvenile polyarteritis	M30.2	0	0.000	0	0.000	0	0.000	0	0.000	0.0000
	Mucocutaneous lymph node syndrome [Kawasaki]	M30.3	5	0.031	2	0.017	0	0.000	7	0.023	0.7207
	Other conditions related to polyarteritis nodosa	M30.8	0	0.000	2	0.017	0	0.000	2	0.007	0.3507
	**Other necrotizing vasculopathies**	**M31**	0	0.000	0	0.000	0	0.000	0	0.000	0.0000
	Hypersensitivity angiitis	M31.0	7	0.044	4	0.034	1	0.037	12	0.040	0.9207
	Thrombotic microangiopathy	M31.1	4	0.025	4	0.034	2	0.074	10	0.033	0.9380

	Thrombotic microangiopathy. unspecified	M31.10	0	0.000	0	0.000	0	0.000	0	0.000	0.0000
	Hematopoietic stem cell transplantation-associated thrombotic microangiopathy [HSCT-TMA]	M31.11	0	0.000	0	0.000	0	0.000	0	0.000	0.0000
	Other thrombotic microangiopathy	M31.19	0	0.000	0	0.000	0	0.000	0	0.000	0.0000
	Lethal midline granuloma	M31.2	0	0.000	1	0.009	0	0.000	1	0.003	0.8776
	Wegener's granulomatosis	M31.3	22	0.138	11	0.094	4	0.149	37	0.122	0.3803
	Wegener's granulomatosis without renal involvement	M31.30	0	0.000	1	0.009	0	0.000	1	0.003	0.8776
	Wegener's granulomatosis with renal involvement	M31.31	0	0.000	0	0.000	0	0.000	0	0.000	0.0000
	Aortic arch syndrome [Takayasu]	M31.4	8	0.050	5	0.043	0	0.000	13	0.043	0.9951
	Giant cell arteritis with polymyalgia rheumatica	M31.5	2	0.013	1	0.009	1	0.037	4	0.013	0.7897
	Other giant cell arteritis	M31.6	31	0.195	23	0.196	9	0.334	63	0.208	0.9140
	Microscopic polyangiitis	M31.7	1	0.006	1	0.009	0	0.000	2	0.007	0.6187
	Other specified necrotizing vasculopathies	M31.8	2	0.013	1	0.009	1	0.037	4	0.013	0.7897
	Necrotizing vasculopathy. unspecified	M31.9	4	0.025	2	0.017	1	0.037	7	0.023	0.9713
	**Systemic lupus erythematosus**	**M32**	0	0.000	0	0.000	0	0.000	0	0.000	0.0000
	Drug-induced systemic lupus erythematosus	M32.0	12	0.075	2	0.017	0	0.000	14	0.046	0.0632
	Systemic lupus erythematosus with organ or system involvement	M32.1	9	0.057	8	0.068	2	0.074	19	0.063	0.8855
	Systemic lupus erythematosus. organ or system involvement unspecified	M32.10	0	0.000	0	0.000	0	0.000	0	0.000	0.0000
	Endocarditis in systemic lupus erythematosus	M32.11	0	0.000	0	0.000	0	0.000	0	0.000	0.0000
	Pericarditis in systemic lupus erythematosus	M32.12	0	0.000	0	0.000	0	0.000	0	0.000	0.0000
	Lung involvement in systemic lupus erythematosus	M32.13	0	0.000	0	0.000	0	0.000	0	0.000	0.0000
	Glomerular disease in systemic lupus erythematosus	M32.14	0	0.000	0	0.000	0	0.000	0	0.000	0.0000
	Tubulo-interstitial nephropathy in systemic lupus erythematosus	M32.15	0	0.000	0	0.000	0	0.000	0	0.000	0.0000
	Other organ or system involvement in systemic lupus erythematosus	M32.19	0	0.000	0	0.000	0	0.000	0	0.000	0.0000
	Other forms of systemic lupus erythematosus	M32.8	3	0.019	1	0.009	1	0.037	5	0.016	0.8430
	Systemic lupus erythematosus. unspecified	M32.9	125	0.785	61	0.521	19	0.706	205	0.676	0.0098
	**Dermatopolymyositis**	**M33**	0	0.000	0	0.000	0	0.000	0	0.000	0.0000
	Juvenile dermatomyositis	M33.0	1	0.006	0	0.000	0	0.000	1	0.003	0.8776
	Juvenile dermatomyositis. organ involvement unspecified	M33.00	0	0.000	0	0.000	0	0.000	0	0.000	0.0000

	Juvenile dermatomyositis with respiratory involvement	M33.01	0	0.000	0	0.000	0	0.000	0	0.000	0.0000
	Juvenile dermatomyositis with myopathy	M33.02	0	0.000	0	0.000	0	0.000	0	0.000	0.0000
	Juvenile dermatomyositis without myopathy	M33.03	0	0.000	0	0.000	0	0.000	0	0.000	0.0000
	Juvenile dermatomyositis with other organ involvement	M33.09	0	0.000	0	0.000	0	0.000	0	0.000	0.0000
	Other dermatomyositis	M33.1	13	0.082	8	0.068	2	0.074	23	0.076	0.8589
	Other dermatomyositis. organ involvement unspecified	M33.10	0	0.000	0	0.000	0	0.000	0	0.000	0.0000
	Other dermatomyositis with respiratory involvement	M33.11	0	0.000	0	0.000	0	0.000	0	0.000	0.0000
	Other dermatomyositis with myopathy	M33.12	0	0.000	0	0.000	0	0.000	0	0.000	0.0000
	Other dermatomyositis without myopathy	M33.13	0	0.000	0	0.000	0	0.000	0	0.000	0.0000
	Other dermatomyositis with other organ involvement	M33.19	0	0.000	0	0.000	0	0.000	0	0.000	0.0000
	Polymyositis	M33.2	7	0.044	10	0.085	0	0.000	17	0.056	0.2602
	Polymyositis. organ involvement unspecified	M33.20	0	0.000	0	0.000	0	0.000	0	0.000	0.0000
	Polymyositis with respiratory involvement	M33.21	0	0.000	0	0.000	0	0.000	0	0.000	0.0000
	Polymyositis with myopathy	M33.22	0	0.000	0	0.000	0	0.000	0	0.000	0.0000
	Polymyositis with other organ involvement	M33.29	0	0.000	0	0.000	0	0.000	0	0.000	0.0000
	Dermatopolymyositis. unspecified	M33.9	2	0.013	1	0.009	0	0.000	3	0.010	0.7897
	Dermatopolymyositis. unspecified. organ involvement unspecified	M33.90	0	0.000	0	0.000	0	0.000	0	0.000	0.0000
	Dermatopolymyositis. unspecified with respiratory involvement	M33.91	0	0.000	0	0.000	0	0.000	0	0.000	0.0000
	Dermatopolymyositis. unspecified with myopathy	M33.92	0	0.000	0	0.000	0	0.000	0	0.000	0.0000
	Dermatopolymyositis. unspecified without myopathy	M33.93	0	0.000	0	0.000	0	0.000	0	0.000	0.0000
	Dermatopolymyositis. unspecified with other organ involvement	M33.99	0	0.000	0	0.000	0	0.000	0	0.000	0.0000
	**Systemic sclerosis [scleroderma]**	**M34**	0	0.000	0	0.000	0	0.000	0	0.000	0.0000
	Progressive systemic sclerosis	M34.0	0	0.000	1	0.009	0	0.000	1	0.003	0.8776
	CREST syndrome	M34.1	6	0.038	1	0.009	0	0.000	7	0.023	0.2617
	Systemic sclerosis induced by drug and chemical	M34.2	0	0.000	0	0.000	0	0.000	0	0.000	0.0000
	Other forms of systemic sclerosis	M34.8	2	0.013	0	0.000	0	0.000	2	0.007	0.6187
	Systemic sclerosis with lung involvement	M34.81	0	0.000	0	0.000	0	0.000	0	0.000	0.0000
	Systemic sclerosis with myopathy	M34.82	0	0.000	0	0.000	0	0.000	0	0.000	0.0000
	Systemic sclerosis with polyneuropathy	M34.83	0	0.000	0	0.000	0	0.000	0	0.000	0.0000

	Other systemic sclerosis	M34.89	0	0.000	0	0.000	0	0.000	0	0.000	0.0000
	Systemic sclerosis. unspecified	M34.9	32	0.201	7	0.060	2	0.074	41	0.135	0.0034
	**Other systemic involvement of connective tissue**	**M35**	0	0.000	0	0.000	0	0.000	0	0.000	0.0000
	Sjögren syndrome	M35.0	35	0.220	19	0.162	5	0.186	59	0.195	0.3498
	Sjögren syndrome. unspecified	M35.00	0	0.000	0	0.000	0	0.000	0	0.000	0.0000
	Sjögren syndrome with keratoconjunctivitis	M35.01	0	0.000	0	0.000	0	0.000	0	0.000	0.0000
	Sjögren syndrome with lung involvement	M35.02	0	0.000	0	0.000	0	0.000	0	0.000	0.0000
	Sjögren syndrome with myopathy	M35.03	0	0.000	0	0.000	0	0.000	0	0.000	0.0000
	Sjögren syndrome with tubulo-interstitial nephropathy	M35.04	0	0.000	0	0.000	0	0.000	0	0.000	0.0000
	Sjögren syndrome with inflammatory arthritis	M35.05	0	0.000	0	0.000	0	0.000	0	0.000	0.0000
	Sögren syndrome with peripheral nervous system involvement	M35.06	0	0.000	0	0.000	0	0.000	0	0.000	0.0000
	Sjögren syndrome with central nervous system involvement	M35.07	0	0.000	0	0.000	0	0.000	0	0.000	0.0000
	Sjögren syndrome with gastrointestinal involvement	M35.08	0	0.000	0	0.000	0	0.000	0	0.000	0.0000
	Sjögren syndrome with glomerular disease	M35.0A	0	0.000	0	0.000	0	0.000	0	0.000	0.0000
	Sjögren syndrome with vasculitis	M35.0B	0	0.000	0	0.000	0	0.000	0	0.000	0.0000
	Sjögren syndrome with dental involvement	M35.0C	0	0.000	0	0.000	0	0.000	0	0.000	0.0000
	Sjögren syndrome with other organ involvement	M35.09	0	0.000	0	0.000	0	0.000	0	0.000	0.0000
	Other overlap syndromes	M35.1	2	0.013	3	0.026	1	0.037	6	0.020	0.7306
	Behcet's disease	M35.2	32	0.201	10	0.085	4	0.149	46	0.152	0.0225
	Polymyalgia rheumatica	M35.3	54	0.339	43	0.367	16	0.594	113	0.373	0.7764
	Diffuse	M35.4	0	0.000	0	0.000	0	0.000	0	0.000	0.0000
	Multifocal fibrosclerosis	M35.5	1	0.006	0	0.000	0	0.000	1	0.003	0.8776
	Relapsing panniculitis [Weber-Christian]	M35.6	0	0.000	0	0.000	0	0.000	0	0.000	0.0000
	Hypermobility syndrome	M35.7	25	0.157	21	0.179	6	0.223	52	0.171	0.7653
	Other specified systemic involvement of connective tissue	M35.8	4	0.025	10	0.085	1	0.037	15	0.049	0.0538
	Multisystem inflammatory syndrome	M35.81	0	0.000	0	0.000	0	0.000	0	0.000	0.0000
	Other specified systemic involvement of connective tissue	M35.89	0	0.000	0	0.000	0	0.000	0	0.000	0.0000
	Systemic involvement of connective tissue. unspecified	M35.9	148	0.929	67	0.572	22	0.817	237	0.781	0.0011
	**Ankylosing spondylitis**	**M45**	560	3.517	170	1.451	55	2.043	785	2.588	0.0000

	Ankylosing spondylitis of multiple sites in spine	M45.0	0	0.000	0	0.000	0	0.000	0	0.000	0.0000
	Ankylosing spondylitis of occipito-atlanto-axial region	M45.1	0	0.000	0	0.000	0	0.000	0	0.000	0.0000
	Ankylosing spondylitis of cervical region	M45.2	0	0.000	0	0.000	0	0.000	0	0.000	0.0000
	Ankylosing spondylitis of cervicothoracic region	M45.3	1	0.006	0	0.000	0	0.000	1	0.003	0.8776
	Ankylosing spondylitis of thoracic region	M45.4	0	0.000	0	0.000	0	0.000	0	0.000	0.0000
	Ankylosing spondylitis of thoracolumbar region	M45.5	0	0.000	0	0.000	0	0.000	0	0.000	0.0000
	Ankylosing spondylitis lumbar region	M45.6	0	0.000	0	0.000	0	0.000	0	0.000	0.0000
	Ankylosing spondylitis of lumbosacral region	M45.7	0	0.000	0	0.000	0	0.000	0	0.000	0.0000
	Ankylosing spondylitis sacral and sacrococcygeal region	M45.8	0	0.000	0	0.000	0	0.000	0	0.000	0.0000
	Ankylosing spondylitis of unspecified sites in spine	M45.9	0	0.000	0	0.000	0	0.000	0	0.000	0.0000
	Non-radiographic axial spondyloarthritis	M45.A	0	0.000	0	0.000	0	0.000	0	0.000	0.0000
	Non-radiographic axial spondyloarthritis of unspecified sites in spine	M45.A0	0	0.000	0	0.000	0	0.000	0	0.000	0.0000
	Non-radiographic axial spondyloarthritis of occipito-atlanto-axial region	M45.A1	0	0.000	0	0.000	0	0.000	0	0.000	0.0000
	Non-radiographic axial spondyloarthritis of cervical region	M45.A2	0	0.000	0	0.000	0	0.000	0	0.000	0.0000
	Non-radiographic axial spondyloarthritis of cervicothoracic region	M45.A3	0	0.000	0	0.000	0	0.000	0	0.000	0.0000
	Non-radiographic axial spondyloarthritis of thoracic region	M45.A4	0	0.000	0	0.000	0	0.000	0	0.000	0.0000
	Non-radiographic axial spondyloarthritis of thoracolumbar region	M45.A5	0	0.000	0	0.000	0	0.000	0	0.000	0.0000
	Non-radiographic axial spondyloarthritis of lumbar region	M45.A6	0	0.000	0	0.000	0	0.000	0	0.000	0.0000
	Non-radiographic axial spondyloarthritis of lumbosacral region	M45.A7	0	0.000	0	0.000	0	0.000	0	0.000	0.0000
	Non-radiographic axial spondyloarthritis of sacral and sacrococcygeal region	M45.A8	0	0.000	0	0.000	0	0.000	0	0.000	0.0000
	Non-radiographic axial spondyloarthritis of multiple sites in spine	M45.AB	0	0.000	0	0.000	0	0.000	0	0.000	0.0000
	**Other inflammatory spondylopathies**	**M46**	0	0.000	0	0.000	0	0.000	0	0.000	0.0000
	Spinal enthesopathy	M46.0	0	0.000	0	0.000	0	0.000	0	0.000	0.0000
	Spinal enthesopathy. site unspecified	M46.00	0	0.000	0	0.000	0	0.000	0	0.000	0.0000
	Spinal enthesopathy. occipito-atlanto-axial region	M46.01	0	0.000	0	0.000	0	0.000	0	0.000	0.0000
	Spinal enthesopathy. cervical region	M46.02	0	0.000	0	0.000	0	0.000	0	0.000	0.0000
	Spinal enthesopathy. cervicothoracic region	M46.03	0	0.000	0	0.000	0	0.000	0	0.000	0.0000
	Spinal enthesopathy. thoracic region	M46.04	0	0.000	0	0.000	0	0.000	0	0.000	0.0000

	Spinal enthesopathy. thoracolumbar region	M46.05	0	0.000	0	0.000	0	0.000	0	0.000	0.0000
	Spinal enthesopathy. lumbar region	M46.06	0	0.000	1	0.009	0	0.000	1	0.003	0.8776
	Spinal enthesopathy. lumbosacral region	M46.07	0	0.000	0	0.000	0	0.000	0	0.000	0.0000
	Spinal enthesopathy. sacral and sacrococcygeal region	M46.08	0	0.000	0	0.000	0	0.000	0	0.000	0.0000
	Spinal enthesopathy. multiple sites in spine	M46.09	0	0.000	0	0.000	0	0.000	0	0.000	0.0000
	Sacroiliitis. not elsewhere classified	M46.1	153	0.961	69	0.589	15	0.557	237	0.781	0.0008
	Other specified inflammatory spondylopathies	M46.8	0	0.000	0	0.000	0	0.000	0	0.000	0.0000
	Other specified inflammatory spondylopathies. site unspecified	M46.80	0	0.000	0	0.000	0	0.000	0	0.000	0.0000
	Other specified inflammatory spondylopathies. occipito-atlanto-axial region	M46.81	0	0.000	0	0.000	0	0.000	0	0.000	0.0000
	Other specified inflammatory spondylopathies. cervical region	M46.82	0	0.000	0	0.000	1	0.037	1	0.003	0.0000
	Other specified inflammatory spondylopathies. cervicothoracic region	M46.83	0	0.000	0	0.000	0	0.000	0	0.000	0.0000
	Other specified inflammatory spondylopathies. thoracic region	M46.84	1	0.006	0	0.000	0	0.000	1	0.003	0.8776
	Other specified inflammatory spondylopathies. thoracolumbar region	M46.85	0	0.000	0	0.000	0	0.000	0	0.000	0.0000
	Other specified inflammatory spondylopathies. lumbar region	M46.86	0	0.000	1	0.009	0	0.000	1	0.003	0.8776
	Other specified inflammatory spondylopathies. lumbosacral region	M46.87	0	0.000	0	0.000	0	0.000	0	0.000	0.0000
	Other specified inflammatory spondylopathies. sacral and sacrococcygeal region	M46.88	1	0.006	0	0.000	0	0.000	1	0.003	0.8776
	Other specified inflammatory spondylopathies. multiple sites in spine	M46.89	2	0.013	0	0.000	0	0.000	2	0.007	0.6187
	Unspecified inflammatory spondylopathy	M46.9	0	0.000	0	0.000	0	0.000	0	0.000	0.0000
	Unspecified inflammatory spondylopathy. site unspecified	M46.90	1	0.006	0	0.000	0	0.000	1	0.003	0.8776
	Unspecified inflammatory spondylopathy. occipito-atlanto-axial region	M46.91	0	0.000	0	0.000	0	0.000	0	0.000	0.0000
	Unspecified inflammatory spondylopathy. cervical region	M46.92	10	0.063	4	0.034	0	0.000	14	0.046	0.4376
	Unspecified inflammatory spondylopathy. cervicothoracic region	M46.93	0	0.000	0	0.000	0	0.000	0	0.000	0.0000
	Unspecified inflammatory spondylopathy. thoracic region	M46.94	1	0.006	1	0.009	0	0.000	2	0.007	0.6187
	Unspecified inflammatory spondylopathy. thoracolumbar region	M46.95	0	0.000	0	0.000	0	0.000	0	0.000	0.0000
	Unspecified inflammatory spondylopathy. lumbar region	M46.96	5	0.031	8	0.068	1	0.037	14	0.046	0.2642
	Unspecified inflammatory spondylopathy. lumbosacral region	M46.97	3	0.019	1	0.009	0	0.000	4	0.013	0.8430

	Unspecified inflammatory spondylopathy. sacral and sacrococcygeal region	M46.98	0	0.000	2	0.017	0	0.000	2	0.007	0.3507
	Unspecified inflammatory spondylopathy. multiple sites in spine	M46.99	33	0.207	10	0.085	1	0.037	44	0.145	0.0170
	**Rheumatism. unspecified**	**M79.0**	0	0.000	0	0.000	0	0.000	0	0.000	0.0000
	Myalgia	M79.1	0	0.000	0	0.000	0	0.000	0	0.000	0.0000
	Myalgia. unspecified site	M79.10	138	0.867	70	0.597	18	0.669	226	0.745	0.0128
	Myalgia of mastication muscle	M79.11	69	0.433	46	0.393	8	0.297	123	0.405	0.6704
	Myalgia of auxiliary muscles. head and neck	M79.12	17	0.107	18	0.154	2	0.074	37	0.122	0.3621
	Myalgia. other site	M79.18	213	1.338	126	1.075	47	1.746	386	1.272	0.0570
	Neuralgia and neuritis. unspecified	M79.2	0	0.000	0	0.000	0	0.000	0	0.000	0.0000
	Panniculitis. unspecified	M79.3	0	0.000	0	0.000	0	0.000	0	0.000	0.0000
	Hypertrophy of	M79.4	10	0.063	3	0.026	0	0.000	13	0.043	0.2589
	Residual foreign body in soft tissue	M79.5	0	0.000	0	0.000	0	0.000	0	0.000	0.0000
	Pain in limb. hand. foot. fingers and toes	M79.6	0	0.000	0	0.000	0	0.000	0	0.000	0.0000
	Pain in limb. unspecified	M79.60	423	2.656	258	2.202	84	3.120	765	2.522	0.0178
	Pain in right arm	M79.601	0	0.000	0	0.000	0	0.000	0	0.000	0.0000
	Pain in left arm	M79.602	0	0.000	0	0.000	0	0.000	0	0.000	0.0000
	Pain in arm. unspecified	M79.603	0	0.000	0	0.000	0	0.000	0	0.000	0.0000
	Pain in right leg	M79.604	0	0.000	0	0.000	0	0.000	0	0.000	0.0000
	Pain in left leg	M79.605	0	0.000	0	0.000	0	0.000	0	0.000	0.0000
	Pain in leg. unspecified	M79.606	0	0.000	0	0.000	0	0.000	0	0.000	0.0000
	Pain in unspecified limb	M79.609	0	0.000	0	0.000	0	0.000	0	0.000	0.0000
	Pain in upper arm	M79.62	0	0.000	0	0.000	0	0.000	0	0.000	0.0000
	Pain in right upper arm	M79.621	0	0.000	0	0.000	0	0.000	0	0.000	0.0000
	Pain in left upper arm	M79.622	0	0.000	0	0.000	0	0.000	0	0.000	0.0000
	Pain in unspecified upper arm	M79.629	0	0.000	0	0.000	0	0.000	0	0.000	0.0000
	Pain in forearm	M79.63	0	0.000	0	0.000	0	0.000	0	0.000	0.0000
	Pain in right forearm	M79.631	0	0.000	0	0.000	0	0.000	0	0.000	0.0000
	Pain in left forearm	M79.632	0	0.000	0	0.000	0	0.000	0	0.000	0.0000
	Pain in unspecified forearm	M79.639	0	0.000	0	0.000	0	0.000	0	0.000	0.0000

	Pain in hand and fingers	M79.64	0	0.000	0	0.000	0	0.000	0	0.000	0.0000
	Pain in right hand	M79.641	0	0.000	0	0.000	0	0.000	0	0.000	0.0000
	Pain in left hand	M79.642	0	0.000	0	0.000	0	0.000	0	0.000	0.0000
	Pain in unspecified hand	M79.643	0	0.000	0	0.000	0	0.000	0	0.000	0.0000
	Pain in right finge	M79.644	0	0.000	0	0.000	0	0.000	0	0.000	0.0000
	Pain in left finge	M79.645	0	0.000	0	0.000	0	0.000	0	0.000	0.0000
	Pain in unspecified finge	M79.646	0	0.000	0	0.000	0	0.000	0	0.000	0.0000
	Pain in thigh	M79.65	0	0.000	0	0.000	0	0.000	0	0.000	0.0000
	Pain in right thigh	M79.651	0	0.000	0	0.000	0	0.000	0	0.000	0.0000
	Pain in left thigh	M79.652	0	0.000	0	0.000	0	0.000	0	0.000	0.0000
	Pain in unspecified thigh	M79.659	0	0.000	0	0.000	0	0.000	0	0.000	0.0000
	Pain in lower leg	M79.66	0	0.000	0	0.000	0	0.000	0	0.000	0.0000
	Pain in right lower leg	M79.661	0	0.000	0	0.000	0	0.000	0	0.000	0.0000
	Pain in left lower leg	M79.662	0	0.000	0	0.000	0	0.000	0	0.000	0.0000
	Pain in unspecified lower leg	M79.669	0	0.000	0	0.000	0	0.000	0	0.000	0.0000
	Pain in foot and toes	M79.67	0	0.000	0	0.000	0	0.000	0	0.000	0.0000
	Pain in right foot	M79.671	0	0.000	0	0.000	0	0.000	0	0.000	0.0000
	Pain in left foot	M79.672	0	0.000	0	0.000	0	0.000	0	0.000	0.0000
	Pain in unspecified foot	M79.673	0	0.000	0	0.000	0	0.000	0	0.000	0.0000
	Pain in right to	M79.674	0	0.000	0	0.000	0	0.000	0	0.000	0.0000
	Pain in left to	M79.675	0	0.000	0	0.000	0	0.000	0	0.000	0.0000
	Pain in unspecified to	M79.676	0	0.000	0	0.000	0	0.000	0	0.000	0.0000
	Fibromyalgia	M79.7	253	1.589	105	0.896	61	2.266	419	1.381	0.0000
**Total**			3142	19.731	1767	15.079	467	17.348	5376	17.723	0.0000

Supplementary Table 3: Diseases of the genitourinary system

Organ System	Extraintestinal Manifestations and Associated Immune Disorders	ICD-10	Crohn's Disease		Ulcerative Colitis		IBD-Unclassified		IBD-Total (CD + UC + IBDU)		Comparison
			N = 15924		N = 11718		N = 2692		N = 30334		UC vs. CD
			n	%	n	%	n	%	n	%	p value ≤
**Diseases of the genitourinary system**											
	**Acute nephritic syndrome**	**N00**	0	0.000	0	0.000	0	0.000	0	0.000	0.0000
	Acute nephritic syndrome with minor glomerular abnormality	N00.0	1	0.006	0	0.000	0	0.000	1	0.003	0.8776
	Acute nephritic syndrome with focal and segmental glomerular lesions	N00.1	0	0.000	0	0.000	0	0.000	0	0.000	0.0000
	Acute nephritic syndrome with diffuse membranous glomerulonephritis	N00.2	0	0.000	0	0.000	0	0.000	0	0.000	0.0000
	Acute nephritic syndrome with diffuse mesangial proliferative glomerulonephritis	N00.3	0	0.000	0	0.000	0	0.000	0	0.000	0.0000
	Acute nephritic syndrome with diffuse endocapillary proliferative glomerulonephritis	N00.4	1	0.006	0	0.000	0	0.000	1	0.003	0.8776
	Acute nephritic syndrome with diffuse mesangiocapillary glomerulonephritis	N00.5	0	0.000	0	0.000	0	0.000	0	0.000	0.0000
	Acute nephritic syndrome with dense deposit disease	N00.6	0	0.000	0	0.000	0	0.000	0	0.000	0.0000
	Acute nephritic syndrome with diffuse crescentic glomerulonephritis	N00.7	0	0.000	0	0.000	0	0.000	0	0.000	0.0000
	Acute nephritic syndrome with other morphologic changes	N00.8	2	0.013	2	0.017	0	0.000	4	0.013	0.8430
	Acute nephritic syndrome with unspecified morphologic changes	N00.9	8	0.050	7	0.060	3	0.111	18	0.059	0.9412
	Acute nephritic syndrome with C3 glomerulonephritis	N00.A	0	0.000	0	0.000	0	0.000	0	0.000	0.0000
	**Rapidly progressive nephritic syndrome**	**N01**	0	0.000	0	0.000	0	0.000	0	0.000	0.0000
	Rapidly progressive nephritic syndrome with minor glomerular abnormality	N01.0	0	0.000	0	0.000	0	0.000	0	0.000	0.0000
	Rapidly progressive nephritic syndrome with focal and segmental glomerular lesions	N01.1	0	0.000	0	0.000	0	0.000	0	0.000	0.0000
	Rapidly progressive nephritic syndrome with diffuse membranous glomerulonephritis	N01.2	0	0.000	0	0.000	0	0.000	0	0.000	0.0000
	Rapidly progressive nephritic syndrome with diffuse mesangial proliferative glomerulonephritis	N01.3	0	0.000	0	0.000	0	0.000	0	0.000	0.0000
	Rapidly progressive nephritic syndrome with diffuse endocapillary proliferative glomerulonephritis	N01.4	0	0.000	0	0.000	0	0.000	0	0.000	0.0000
	Rapidly progressive nephritic syndrome with diffuse mesangiocapillary glomerulonephritis	N01.5	0	0.000	0	0.000	0	0.000	0	0.000	0.0000
	Rapidly progressive nephritic syndrome with dense deposit disease	N01.6	0	0.000	0	0.000	0	0.000	0	0.000	0.0000

	Rapidly progressive nephritic syndrome with diffuse crescentic glomerulonephritis	N01.7	0	0.000	0	0.000	0	0.000	0	0.000	0.0000
	Rapidly progressive nephritic syndrome with other morphologic changes	N01.8	0	0.000	0	0.000	0	0.000	0	0.000	0.0000
	Rapidly progressive nephritic syndrome with unspecified morphologic changes	N01.9	0	0.000	1	0.009	0	0.000	1	0.003	0.8776
	Rapidly progressive nephritic syndrome with C3 glomerulonephritis	N01.A	0	0.000	0	0.000	0	0.000	0	0.000	0.0000
	**Chronic nephritic syndrome**	**N03**	0	0.000	0	0.000	0	0.000	0	0.000	0.0000
	Chronic nephritic syndrome with minor glomerular abnormality	N03.0	0	0.000	0	0.000	0	0.000	0	0.000	0.0000
	Chronic nephritic syndrome with focal and segmental glomerular lesions	N03.1	1	0.006	1	0.009	0	0.000	2	0.007	0.6187
	Chronic nephritic syndrome with diffuse membranous glomerulonephritis	N03.2	0	0.000	0	0.000	1	0.037	1	0.003	0.0000
	Chronic nephritic syndrome with diffuse mesangial proliferative glomerulonephritis	N03.3	0	0.000	0	0.000	0	0.000	0	0.000	0.0000
	Chronic nephritic syndrome with diffuse endocapillary proliferative glomerulonephritis	N03.4	0	0.000	0	0.000	0	0.000	0	0.000	0.0000
	Chronic nephritic syndrome with diffuse mesangiocapillary glomerulonephritis	N03.5	0	0.000	1	0.009	0	0.000	1	0.003	0.8776
	Chronic nephritic syndrome with dense deposit disease	N03.6	0	0.000	0	0.000	0	0.000	0	0.000	0.0000
	Chronic nephritic syndrome with diffuse crescentic glomerulonephritis	N03.7	0	0.000	0	0.000	0	0.000	0	0.000	0.0000
	Chronic nephritic syndrome with other morphologic changes	N03.8	1	0.006	0	0.000	1	0.037	2	0.007	0.8776
	Chronic nephritic syndrome with unspecified morphologic changes	N03.9	14	0.088	4	0.034	1	0.037	19	0.063	0.1353
	Chronic nephritic syndrome with C3 glomerulonephritis	N03.A	0	0.000	0	0.000	0	0.000	0	0.000	0.0000
	**Unspecified nephritic syndrome**	**N05**	0	0.000	0	0.000	0	0.000	0	0.000	0.0000
	Unspecified nephritic syndrome with minor glomerular abnormality	N05.0	0	0.000	0	0.000	0	0.000	0	0.000	0.0000
	Unspecified nephritic syndrome with focal and segmental glomerular lesions	N05.1	0	0.000	2	0.017	1	0.037	3	0.010	0.3507
	Unspecified nephritic syndrome with diffuse membranous glomerulonephritis	N05.2	1	0.006	1	0.009	1	0.037	3	0.010	0.6187
	Unspecified nephritic syndrome with diffuse mesangial proliferative glomerulonephritis	N05.3	0	0.000	0	0.000	0	0.000	0	0.000	0.0000
	Unspecified nephritic syndrome with diffuse endocapillary proliferative glomerulonephritis	N05.4	0	0.000	1	0.009	0	0.000	1	0.003	0.8776
	Unspecified nephritic syndrome with diffuse mesangiocapillary glomerulonephritis	N05.5	1	0.006	2	0.017	1	0.037	4	0.013	0.7897
	Unspecified nephritic syndrome with dense deposit disease	N05.6	0	0.000	0	0.000	0	0.000	0	0.000	0.0000
	Unspecified nephritic syndrome with diffuse crescentic glomerulonephritis	N05.7	2	0.013	1	0.009	0	0.000	3	0.010	0.7897
	Unspecified nephritic syndrome with other morphologic changes	N05.8	6	0.038	3	0.026	1	0.037	10	0.033	0.8316
	Unspecified nephritic syndrome with unspecified morphologic changes	N05.9	63	0.396	57	0.486	14	0.520	134	0.442	0.2973
	Unspecified nephritic syndrome with C3 glomerulonephritis	N05.A	0	0.000	0	0.000	0	0.000	0	0.000	0.0000
	Other chronic tubulo-interstitial nephritis	N11.8	0	0.000	3	0.026	0	0.000	3	0.010	0.1513

	Chronic tubulo-interstitial nephritis. unspecified	N11.9	10	0.063	3	0.026	2	0.074	15	0.049	0.2589
	Tubulo-interstitial nephritis. not specified as acute or chronic	N12	748	4.697	425	3.627	133	4.941	1306	4.305	0.0000
	**Calculus of kidney and ureter**	**N20**	0	0.000	0	0.000	0	0.000	0	0.000	0.0000
	Calculus of kidney	N20.0	1003	6.299	467	3.985	116	4.309	1586	5.228	0.0000
	Calculus of ureter	N20.1	625	3.925	295	2.517	63	2.340	983	3.241	0.0000
	Calculus of kidney with calculus of ureter	N20.2	217	1.363	85	0.725	21	0.780	323	1.065	0.0000
	Urinary calculus. unspecified	N20.9	115	0.722	49	0.418	12	0.446	176	0.580	0.0015
	**Calculus of lower urinary tract**	**N21**	0	0.000	0	0.000	0	0.000	0	0.000	0.0000
	Calculus in bladder	N21.0	131	0.823	62	0.529	13	0.483	206	0.679	0.0048
	Calculus in urethra	N21.1	19	0.119	11	0.094	4	0.149	34	0.112	0.6526
	Other lower urinary tract calculus	N21.8	0	0.000	0	0.000	0	0.000	0	0.000	0.0000
	Calculus of lower urinary tract. unspecified	N21.9	0	0.000	1	0.009	0	0.000	1	0.003	0.8776
	**Calculus of urinary tract in diseases classified elsewhere**	**N22**	0	0.000	0	0.000	0	0.000	0	0.000	0.0000
	Other inflammatory disorders of penis	N48.2	0	0.000	0	0.000	0	0.000	0	0.000	0.0000
	Ulcer of penis	N48.5	8	0.050	5	0.043	0	0.000	13	0.043	0.9951
	Induration penis plastica	N48.6	8	0.050	6	0.051	1	0.037	15	0.049	0.8140
	Ulceration of vagina	N76.5	11	0.069	4	0.034	2	0.074	17	0.056	0.3313
	Ulceration of vulva	N76.6	15	0.094	7	0.060	2	0.074	24	0.079	0.4306
**Total**			2046	12.849	1106	9.438	279	10.364	3431	11.311	0.0000

Supplementary Table 4: Cerebrovascular diseases

Organ System	Extraintestinal Manifestations and Associated Immune Disorders	ICD-10	Crohn's Disease		Ulcerative Colitis		IBD-Unclassified		IBD-Total (CD + UC + IBDU)		Comparison
			N = 15924		N = 11718		N = 2692		N = 30334		UC vs. CD
			n	%	n	%	n	%	n	%	p value ≤
**Cerebrovascular diseases**											
	**Nontraumatic subarachnoid hemorrhage**	**I60**	0	0.000	0	0.000	0	0.000	0	0.000	0.0000
	Nontraumatic subarachnoid hemorrhage from carotid siphon and bifurcation	I60.0	0	0.000	0	0.000	0	0.000	0	0.000	0.0000
	Nontraumatic subarachnoid hemorrhage from unspecified carotid siphon and bifurcation	I60.00	0	0.000	0	0.000	0	0.000	0	0.000	0.0000
	Nontraumatic subarachnoid hemorrhage from right carotid siphon and bifurcation	I60.01	0	0.000	0	0.000	0	0.000	0	0.000	0.0000
	Nontraumatic subarachnoid hemorrhage from left carotid siphon and bifurcation	I60.02	0	0.000	0	0.000	0	0.000	0	0.000	0.0000
	Nontraumatic subarachnoid hemorrhage from middle cerebral artery	I60.1	2	0.013	2	0.017	1	0.037	5	0.016	0.8430
	Nontraumatic subarachnoid hemorrhage from unspecified middle cerebral artery	I60.10	0	0.000	0	0.000	0	0.000	0	0.000	0.0000
	Nontraumatic subarachnoid hemorrhage from right middle cerebral artery	I60.11	0	0.000	0	0.000	0	0.000	0	0.000	0.0000
	Nontraumatic subarachnoid hemorrhage from left middle cerebral artery	I60.12	0	0.000	0	0.000	0	0.000	0	0.000	0.0000
	Nontraumatic subarachnoid hemorrhage from anterior communicating artery	I60.2	1	0.006	3	0.026	0	0.000	4	0.013	0.4157
	Nontraumatic subarachnoid hemorrhage from posterior communicating artery	I60.3	3	0.019	1	0.009	1	0.037	5	0.016	0.8430
	Nontraumatic subarachnoid hemorrhage from unspecified posterior communicating artery	I60.30	0	0.000	0	0.000	0	0.000	0	0.000	0.0000
	Nontraumatic subarachnoid hemorrhage from right posterior communicating artery	I60.31	0	0.000	0	0.000	0	0.000	0	0.000	0.0000
	Nontraumatic subarachnoid hemorrhage from left posterior communicating artery	I60.32	0	0.000	0	0.000	0	0.000	0	0.000	0.0000
	Nontraumatic subarachnoid hemorrhage from basilar artery	I60.4	1	0.006	0	0.000	0	0.000	1	0.003	0.8776
	Nontraumatic subarachnoid hemorrhage from vertebral artery	I60.5	0	0.000	0	0.000	1	0.037	1	0.003	0.0000
	Nontraumatic subarachnoid hemorrhage from unspecified vertebral artery	I60.50	0	0.000	0	0.000	0	0.000	0	0.000	0.0000
	Nontraumatic subarachnoid hemorrhage from right vertebral artery	I60.51	0	0.000	0	0.000	0	0.000	0	0.000	0.0000
	Nontraumatic subarachnoid hemorrhage from left vertebral artery	I60.52	0	0.000	0	0.000	0	0.000	0	0.000	0.0000
	Nontraumatic subarachnoid hemorrhage from other intracranial arteries	I60.6	2	0.013	0	0.000	1	0.037	3	0.010	0.6187

	Nontraumatic subarachnoid hemorrhage from unspecified intracranial artery	I60.7	1	0.006	3	0.026	0	0.000	4	0.013	0.4157
	Other nontraumatic subarachnoid hemorrhage	I60.8	5	0.031	4	0.034	2	0.074	11	0.036	0.8316
	Nontraumatic subarachnoid hemorrhage. unspecified	I60.9	60	0.377	37	0.316	7	0.260	104	0.343	0.4562
	**Nontraumatic intracerebral hemorrhage**	**I61**	0	0.000	0	0.000	0	0.000	0	0.000	0.0000
	Nontraumatic intracerebral hemorrhage in hemisphere. subcortical	I61.0	8	0.050	6	0.051	2	0.074	16	0.053	0.8140
	Nontraumatic intracerebral hemorrhage in hemisphere. cortical	I61.1	13	0.082	8	0.068	2	0.074	23	0.076	0.8589
	Nontraumatic intracerebral hemorrhage in hemisphere. unspecified	I61.2	4	0.025	0	0.000	0	0.000	4	0.013	0.2263
	Nontraumatic intracerebral hemorrhage in brain stem	I61.3	2	0.013	1	0.009	0	0.000	3	0.010	0.7897
	Nontraumatic intracerebral hemorrhage in cerebellum	I61.4	9	0.057	5	0.043	3	0.111	17	0.056	0.8140
	Nontraumatic intracerebral hemorrhage. intraventricular	I61.5	5	0.031	2	0.017	1	0.037	8	0.026	0.7207
	Nontraumatic intracerebral hemorrhage. multiple localized	I61.6	0	0.000	1	0.009	0	0.000	1	0.003	0.8776
	Other nontraumatic intracerebral hemorrhage	I61.8	6	0.038	7	0.060	0	0.000	13	0.043	0.5787
	Nontraumatic intracerebral hemorrhage. unspecified	I61.9	38	0.239	21	0.179	6	0.223	65	0.214	0.3544
	**Other and unspecified nontraumatic intracranial hemorrhage**	**I62**	0	0.000	0	0.000	0	0.000	0	0.000	0.0000
	Nontraumatic subdural hemorrhage	I62.0	27	0.170	15	0.128	9	0.334	51	0.168	0.4714
	Nontraumatic subdural hemorrhage. unspecified	I62.00	0	0.000	0	0.000	0	0.000	0	0.000	0.0000
	Nontraumatic acute subdural hemorrhage	I62.01	0	0.000	0	0.000	0	0.000	0	0.000	0.0000
	Nontraumatic subacute subdural hemorrhage	I62.02	0	0.000	0	0.000	0	0.000	0	0.000	0.0000
	Nontraumatic chronic subdural hemorrhage	I62.03	0	0.000	0	0.000	0	0.000	0	0.000	0.0000
	Nontraumatic extradural hemorrhage	I62.1	0	0.000	0	0.000	0	0.000	0	0.000	0.0000
	Nontraumatic intracranial hemorrhage. unspecified	I62.9	17	0.107	18	0.154	4	0.149	39	0.129	0.3621
	**Cerebral infarction**	**I63**	0	0.000	0	0.000	0	0.000	0	0.000	0.0000
	Cerebral infarction due to thrombosis of precerebral arteries	I63.0	9	0.057	3	0.026	1	0.037	13	0.043	0.3538
	Cerebral infarction due to thrombosis of unspecified precerebral artery	I63.00	0	0.000	0	0.000	0	0.000	0	0.000	0.0000
	Cerebral infarction due to thrombosis of vertebral artery	I63.01	0	0.000	0	0.000	0	0.000	0	0.000	0.0000
	Cerebral infarction due to thrombosis of right vertebral artery	I63.011	0	0.000	0	0.000	0	0.000	0	0.000	0.0000
	Cerebral infarction due to thrombosis of left vertebral artery	I63.012	0	0.000	0	0.000	0	0.000	0	0.000	0.0000
	Cerebral infarction due to thrombosis of bilateral vertebral arteries	I63.013	0	0.000	0	0.000	0	0.000	0	0.000	0.0000
	Cerebral infarction due to thrombosis of unspecified vertebral artery	I63.019	0	0.000	0	0.000	0	0.000	0	0.000	0.0000

	Cerebral infarction due to thrombosis of basilar artery	I63.02	0	0.000	0	0.000	0	0.000	0	0.000	0.0000
	Cerebral infarction due to thrombosis of carotid artery	I63.03	0	0.000	0	0.000	0	0.000	0	0.000	0.0000
	Cerebral infarction due to thrombosis of right carotid artery	I63.031	0	0.000	0	0.000	0	0.000	0	0.000	0.0000
	Cerebral infarction due to thrombosis of left carotid artery	I63.032	0	0.000	0	0.000	0	0.000	0	0.000	0.0000
	Cerebral infarction due to thrombosis of bilateral carotid arteries	I63.033	0	0.000	0	0.000	0	0.000	0	0.000	0.0000
	Cerebral infarction due to thrombosis of unspecified carotid artery	I63.039	0	0.000	0	0.000	0	0.000	0	0.000	0.0000
	Cerebral infarction due to thrombosis of other precerebral artery	I63.09	0	0.000	0	0.000	0	0.000	0	0.000	0.0000
	Cerebral infarction due to embolism of precerebral arteries	I63.1	3	0.019	2	0.017	0	0.000	5	0.016	0.7306
	Cerebral infarction due to embolism of unspecified precerebral artery	I63.10	0	0.000	0	0.000	0	0.000	0	0.000	0.0000
	Cerebral infarction due to embolism of vertebral artery	I63.11	0	0.000	0	0.000	0	0.000	0	0.000	0.0000
	Cerebral infarction due to embolism of right vertebral artery	I63.111	0	0.000	0	0.000	0	0.000	0	0.000	0.0000
	Cerebral infarction due to embolism of left vertebral artery	I63.112	0	0.000	0	0.000	0	0.000	0	0.000	0.0000
	Cerebral infarction due to embolism of bilateral vertebral arteries	I63.113	0	0.000	0	0.000	0	0.000	0	0.000	0.0000
	Cerebral infarction due to embolism of unspecified vertebral artery	I63.119	0	0.000	0	0.000	0	0.000	0	0.000	0.0000
	Cerebral infarction due to embolism of basilar artery	I63.12	0	0.000	0	0.000	0	0.000	0	0.000	0.0000
	Cerebral infarction due to embolism of carotid artery	I63.13	0	0.000	0	0.000	0	0.000	0	0.000	0.0000
	Cerebral infarction due to embolism of right carotid artery	I63.131	0	0.000	0	0.000	0	0.000	0	0.000	0.0000
	Cerebral infarction due to embolism of left carotid artery	I63.132	0	0.000	0	0.000	0	0.000	0	0.000	0.0000
	Cerebral infarction due to embolism of bilateral carotid arteries	I63.133	0	0.000	0	0.000	0	0.000	0	0.000	0.0000
	Cerebral infarction due to embolism of unspecified carotid artery	I63.139	0	0.000	0	0.000	0	0.000	0	0.000	0.0000
	Cerebral infarction due to embolism of other precerebral artery	I63.19	0	0.000	0	0.000	0	0.000	0	0.000	0.0000
	Cerebral infarction due to unspecified occlusion or stenosis of precerebral arteries	I63.2	12	0.075	9	0.077	3	0.111	24	0.079	0.8589
	Cerebral infarction due to unspecified occlusion or stenosis of unspecified precerebral arteries	I63.20	0	0.000	0	0.000	0	0.000	0	0.000	0.0000
	Cerebral infarction due to unspecified occlusion or stenosis of vertebral arteries	I63.21	0	0.000	0	0.000	0	0.000	0	0.000	0.0000
	Cerebral infarction due to unspecified occlusion or stenosis of right vertebral artery	I63.211	0	0.000	0	0.000	0	0.000	0	0.000	0.0000
	Cerebral infarction due to unspecified occlusion or stenosis of left vertebral artery	I63.212	0	0.000	0	0.000	0	0.000	0	0.000	0.0000
	Cerebral infarction due to unspecified occlusion or stenosis of bilateral vertebral arteries	I63.213	0	0.000	0	0.000	0	0.000	0	0.000	0.0000
	Cerebral infarction due to unspecified occlusion or stenosis of unspecified vertebral artery	I63.219	0	0.000	0	0.000	0	0.000	0	0.000	0.0000
	Cerebral infarction due to unspecified occlusion or stenosis of basilar artery	I63.22	0	0.000	0	0.000	0	0.000	0	0.000	0.0000

	Cerebral infarction due to unspecified occlusion or stenosis of carotid arteries	I63.23	0	0.000	0	0.000	0	0.000	0	0.000	0.0000
	Cerebral infarction due to unspecified occlusion or stenosis of right carotid arteries	I63.231	0	0.000	0	0.000	0	0.000	0	0.000	0.0000
	Cerebral infarction due to unspecified occlusion or stenosis of left carotid arteries	I63.232	0	0.000	0	0.000	0	0.000	0	0.000	0.0000
	Cerebral infarction due to unspecified occlusion or stenosis of bilateral carotid arteries	I63.233	0	0.000	0	0.000	0	0.000	0	0.000	0.0000
	Cerebral infarction due to unspecified occlusion or stenosis of unspecified carotid artery	I63.239	0	0.000	0	0.000	0	0.000	0	0.000	0.0000
	Cerebral infarction due to unspecified occlusion or stenosis of other precerebral arteries	I63.29	0	0.000	0	0.000	0	0.000	0	0.000	0.0000
	Cerebral infarction due to thrombosis of cerebral arteries	I63.3	35	0.220	22	0.188	6	0.223	63	0.208	0.6554
	Cerebral infarction due to thrombosis of unspecified cerebral artery	I63.30	0	0.000	0	0.000	0	0.000	0	0.000	0.0000
	Cerebral infarction due to thrombosis of middle cerebral artery	I63.31	0	0.000	0	0.000	0	0.000	0	0.000	0.0000
	Cerebral infarction due to thrombosis of right middle cerebral artery	I63.311	0	0.000	0	0.000	0	0.000	0	0.000	0.0000
	Cerebral infarction due to thrombosis of left middle cerebral artery	I63.312	0	0.000	0	0.000	0	0.000	0	0.000	0.0000
	Cerebral infarction due to thrombosis of bilateral middle cerebral arteries	I63.313	0	0.000	0	0.000	0	0.000	0	0.000	0.0000
	Cerebral infarction due to thrombosis of unspecified middle cerebral artery	I63.319	0	0.000	0	0.000	0	0.000	0	0.000	0.0000
	Cerebral infarction due to thrombosis of anterior cerebral artery	I63.32	0	0.000	0	0.000	0	0.000	0	0.000	0.0000
	Cerebral infarction due to thrombosis of right anterior cerebral artery	I63.321	0	0.000	0	0.000	0	0.000	0	0.000	0.0000
	Cerebral infarction due to thrombosis of left anterior cerebral artery	I63.322	0	0.000	0	0.000	0	0.000	0	0.000	0.0000
	Cerebral infarction due to thrombosis of bilateral anterior cerebral arteries	I63.323	0	0.000	0	0.000	0	0.000	0	0.000	0.0000
	Cerebral infarction due to thrombosis of unspecified anterior cerebral artery	I63.329	0	0.000	0	0.000	0	0.000	0	0.000	0.0000
	Cerebral infarction due to thrombosis of posterior cerebral artery	I63.33	0	0.000	0	0.000	0	0.000	0	0.000	0.0000
	Cerebral infarction due to thrombosis of right posterior cerebral artery	I63.331	0	0.000	0	0.000	0	0.000	0	0.000	0.0000
	Cerebral infarction due to thrombosis of left posterior cerebral artery	I63.332	0	0.000	0	0.000	0	0.000	0	0.000	0.0000
	Cerebral infarction due to thrombosis of bilateral posterior cerebral arteries	I63.333	0	0.000	0	0.000	0	0.000	0	0.000	0.0000
	Cerebral infarction due to thrombosis of unspecified posterior cerebral artery	I63.339	0	0.000	0	0.000	0	0.000	0	0.000	0.0000
	Cerebral infarction due to thrombosis of cerebellar artery	I63.34	0	0.000	0	0.000	0	0.000	0	0.000	0.0000
	Cerebral infarction due to thrombosis of right cerebellar artery	I63.341	0	0.000	0	0.000	0	0.000	0	0.000	0.0000
	Cerebral infarction due to thrombosis of left cerebellar artery	I63.342	0	0.000	0	0.000	0	0.000	0	0.000	0.0000
	Cerebral infarction due to thrombosis of bilateral cerebellar arteries	I63.343	0	0.000	0	0.000	0	0.000	0	0.000	0.0000
	Cerebral infarction due to thrombosis of unspecified cerebellar artery	I63.349	0	0.000	0	0.000	0	0.000	0	0.000	0.0000
	Cerebral infarction due to thrombosis of other cerebral artery	I63.39	0	0.000	0	0.000	0	0.000	0	0.000	0.0000

	Cerebral infarction due to embolism of cerebral arteries	I63.4	40	0.251	43	0.367	8	0.297	91	0.300	0.1037
	Cerebral infarction due to embolism of unspecified cerebral artery	I63.40	0	0.000	0	0.000	0	0.000	0	0.000	0.0000
	Cerebral infarction due to embolism of middle cerebral artery	I63.41	0	0.000	0	0.000	0	0.000	0	0.000	0.0000
	Cerebral infarction due to embolism of right middle cerebral artery	I63.411	0	0.000	0	0.000	0	0.000	0	0.000	0.0000
	Cerebral infarction due to embolism of left middle cerebral artery	I63.412	0	0.000	0	0.000	0	0.000	0	0.000	0.0000
	Cerebral infarction due to embolism of bilateral middle cerebral arteries	I63.413	0	0.000	0	0.000	0	0.000	0	0.000	0.0000
	Cerebral infarction due to embolism of unspecified middle cerebral artery	I63.419	0	0.000	0	0.000	0	0.000	0	0.000	0.0000
	Cerebral infarction due to embolism of anterior cerebral artery	I63.42	0	0.000	0	0.000	0	0.000	0	0.000	0.0000
	Cerebral infarction due to embolism of right anterior cerebral artery	I63.421	0	0.000	0	0.000	0	0.000	0	0.000	0.0000
	Cerebral infarction due to embolism of left anterior cerebral artery	I63.422	0	0.000	0	0.000	0	0.000	0	0.000	0.0000
	Cerebral infarction due to embolism of bilateral anterior cerebral arteries	I63.423	0	0.000	0	0.000	0	0.000	0	0.000	0.0000
	Cerebral infarction due to embolism of unspecified anterior cerebral artery	I63.429	0	0.000	0	0.000	0	0.000	0	0.000	0.0000
	Cerebral infarction due to embolism of posterior cerebral artery	I63.43	0	0.000	0	0.000	0	0.000	0	0.000	0.0000
	Cerebral infarction due to embolism of right posterior cerebral artery	I63.431	0	0.000	0	0.000	0	0.000	0	0.000	0.0000
	Cerebral infarction due to embolism of left posterior cerebral artery	I63.432	0	0.000	0	0.000	0	0.000	0	0.000	0.0000
	Cerebral infarction due to embolism of bilateral posterior cerebral arteries	I63.433	0	0.000	0	0.000	0	0.000	0	0.000	0.0000
	Cerebral infarction due to embolism of unspecified posterior cerebral artery	I63.439	0	0.000	0	0.000	0	0.000	0	0.000	0.0000
	Cerebral infarction due to embolism of cerebellar artery	I63.44	0	0.000	0	0.000	0	0.000	0	0.000	0.0000
	Cerebral infarction due to embolism of right cerebellar artery	I63.441	0	0.000	0	0.000	0	0.000	0	0.000	0.0000
	Cerebral infarction due to embolism of left cerebellar artery	I63.442	0	0.000	0	0.000	0	0.000	0	0.000	0.0000
	Cerebral infarction due to embolism of bilateral cerebellar arteries	I63.443	0	0.000	0	0.000	0	0.000	0	0.000	0.0000
	Cerebral infarction due to embolism of unspecified cerebellar artery	I63.449	0	0.000	0	0.000	0	0.000	0	0.000	0.0000
	Cerebral infarction due to embolism of other cerebral artery	I63.49	0	0.000	0	0.000	0	0.000	0	0.000	0.0000
	Cerebral infarction due to unspecified occlusion or stenosis of cerebral arteries	I63.5	57	0.358	55	0.469	18	0.669	130	0.429	0.1786
	Cerebral infarction due to unspecified occlusion or stenosis of unspecified cerebral artery	I63.50	0	0.000	0	0.000	0	0.000	0	0.000	0.0000
	Cerebral infarction due to unspecified occlusion or stenosis of middle cerebral artery	I63.51	0	0.000	0	0.000	0	0.000	0	0.000	0.0000
	Cerebral infarction due to unspecified occlusion or stenosis of right middle cerebral artery	I63.511	0	0.000	0	0.000	0	0.000	0	0.000	0.0000
	Cerebral infarction due to unspecified occlusion or stenosis of left middle cerebral artery	I63.512	0	0.000	0	0.000	0	0.000	0	0.000	0.0000
	Cerebral infarction due to unspecified occlusion or stenosis of bilateral middle cerebral arteries	I63.513	0	0.000	0	0.000	0	0.000	0	0.000	0.0000

	Cerebral infarction due to unspecified occlusion or stenosis of unspecified middle cerebral artery	I63.519	0	0.000	0	0.000	0	0.000	0	0.000	0.0000
	Cerebral infarction due to unspecified occlusion or stenosis of anterior cerebral artery	I63.52	0	0.000	0	0.000	0	0.000	0	0.000	0.0000
	Cerebral infarction due to unspecified occlusion or stenosis of right anterior cerebral artery	I63.521	0	0.000	0	0.000	0	0.000	0	0.000	0.0000
	Cerebral infarction due to unspecified occlusion or stenosis of left anterior cerebral artery	I63.522	0	0.000	0	0.000	0	0.000	0	0.000	0.0000
	Cerebral infarction due to unspecified occlusion or stenosis of bilateral anterior cerebral arteries	I63.523	0	0.000	0	0.000	0	0.000	0	0.000	0.0000
	Cerebral infarction due to unspecified occlusion or stenosis of unspecified anterior cerebral artery	I63.529	0	0.000	0	0.000	0	0.000	0	0.000	0.0000
	Cerebral infarction due to unspecified occlusion or stenosis of posterior cerebral artery	I63.53	0	0.000	0	0.000	0	0.000	0	0.000	0.0000
	Cerebral infarction due to unspecified occlusion or stenosis of right posterior cerebral artery	I63.531	0	0.000	0	0.000	0	0.000	0	0.000	0.0000
	Cerebral infarction due to unspecified occlusion or stenosis of left posterior cerebral artery	I63.532	0	0.000	0	0.000	0	0.000	0	0.000	0.0000
	Cerebral infarction due to unspecified occlusion or stenosis of bilateral posterior cerebral arteries	I63.533	0	0.000	0	0.000	0	0.000	0	0.000	0.0000
	Cerebral infarction due to unspecified occlusion or stenosis of unspecified posterior cerebral artery	I63.539	0	0.000	0	0.000	0	0.000	0	0.000	0.0000
	Cerebral infarction due to unspecified occlusion or stenosis of cerebellar artery	I63.54	0	0.000	0	0.000	0	0.000	0	0.000	0.0000
	Cerebral infarction due to unspecified occlusion or stenosis of right cerebellar artery	I63.541	0	0.000	0	0.000	0	0.000	0	0.000	0.0000
	Cerebral infarction due to unspecified occlusion or stenosis of left cerebellar artery	I63.542	0	0.000	0	0.000	0	0.000	0	0.000	0.0000
	Cerebral infarction due to unspecified occlusion or stenosis of bilateral cerebellar arteries	I63.543	0	0.000	0	0.000	0	0.000	0	0.000	0.0000
	Cerebral infarction due to unspecified occlusion or stenosis of unspecified cerebellar artery	I63.549	0	0.000	0	0.000	0	0.000	0	0.000	0.0000
	Cerebral infarction due to unspecified occlusion or stenosis of other cerebral artery	I63.59	0	0.000	0	0.000	0	0.000	0	0.000	0.0000
	Cerebral infarction due to cerebral venous thrombosis. nonpyogenic	I63.6	8	0.050	7	0.060	2	0.074	17	0.056	0.9412
	Other cerebral infarction	I63.8	24	0.151	13	0.111	1	0.037	38	0.125	0.4670
	Other cerebral infarction due to occlusion or stenosis of small artery	I63.81	0	0.000	0	0.000	0	0.000	0	0.000	0.0000
	Other cerebral infarction	I63.89	0	0.000	0	0.000	0	0.000	0	0.000	0.0000
	Cerebral infarction. unspecified	I63.9	137	0.860	107	0.913	29	1.077	273	0.900	0.6902
	**Stroke. not specified as haemorrhage or infarction**	**I64**	351	2.204	285	2.432	79	2.935	715	2.357	0.2269
	**Other cerebrovascular diseases**	**I67**	0	0.000	0	0.000	0	0.000	0	0.000	0.0000
	Dissection of cerebral arteries. nonruptured	I67.0	10	0.063	1	0.009	2	0.074	13	0.043	0.0536
	Cerebral aneurysm. nonruptured	I67.1	44	0.276	12	0.102	5	0.186	61	0.201	0.0023
	Cerebral atherosclerosis	I67.2	7	0.044	6	0.051	3	0.111	16	0.053	0.9951
	Progressive vascular leukoencephalopathy	I67.3	1	0.006	6	0.051	1	0.037	8	0.026	0.0527
	Hypertensive encephalopathy	I67.4	2	0.013	2	0.017	1	0.037	5	0.016	0.8430

	Moyamoya disease	I67.5	1	0.006	0	0.000	0	0.000	1	0.003	0.8776
	Nonpyogenic thrombosis of intracranial venous system	I67.6	6	0.038	1	0.009	0	0.000	7	0.023	0.2617
	Cerebral arteritis. not elsewhere classified	I67.7	4	0.025	0	0.000	0	0.000	4	0.013	0.2263
	Other specified cerebrovascular diseases	I67.8	15	0.094	16	0.137	2	0.074	33	0.109	0.3911
	Acute cerebrovascular insufficiency	I67.81	0	0.000	0	0.000	0	0.000	0	0.000	0.0000
	Cerebral ischemia	I67.82	0	0.000	0	0.000	0	0.000	0	0.000	0.0000
	Posterior reversible encephalopathy syndrome	I67.83	0	0.000	0	0.000	0	0.000	0	0.000	0.0000
	Cerebral vasospasm and vasoconstriction	I67.84	0	0.000	0	0.000	0	0.000	0	0.000	0.0000
	Reversible cerebrovascular vasoconstriction syndrome	I67.841	0	0.000	0	0.000	0	0.000	0	0.000	0.0000
	Other cerebrovascular vasospasm and vasoconstriction	I67.848	0	0.000	0	0.000	0	0.000	0	0.000	0.0000
	Hereditary cerebrovascular diseases	I67.85	0	0.000	0	0.000	0	0.000	0	0.000	0.0000
	Cerebral autosomal dominant arteriopathy with subcortical infarcts and leukoencephalopathy	I67.850	0	0.000	0	0.000	0	0.000	0	0.000	0.0000
	Other hereditary cerebrovascular disease	I67.858	0	0.000	0	0.000	0	0.000	0	0.000	0.0000
	Other cerebrovascular disease	I67.89	0	0.000	0	0.000	0	0.000	0	0.000	0.0000
	Cerebrovascular disease. unspecified	I67.9	40	0.251	36	0.307	12	0.446	88	0.290	0.4455
	**Phlebitis and thrombophlebitis**	**I80**	0	0.000	0	0.000	0	0.000	0	0.000	0.0000
	Phlebitis and thrombophlebitis of superficial vessels of lower extremities	I80.0	109	0.685	90	0.768	16	0.594	215	0.709	0.4593
	Phlebitis and thrombophlebitis of superficial vessels of unspecified lower extremity	I80.00	0	0.000	0	0.000	0	0.000	0	0.000	0.0000
	Phlebitis and thrombophlebitis of superficial vessels of right lower extremity	I80.01	0	0.000	0	0.000	0	0.000	0	0.000	0.0000
	Phlebitis and thrombophlebitis of superficial vessels of left lower extremity	I80.02	0	0.000	0	0.000	0	0.000	0	0.000	0.0000
	Phlebitis and thrombophlebitis of superficial vessels of lower extremities. bilateral	I80.03	0	0.000	0	0.000	0	0.000	0	0.000	0.0000
	Phlebitis and thrombophlebitis of femoral vein	I80.1	14	0.088	7	0.060	3	0.111	24	0.079	0.5356
	Phlebitis and thrombophlebitis of unspecified femoral vein	I80.10	0	0.000	0	0.000	0	0.000	0	0.000	0.0000
	Phlebitis and thrombophlebitis of right femoral vein	I80.11	0	0.000	0	0.000	0	0.000	0	0.000	0.0000
	Phlebitis and thrombophlebitis of left femoral vein	I80.12	0	0.000	0	0.000	0	0.000	0	0.000	0.0000
	Phlebitis and thrombophlebitis of femoral vein. bilateral	I80.13	0	0.000	0	0.000	0	0.000	0	0.000	0.0000
	Phlebitis and thrombophlebitis of other and unspecified deep vessels of lower extremities	I80.2	746	4.685	552	4.711	130	4.829	1428	4.708	0.9426
	Phlebitis and thrombophlebitis of unspecified deep vessels of lower extremities	I80.20	0	0.000	0	0.000	0	0.000	0	0.000	0.0000
	Phlebitis and thrombophlebitis of unspecified deep vessels of right lower extremity	I80.201	0	0.000	0	0.000	0	0.000	0	0.000	0.0000

	Phlebitis and thrombophlebitis of unspecified deep vessels of left lower extremity	I80.202	0	0.000	0	0.000	0	0.000	0	0.000	0.0000
	Phlebitis and thrombophlebitis of unspecified deep vessels of lower extremities. bilateral	I80.203	0	0.000	0	0.000	0	0.000	0	0.000	0.0000
	Phlebitis and thrombophlebitis of unspecified deep vessels of unspecified lower extremity	I80.209	0	0.000	0	0.000	0	0.000	0	0.000	0.0000
	Phlebitis and thrombophlebitis of iliac vein	I80.21	0	0.000	0	0.000	0	0.000	0	0.000	0.0000
	Phlebitis and thrombophlebitis of right iliac vein	I80.211	0	0.000	0	0.000	0	0.000	0	0.000	0.0000
	Phlebitis and thrombophlebitis of left iliac vein	I80.212	0	0.000	0	0.000	0	0.000	0	0.000	0.0000
	Phlebitis and thrombophlebitis of iliac vein. bilateral	I80.213	0	0.000	0	0.000	0	0.000	0	0.000	0.0000
	Phlebitis and thrombophlebitis of unspecified iliac vein	I80.219	0	0.000	0	0.000	0	0.000	0	0.000	0.0000
	Phlebitis and thrombophlebitis of popliteal vein	I80.22	0	0.000	0	0.000	0	0.000	0	0.000	0.0000
	Phlebitis and thrombophlebitis of right popliteal vein	I80.221	0	0.000	0	0.000	0	0.000	0	0.000	0.0000
	Phlebitis and thrombophlebitis of left popliteal vein	I80.222	0	0.000	0	0.000	0	0.000	0	0.000	0.0000
	Phlebitis and thrombophlebitis of popliteal vein. bilateral	I80.223	0	0.000	0	0.000	0	0.000	0	0.000	0.0000
	Phlebitis and thrombophlebitis of unspecified popliteal vein	I80.229	0	0.000	0	0.000	0	0.000	0	0.000	0.0000
	Phlebitis and thrombophlebitis of tibial vein	I80.23	0	0.000	0	0.000	0	0.000	0	0.000	0.0000
	Phlebitis and thrombophlebitis of right tibial vein	I80.231	0	0.000	0	0.000	0	0.000	0	0.000	0.0000
	Phlebitis and thrombophlebitis of left tibial vein	I80.232	0	0.000	0	0.000	0	0.000	0	0.000	0.0000
	Phlebitis and thrombophlebitis of tibial vein. bilateral	I80.233	0	0.000	0	0.000	0	0.000	0	0.000	0.0000
	Phlebitis and thrombophlebitis of unspecified tibial vein	I80.239	0	0.000	0	0.000	0	0.000	0	0.000	0.0000
	Phlebitis and thrombophlebitis of peroneal vein	I80.24	0	0.000	0	0.000	0	0.000	0	0.000	0.0000
	Phlebitis and thrombophlebitis of right peroneal vein	I80.241	0	0.000	0	0.000	0	0.000	0	0.000	0.0000
	Phlebitis and thrombophlebitis of left peroneal vein	I80.242	0	0.000	0	0.000	0	0.000	0	0.000	0.0000
	Phlebitis and thrombophlebitis of peroneal vein. bilateral	I80.243	0	0.000	0	0.000	0	0.000	0	0.000	0.0000
	Phlebitis and thrombophlebitis of unspecified peroneal vein	I80.249	0	0.000	0	0.000	0	0.000	0	0.000	0.0000
	Phlebitis and thrombophlebitis of calf muscular vein	I80.25	0	0.000	0	0.000	0	0.000	0	0.000	0.0000
	Phlebitis and thrombophlebitis of right calf muscular vein	I80.251	0	0.000	0	0.000	0	0.000	0	0.000	0.0000
	Phlebitis and thrombophlebitis of left calf muscular vein	I80.252	0	0.000	0	0.000	0	0.000	0	0.000	0.0000
	Phlebitis and thrombophlebitis of calf muscular vein. bilateral	I80.253	0	0.000	0	0.000	0	0.000	0	0.000	0.0000
	Phlebitis and thrombophlebitis of unspecified calf muscular vein	I80.259	0	0.000	0	0.000	0	0.000	0	0.000	0.0000
	Phlebitis and thrombophlebitis of other deep vessels of lower extremities	I80.29	0	0.000	0	0.000	0	0.000	0	0.000	0.0000

	Phlebitis and thrombophlebitis of other deep vessels of right lower extremity	I80.291	0	0.000	0	0.000	0	0.000	0	0.000	0.0000
	Phlebitis and thrombophlebitis of other deep vessels of left lower extremity	I80.292	0	0.000	0	0.000	0	0.000	0	0.000	0.0000
	Phlebitis and thrombophlebitis of other deep vessels of lower extremity. bilateral	I80.293	0	0.000	0	0.000	0	0.000	0	0.000	0.0000
	Phlebitis and thrombophlebitis of other deep vessels of unspecified lower extremity	I80.299	0	0.000	0	0.000	0	0.000	0	0.000	0.0000
	Phlebitis and thrombophlebitis of lower extremities. unspecified	I80.3	54	0.339	45	0.384	7	0.260	106	0.349	0.6060
	Phlebitis and thrombophlebitis of other sites	I80.8	140	0.879	80	0.683	18	0.669	238	0.785	0.0804
	Phlebitis and thrombophlebitis of unspecified site	I80.9	58	0.364	30	0.256	8	0.297	96	0.316	0.1415
	**Portal vein thrombosis**	**I81**	58	0.364	44	0.375	11	0.409	113	0.373	0.9584
	**Other venous embolism and thrombosis**	**I82**	0	0.000	0	0.000	0	0.000	0	0.000	0.0000
	Budd-Chiari syndrome	I82.0	5	0.031	5	0.043	1	0.037	11	0.036	0.8674
	Thrombophlebitis migrans	I82.1	1	0.006	2	0.017	0	0.000	3	0.010	0.7897
	Embolism and thrombosis of vena cava and other thoracic veins	I82.2	23	0.144	5	0.043	1	0.037	29	0.096	0.0148
	Embolism and thrombosis of superior vena cava	I82.21	0	0.000	0	0.000	0	0.000	0	0.000	0.0000
	Acute embolism and thrombosis of superior vena cava	I82.210	0	0.000	0	0.000	0	0.000	0	0.000	0.0000
	Chronic embolism and thrombosis of superior vena cava	I82.211	0	0.000	0	0.000	0	0.000	0	0.000	0.0000
	Embolism and thrombosis of inferior vena cava	I82.22	0	0.000	0	0.000	0	0.000	0	0.000	0.0000
	Acute embolism and thrombosis of inferior vena cava	I82.220	0	0.000	0	0.000	0	0.000	0	0.000	0.0000
	Chronic embolism and thrombosis of inferior vena cava	I82.221	0	0.000	0	0.000	0	0.000	0	0.000	0.0000
	Embolism and thrombosis of other thoracic veins	I82.29	0	0.000	0	0.000	0	0.000	0	0.000	0.0000
	Acute embolism and thrombosis of other thoracic veins	I82.290	0	0.000	0	0.000	0	0.000	0	0.000	0.0000
	Chronic embolism and thrombosis of other thoracic veins	I82.291	0	0.000	0	0.000	0	0.000	0	0.000	0.0000
	Embolism and thrombosis of renal vein	I82.3	4	0.025	0	0.000	1	0.037	5	0.016	0.2263
	Acute embolism and thrombosis of deep veins of lower extremity	I82.4	0	0.000	0	0.000	0	0.000	0	0.000	0.0000
	Acute embolism and thrombosis of unspecified deep veins of lower extremity	I82.40	0	0.000	0	0.000	0	0.000	0	0.000	0.0000
	Acute embolism and thrombosis of unspecified deep veins of right lower extremity	I82.401	0	0.000	0	0.000	0	0.000	0	0.000	0.0000
	Acute embolism and thrombosis of unspecified deep veins of left lower extremity	I82.402	0	0.000	0	0.000	0	0.000	0	0.000	0.0000
	Acute embolism and thrombosis of unspecified deep veins of lower extremity. bilateral	I82.403	0	0.000	0	0.000	0	0.000	0	0.000	0.0000
	Acute embolism and thrombosis of unspecified deep veins of unspecified lower extremity	I82.409	0	0.000	0	0.000	0	0.000	0	0.000	0.0000
	Acute embolism and thrombosis of femoral vein	I82.41	0	0.000	0	0.000	0	0.000	0	0.000	0.0000

	Acute embolism and thrombosis of right femoral vein	I82.411	0	0.000	0	0.000	0	0.000	0	0.000	0.0000
	Acute embolism and thrombosis of left femoral vein	I82.412	0	0.000	0	0.000	0	0.000	0	0.000	0.0000
	Acute embolism and thrombosis of femoral vein. bilateral	I82.413	0	0.000	0	0.000	0	0.000	0	0.000	0.0000
	Acute embolism and thrombosis of unspecified femoral vein	I82.419	0	0.000	0	0.000	0	0.000	0	0.000	0.0000
	Acute embolism and thrombosis of iliac vein	I82.42	0	0.000	0	0.000	0	0.000	0	0.000	0.0000
	Acute embolism and thrombosis of right iliac vein	I82.421	0	0.000	0	0.000	0	0.000	0	0.000	0.0000
	Acute embolism and thrombosis of left iliac vein	I82.422	0	0.000	0	0.000	0	0.000	0	0.000	0.0000
	Acute embolism and thrombosis of iliac vein. bilateral	I82.423	0	0.000	0	0.000	0	0.000	0	0.000	0.0000
	Acute embolism and thrombosis of unspecified iliac vein	I82.429	0	0.000	0	0.000	0	0.000	0	0.000	0.0000
	Acute embolism and thrombosis of popliteal vein	I82.43	0	0.000	0	0.000	0	0.000	0	0.000	0.0000
	Acute embolism and thrombosis of right popliteal vein	I82.431	0	0.000	0	0.000	0	0.000	0	0.000	0.0000
	Acute embolism and thrombosis of left popliteal vein	I82.432	0	0.000	0	0.000	0	0.000	0	0.000	0.0000
	Acute embolism and thrombosis of popliteal vein. bilateral	I82.433	0	0.000	0	0.000	0	0.000	0	0.000	0.0000
	Acute embolism and thrombosis of unspecified popliteal vein	I82.439	0	0.000	0	0.000	0	0.000	0	0.000	0.0000
	Acute embolism and thrombosis of tibial vein	I82.44	0	0.000	0	0.000	0	0.000	0	0.000	0.0000
	Acute embolism and thrombosis of right tibial vein	I82.441	0	0.000	0	0.000	0	0.000	0	0.000	0.0000
	Acute embolism and thrombosis of left tibial vein	I82.442	0	0.000	0	0.000	0	0.000	0	0.000	0.0000
	Acute embolism and thrombosis of tibial vein. bilateral	I82.443	0	0.000	0	0.000	0	0.000	0	0.000	0.0000
	Acute embolism and thrombosis of unspecified tibial vein	I82.449	0	0.000	0	0.000	0	0.000	0	0.000	0.0000
	Acute embolism and thrombosis of peroneal vein	I82.45	0	0.000	0	0.000	0	0.000	0	0.000	0.0000
	Acute embolism and thrombosis of right peroneal vein	I82.451	0	0.000	0	0.000	0	0.000	0	0.000	0.0000
	Acute embolism and thrombosis of left peroneal vein	I82.452	0	0.000	0	0.000	0	0.000	0	0.000	0.0000
	Acute embolism and thrombosis of peroneal vein. bilateral	I82.453	0	0.000	0	0.000	0	0.000	0	0.000	0.0000
	Acute embolism and thrombosis of unspecified peroneal vein	I82.459	0	0.000	0	0.000	0	0.000	0	0.000	0.0000
	Acute embolism and thrombosis of calf muscular vein	I82.46	0	0.000	0	0.000	0	0.000	0	0.000	0.0000
	Acute embolism and thrombosis of right calf muscular vein	I82.461	0	0.000	0	0.000	0	0.000	0	0.000	0.0000
	Acute embolism and thrombosis of left calf muscular vein	I82.462	0	0.000	0	0.000	0	0.000	0	0.000	0.0000
	Acute embolism and thrombosis of calf muscular vein. bilateral	I82.463	0	0.000	0	0.000	0	0.000	0	0.000	0.0000
	Acute embolism and thrombosis of unspecified calf muscular vein	I82.469	0	0.000	0	0.000	0	0.000	0	0.000	0.0000

	Acute embolism and thrombosis of other specified deep vein of lower extremity	I82.49	0	0.000	0	0.000	0	0.000	0	0.000	0.0000
	Acute embolism and thrombosis of other specified deep vein of right lower extremity	I82.491	0	0.000	0	0.000	0	0.000	0	0.000	0.0000
	Acute embolism and thrombosis of other specified deep vein of left lower extremity	I82.492	0	0.000	0	0.000	0	0.000	0	0.000	0.0000
	Acute embolism and thrombosis of other specified deep vein of lower extremity. bilateral	I82.493	0	0.000	0	0.000	0	0.000	0	0.000	0.0000
	Acute embolism and thrombosis of other specified deep vein of unspecified lower extremity	I82.499	0	0.000	0	0.000	0	0.000	0	0.000	0.0000
	Acute embolism and thrombosis of unspecified deep veins of proximal lower extremity	I82.4Y	0	0.000	0	0.000	0	0.000	0	0.000	0.0000
	Acute embolism and thrombosis of unspecified deep veins of right proximal lower extremity	I82.4Y1	0	0.000	0	0.000	0	0.000	0	0.000	0.0000
	Acute embolism and thrombosis of unspecified deep veins of left proximal lower extremity	I82.4Y2	0	0.000	0	0.000	0	0.000	0	0.000	0.0000
	Acute embolism and thrombosis of unspecified deep veins of proximal lower extremity. bilateral	I82.4Y3	0	0.000	0	0.000	0	0.000	0	0.000	0.0000
	Acute embolism and thrombosis of unspecified deep veins of unspecified proximal lower extremity	I82.4Y9	0	0.000	0	0.000	0	0.000	0	0.000	0.0000
	Acute embolism and thrombosis of unspecified deep veins of distal lower extremity	I82.4Z	0	0.000	0	0.000	0	0.000	0	0.000	0.0000
	Acute embolism and thrombosis of unspecified deep veins of right distal lower extremity	I82.4Z1	0	0.000	0	0.000	0	0.000	0	0.000	0.0000
	Acute embolism and thrombosis of unspecified deep veins of left distal lower extremity	I82.4Z2	0	0.000	0	0.000	0	0.000	0	0.000	0.0000
	Acute embolism and thrombosis of unspecified deep veins of distal lower extremity. bilateral	I82.4Z3	0	0.000	0	0.000	0	0.000	0	0.000	0.0000
	Acute embolism and thrombosis of unspecified deep veins of unspecified distal lower extremity	I82.4Z9	0	0.000	0	0.000	0	0.000	0	0.000	0.0000
	Chronic embolism and thrombosis of deep veins of lower extremity	I82.5	0	0.000	0	0.000	0	0.000	0	0.000	0.0000
	Chronic embolism and thrombosis of unspecified deep veins of lower extremity	I82.50	0	0.000	0	0.000	0	0.000	0	0.000	0.0000
	Chronic embolism and thrombosis of unspecified deep veins of right lower extremity	I82.501	0	0.000	0	0.000	0	0.000	0	0.000	0.0000
	Chronic embolism and thrombosis of unspecified deep veins of left lower extremity	I82.502	0	0.000	0	0.000	0	0.000	0	0.000	0.0000
	Chronic embolism and thrombosis of unspecified deep veins of lower extremity. bilateral	I82.503	0	0.000	0	0.000	0	0.000	0	0.000	0.0000
	Chronic embolism and thrombosis of unspecified deep veins of unspecified lower extremity	I82.509	0	0.000	0	0.000	0	0.000	0	0.000	0.0000
	Chronic embolism and thrombosis of femoral vein	I82.51	0	0.000	0	0.000	0	0.000	0	0.000	0.0000
	Chronic embolism and thrombosis of right femoral vein	I82.511	0	0.000	0	0.000	0	0.000	0	0.000	0.0000
	Chronic embolism and thrombosis of left femoral vein	I82.512	0	0.000	0	0.000	0	0.000	0	0.000	0.0000
	Chronic embolism and thrombosis of femoral vein. bilateral	I82.513	0	0.000	0	0.000	0	0.000	0	0.000	0.0000
	Chronic embolism and thrombosis of unspecified femoral vein	I82.519	0	0.000	0	0.000	0	0.000	0	0.000	0.0000
	Chronic embolism and thrombosis of iliac vein	I82.52	0	0.000	0	0.000	0	0.000	0	0.000	0.0000
	Chronic embolism and thrombosis of right iliac vein	I82.521	0	0.000	0	0.000	0	0.000	0	0.000	0.0000
	Chronic embolism and thrombosis of left iliac vein	I82.522	0	0.000	0	0.000	0	0.000	0	0.000	0.0000

	Chronic embolism and thrombosis of iliac vein. bilateral	I82.523	0	0.000	0	0.000	0	0.000	0	0.000	0.0000
	Chronic embolism and thrombosis of unspecified iliac vein	I82.529	0	0.000	0	0.000	0	0.000	0	0.000	0.0000
	Chronic embolism and thrombosis of popliteal vein	I82.53	0	0.000	0	0.000	0	0.000	0	0.000	0.0000
	Chronic embolism and thrombosis of right popliteal vein	I82.531	0	0.000	0	0.000	0	0.000	0	0.000	0.0000
	Chronic embolism and thrombosis of left popliteal vein	I82.532	0	0.000	0	0.000	0	0.000	0	0.000	0.0000
	Chronic embolism and thrombosis of popliteal vein. bilateral	I82.533	0	0.000	0	0.000	0	0.000	0	0.000	0.0000
	Chronic embolism and thrombosis of unspecified popliteal vein	I82.539	0	0.000	0	0.000	0	0.000	0	0.000	0.0000
	Chronic embolism and thrombosis of tibial vein	I82.54	0	0.000	0	0.000	0	0.000	0	0.000	0.0000
	Chronic embolism and thrombosis of right tibial vein	I82.541	0	0.000	0	0.000	0	0.000	0	0.000	0.0000
	Chronic embolism and thrombosis of left tibial vein	I82.542	0	0.000	0	0.000	0	0.000	0	0.000	0.0000
	Chronic embolism and thrombosis of tibial vein. bilateral	I82.543	0	0.000	0	0.000	0	0.000	0	0.000	0.0000
	Chronic embolism and thrombosis of unspecified tibial vein	I82.549	0	0.000	0	0.000	0	0.000	0	0.000	0.0000
	Chronic embolism and thrombosis of peroneal vein	I82.55	0	0.000	0	0.000	0	0.000	0	0.000	0.0000
	Chronic embolism and thrombosis of right peroneal vein	I82.551	0	0.000	0	0.000	0	0.000	0	0.000	0.0000
	Chronic embolism and thrombosis of left peroneal vein	I82.552	0	0.000	0	0.000	0	0.000	0	0.000	0.0000
	Chronic embolism and thrombosis of peroneal vein. bilateral	I82.553	0	0.000	0	0.000	0	0.000	0	0.000	0.0000
	Chronic embolism and thrombosis of unspecified peroneal vein	I82.559	0	0.000	0	0.000	0	0.000	0	0.000	0.0000
	Chronic embolism and thrombosis of calf muscular vein	I82.56	0	0.000	0	0.000	0	0.000	0	0.000	0.0000
	Chronic embolism and thrombosis of right calf muscular vein	I82.561	0	0.000	0	0.000	0	0.000	0	0.000	0.0000
	Chronic embolism and thrombosis of left calf muscular vein	I82.562	0	0.000	0	0.000	0	0.000	0	0.000	0.0000
	Chronic embolism and thrombosis of calf muscular vein. bilateral	I82.563	0	0.000	0	0.000	0	0.000	0	0.000	0.0000
	Chronic embolism and thrombosis of unspecified calf muscular vein	I82.569	0	0.000	0	0.000	0	0.000	0	0.000	0.0000
	Chronic embolism and thrombosis of other specified deep vein of lower extremity	I82.59	0	0.000	0	0.000	0	0.000	0	0.000	0.0000
	Chronic embolism and thrombosis of other specified deep vein of right lower extremity	I82.591	0	0.000	0	0.000	0	0.000	0	0.000	0.0000
	Chronic embolism and thrombosis of other specified deep vein of left lower extremity	I82.592	0	0.000	0	0.000	0	0.000	0	0.000	0.0000
	Chronic embolism and thrombosis of other specified deep vein of lower extremity. bilateral	I82.593	0	0.000	0	0.000	0	0.000	0	0.000	0.0000
	Chronic embolism and thrombosis of other specified deep vein of unspecified lower extremity	I82.599	0	0.000	0	0.000	0	0.000	0	0.000	0.0000
	Chronic embolism and thrombosis of unspecified deep veins of proximal lower extremity	I82.5Y	0	0.000	0	0.000	0	0.000	0	0.000	0.0000
	Chronic embolism and thrombosis of unspecified deep veins of right proximal lower extremity	I82.5Y1	0	0.000	0	0.000	0	0.000	0	0.000	0.0000

	Chronic embolism and thrombosis of unspecified deep veins of left proximal lower extremity	I82.5Y2	0	0.000	0	0.000	0	0.000	0	0.000	0.0000
	Chronic embolism and thrombosis of unspecified deep veins of proximal lower extremity. bilateral	I82.5Y3	0	0.000	0	0.000	0	0.000	0	0.000	0.0000
	Chronic embolism and thrombosis of unspecified deep veins of unspecified proximal lower extremity	I82.5Y9	0	0.000	0	0.000	0	0.000	0	0.000	0.0000
	Chronic embolism and thrombosis of unspecified deep veins of distal lower extremity	I82.5Z	0	0.000	0	0.000	0	0.000	0	0.000	0.0000
	Chronic embolism and thrombosis of unspecified deep veins of right distal lower extremity	I82.5Z1	0	0.000	0	0.000	0	0.000	0	0.000	0.0000
	Chronic embolism and thrombosis of unspecified deep veins of left distal lower extremity	I82.5Z2	0	0.000	0	0.000	0	0.000	0	0.000	0.0000
	Chronic embolism and thrombosis of unspecified deep veins of distal lower extremity. bilateral	I82.5Z3	0	0.000	0	0.000	0	0.000	0	0.000	0.0000
	Chronic embolism and thrombosis of unspecified deep veins of unspecified distal lower extremity	I82.5Z9	0	0.000	0	0.000	0	0.000	0	0.000	0.0000
	Acute embolism and thrombosis of veins of upper extremity	I82.6	0	0.000	0	0.000	0	0.000	0	0.000	0.0000
	Acute embolism and thrombosis of unspecified veins of upper extremity	I82.60	0	0.000	0	0.000	0	0.000	0	0.000	0.0000
	Acute embolism and thrombosis of unspecified veins of right upper extremity	I82.601	0	0.000	0	0.000	0	0.000	0	0.000	0.0000
	Acute embolism and thrombosis of unspecified veins of left upper extremity	I82.602	0	0.000	0	0.000	0	0.000	0	0.000	0.0000
	Acute embolism and thrombosis of unspecified veins of upper extremity. bilateral	I82.603	0	0.000	0	0.000	0	0.000	0	0.000	0.0000
	Acute embolism and thrombosis of unspecified veins of unspecified upper extremity	I82.609	0	0.000	0	0.000	0	0.000	0	0.000	0.0000
	Acute embolism and thrombosis of superficial veins of upper extremity	I82.61	0	0.000	0	0.000	0	0.000	0	0.000	0.0000
	Acute embolism and thrombosis of superficial veins of right upper extremity	I82.611	0	0.000	0	0.000	0	0.000	0	0.000	0.0000
	Acute embolism and thrombosis of superficial veins of left upper extremity	I82.612	0	0.000	0	0.000	0	0.000	0	0.000	0.0000
	Acute embolism and thrombosis of superficial veins of upper extremity. bilateral	I82.613	0	0.000	0	0.000	0	0.000	0	0.000	0.0000
	Acute embolism and thrombosis of superficial veins of unspecified upper extremity	I82.619	0	0.000	0	0.000	0	0.000	0	0.000	0.0000
	Acute embolism and thrombosis of deep veins of upper extremity	I82.62	0	0.000	0	0.000	0	0.000	0	0.000	0.0000
	Acute embolism and thrombosis of deep veins of right upper extremity	I82.621	0	0.000	0	0.000	0	0.000	0	0.000	0.0000
	Acute embolism and thrombosis of deep veins of left upper extremity	I82.622	0	0.000	0	0.000	0	0.000	0	0.000	0.0000
	Acute embolism and thrombosis of deep veins of upper extremity. bilateral	I82.623	0	0.000	0	0.000	0	0.000	0	0.000	0.0000
	Acute embolism and thrombosis of deep veins of unspecified upper extremity	I82.629	0	0.000	0	0.000	0	0.000	0	0.000	0.0000
	Chronic embolism and thrombosis of veins of upper extremity	I82.7	0	0.000	0	0.000	0	0.000	0	0.000	0.0000
	Chronic embolism and thrombosis of unspecified veins of upper extremity	I82.70	0	0.000	0	0.000	0	0.000	0	0.000	0.0000
	Chronic embolism and thrombosis of unspecified veins of right upper extremity	I82.701	0	0.000	0	0.000	0	0.000	0	0.000	0.0000
	Chronic embolism and thrombosis of unspecified veins of left upper extremity	I82.702	0	0.000	0	0.000	0	0.000	0	0.000	0.0000
	Chronic embolism and thrombosis of unspecified veins of upper extremity. bilateral	I82.703	0	0.000	0	0.000	0	0.000	0	0.000	0.0000

	Chronic embolism and thrombosis of unspecified veins of unspecified upper extremity	I82.709	0	0.000	0	0.000	0	0.000	0	0.000	0.0000
	Chronic embolism and thrombosis of superficial veins of upper extremity	I82.71	0	0.000	0	0.000	0	0.000	0	0.000	0.0000
	Chronic embolism and thrombosis of superficial veins of right upper extremity	I82.711	0	0.000	0	0.000	0	0.000	0	0.000	0.0000
	Chronic embolism and thrombosis of superficial veins of left upper extremity	I82.712	0	0.000	0	0.000	0	0.000	0	0.000	0.0000
	Chronic embolism and thrombosis of superficial veins of upper extremity. bilateral	I82.713	0	0.000	0	0.000	0	0.000	0	0.000	0.0000
	Chronic embolism and thrombosis of superficial veins of unspecified upper extremity	I82.719	0	0.000	0	0.000	0	0.000	0	0.000	0.0000
	Chronic embolism and thrombosis of deep veins of upper extremity	I82.72	0	0.000	0	0.000	0	0.000	0	0.000	0.0000
	Chronic embolism and thrombosis of deep veins of right upper extremity	I82.721	0	0.000	0	0.000	0	0.000	0	0.000	0.0000
	Chronic embolism and thrombosis of deep veins of left upper extremity	I82.722	0	0.000	0	0.000	0	0.000	0	0.000	0.0000
	Chronic embolism and thrombosis of deep veins of upper extremity. bilateral	I82.723	0	0.000	0	0.000	0	0.000	0	0.000	0.0000
	Chronic embolism and thrombosis of deep veins of unspecified upper extremity	I82.729	0	0.000	0	0.000	0	0.000	0	0.000	0.0000
	Embolism and thrombosis of axillary vein	I82.A	0	0.000	0	0.000	0	0.000	0	0.000	0.0000
	Acute embolism and thrombosis of axillary vein	I82.A1	0	0.000	0	0.000	0	0.000	0	0.000	0.0000
	Acute embolism and thrombosis of right axillary vein	I82.A11	0	0.000	0	0.000	0	0.000	0	0.000	0.0000
	Acute embolism and thrombosis of left axillary vein	I82.A12	0	0.000	0	0.000	0	0.000	0	0.000	0.0000
	Acute embolism and thrombosis of axillary vein. bilateral	I82.A13	0	0.000	0	0.000	0	0.000	0	0.000	0.0000
	Acute embolism and thrombosis of unspecified axillary vein	I82.A19	0	0.000	0	0.000	0	0.000	0	0.000	0.0000
	Chronic embolism and thrombosis of axillary vein	I82.A2	0	0.000	0	0.000	0	0.000	0	0.000	0.0000
	Chronic embolism and thrombosis of right axillary vein	I82.A21	0	0.000	0	0.000	0	0.000	0	0.000	0.0000
	Chronic embolism and thrombosis of left axillary vein	I82.A22	0	0.000	0	0.000	0	0.000	0	0.000	0.0000
	Chronic embolism and thrombosis of axillary vein. bilateral	I82.A23	0	0.000	0	0.000	0	0.000	0	0.000	0.0000
	Chronic embolism and thrombosis of unspecified axillary vein	I82.A29	0	0.000	0	0.000	0	0.000	0	0.000	0.0000
	Embolism and thrombosis of subclavian vein	I82.B	0	0.000	0	0.000	0	0.000	0	0.000	0.0000
	Acute embolism and thrombosis of subclavian vein	I82.B1	0	0.000	0	0.000	0	0.000	0	0.000	0.0000
	Acute embolism and thrombosis of right subclavian vein	I82.B11	0	0.000	0	0.000	0	0.000	0	0.000	0.0000
	Acute embolism and thrombosis of left subclavian vein	I82.B12	0	0.000	0	0.000	0	0.000	0	0.000	0.0000
	Acute embolism and thrombosis of subclavian vein. bilateral	I82.B13	0	0.000	0	0.000	0	0.000	0	0.000	0.0000
	Acute embolism and thrombosis of unspecified subclavian vein	I82.B19	0	0.000	0	0.000	0	0.000	0	0.000	0.0000
	Chronic embolism and thrombosis of subclavian vein	I82.B2	0	0.000	0	0.000	0	0.000	0	0.000	0.0000

	Chronic embolism and thrombosis of right subclavian vein	I82.B21	0	0.000	0	0.000	0	0.000	0	0.000	0.0000
	Chronic embolism and thrombosis of left subclavian vein	I82.B22	0	0.000	0	0.000	0	0.000	0	0.000	0.0000
	Chronic embolism and thrombosis of subclavian vein. bilateral	I82.B23	0	0.000	0	0.000	0	0.000	0	0.000	0.0000
	Chronic embolism and thrombosis of unspecified subclavian vein	I82.B29	0	0.000	0	0.000	0	0.000	0	0.000	0.0000
	Embolism and thrombosis of internal jugular vein	I82.C	0	0.000	0	0.000	0	0.000	0	0.000	0.0000
	Acute embolism and thrombosis of internal jugular vein	I82.C1	0	0.000	0	0.000	0	0.000	0	0.000	0.0000
	Acute embolism and thrombosis of right internal jugular vein	I82.C11	0	0.000	0	0.000	0	0.000	0	0.000	0.0000
	Acute embolism and thrombosis of left internal jugular vein	I82.C12	0	0.000	0	0.000	0	0.000	0	0.000	0.0000
	Acute embolism and thrombosis of internal jugular vein. bilateral	I82.C13	0	0.000	0	0.000	0	0.000	0	0.000	0.0000
	Acute embolism and thrombosis of unspecified internal jugular vein	I82.C19	0	0.000	0	0.000	0	0.000	0	0.000	0.0000
	Chronic embolism and thrombosis of internal jugular vein	I82.C2	0	0.000	0	0.000	0	0.000	0	0.000	0.0000
	Chronic embolism and thrombosis of right internal jugular vein	I82.C21	0	0.000	0	0.000	0	0.000	0	0.000	0.0000
	Chronic embolism and thrombosis of left internal jugular vein	I82.C22	0	0.000	0	0.000	0	0.000	0	0.000	0.0000
	Chronic embolism and thrombosis of internal jugular vein. bilateral	I82.C23	0	0.000	0	0.000	0	0.000	0	0.000	0.0000
	Chronic embolism and thrombosis of unspecified internal jugular vein	I82.C29	0	0.000	0	0.000	0	0.000	0	0.000	0.0000
	Embolism and thrombosis of other specified veins	I82.8	254	1.595	188	1.604	42	1.560	484	1.596	0.9901
	Embolism and thrombosis of superficial veins of lower extremities	I82.81	1	0.006	0	0.000	0	0.000	1	0.003	0.8776
	Embolism and thrombosis of superficial veins of right lower extremity	I82.811	0	0.000	0	0.000	0	0.000	0	0.000	0.0000
	Embolism and thrombosis of superficial veins of left lower extremity	I82.812	0	0.000	0	0.000	0	0.000	0	0.000	0.0000
	Embolism and thrombosis of superficial veins of lower extremities. bilateral	I82.813	0	0.000	0	0.000	0	0.000	0	0.000	0.0000
	Embolism and thrombosis of superficial veins of unspecified lower extremity	I82.819	0	0.000	0	0.000	0	0.000	0	0.000	0.0000
	Embolism and thrombosis of other specified veins	I82.89	0	0.000	0	0.000	0	0.000	0	0.000	0.0000
	Acute embolism and thrombosis of other specified veins	I82.890	0	0.000	0	0.000	0	0.000	0	0.000	0.0000
	Chronic embolism and thrombosis of other specified veins	I82.891	0	0.000	0	0.000	0	0.000	0	0.000	0.0000
	Embolism and thrombosis of unspecified vein	I82.9	89	0.559	53	0.452	12	0.446	154	0.508	0.2542
	Acute embolism and thrombosis of unspecified vein	I82.90	0	0.000	0	0.000	0	0.000	0	0.000	0.0000
	Chronic embolism and thrombosis of unspecified vein	I82.91	0	0.000	0	0.000	0	0.000	0	0.000	0.0000
**Total**			1657	10.406	1188	10.138	310	11.516	3155	10.401	0.4820

Supplementary Table 5: Diseases of the circulatory system

Organ System	Extraintestinal Manifestations and Associated Immune Disorders	ICD-10	Crohn's Disease		Ulcerative Colitis		IBD-Unclassified		IBD-Total (CD + UC + IBDU)		Comparison
			N = 15924		N = 11718		N = 2692		N = 30334		UC vs. CD
			n	%	n	%	n	%	n	%	p value
**Diseases of the circulatory system**											
	**Rheumatic fever without heart involvement**	**I00**	11	0.069	4	0.034	2	0.074	17	0.056	0.3313
	**Rheumatic fever with heart involvement**	**I01**	0	0.000	0	0.000	0	0.000	0	0.000	0.0000
	Acute rheumatic pericarditis	I01.0	0	0.000	0	0.000	0	0.000	0	0.000	0.0000
	Acute rheumatic endocarditis	I01.1	0	0.000	0	0.000	0	0.000	0	0.000	0.0000
	Acute rheumatic myocarditis	I01.2	0	0.000	0	0.000	0	0.000	0	0.000	0.0000
	Other acute rheumatic heart disease	I01.8	0	0.000	0	0.000	0	0.000	0	0.000	0.0000
	Acute rheumatic heart disease. unspecified	I01.9	1	0.006	0	0.000	1	0.037	2	0.007	0.8776
	**Rheumatic chorea**	**I02**	0	0.000	0	0.000	0	0.000	0	0.000	0.0000
	Rheumatic chorea with heart involvement	I02.0	0	0.000	0	0.000	0	0.000	0	0.000	0.0000
	Rheumatic chorea without heart involvement	I02.9	0	0.000	1	0.009	0	0.000	1	0.003	0.8776
	**Rheumatic mitral valve diseases**	**I05**	0	0.000	0	0.000	0	0.000	0	0.000	0.0000
	Rheumatic mitral stenosis	I05.0	10	0.063	6	0.051	1	0.037	17	0.056	0.8862
	Rheumatic mitral insufficiency	I05.1	6	0.038	8	0.068	2	0.074	16	0.053	0.3972
	Rheumatic mitral stenosis with insufficiency	I05.2	6	0.038	4	0.034	1	0.037	11	0.036	0.8674
	Other rheumatic mitral valve diseases	I05.8	1	0.006	3	0.026	0	0.000	4	0.013	0.4157
	Rheumatic mitral valve disease. unspecified	I05.9	24	0.151	27	0.230	9	0.334	60	0.198	0.1663
	**Rheumatic aortic valve diseases**	**I06**	0	0.000	0	0.000	0	0.000	0	0.000	0.0000
	Rheumatic aortic stenosis	I06.0	1	0.006	1	0.009	0	0.000	2	0.007	0.6187
	Rheumatic aortic insufficiency	I06.1	0	0.000	0	0.000	0	0.000	0	0.000	0.0000
	Rheumatic aortic stenosis with insufficiency	I06.2	0	0.000	0	0.000	0	0.000	0	0.000	0.0000

	Other rheumatic aortic valve diseases	I06.8	0	0.000	0	0.000	0	0.000	0	0.000	0.0000
	Rheumatic aortic valve disease. unspecified	I06.9	10	0.063	8	0.068	1	0.037	19	0.063	0.9503
	**Rheumatic tricuspid valve diseases**	**I07**	0	0.000	0	0.000	0	0.000	0	0.000	0.0000
	Rheumatic tricuspid stenosis	I07.0	0	0.000	0	0.000	0	0.000	0	0.000	0.0000
	Rheumatic tricuspid insufficiency	I07.1	25	0.157	12	0.102	3	0.111	40	0.132	0.2890
	Rheumatic tricuspid stenosis and insufficiency	I07.2	0	0.000	0	0.000	0	0.000	0	0.000	0.0000
	Other rheumatic tricuspid valve diseases	I07.8	3	0.019	0	0.000	0	0.000	3	0.010	0.3672
	Rheumatic tricuspid valve disease. unspecified	I07.9	4	0.025	1	0.009	0	0.000	5	0.016	0.5750
	**Multiple valve diseases**	**I08**	0	0.000	0	0.000	0	0.000	0	0.000	0.0000
	Rheumatic disorders of both mitral and aortic valves	I08.0	21	0.132	20	0.171	3	0.111	44	0.145	0.5027
	Rheumatic disorders of both mitral and tricuspid valves	I08.1	15	0.094	16	0.137	5	0.186	36	0.119	0.3911
	Rheumatic disorders of both aortic and tricuspid valves	I08.2	2	0.013	1	0.009	1	0.037	4	0.013	0.7897
	Combined rheumatic disorders of mitral. aortic and tricuspid valves	I08.3	11	0.069	8	0.068	3	0.111	22	0.073	0.8361
	Other rheumatic multiple valve diseases	I08.8	0	0.000	0	0.000	0	0.000	0	0.000	0.0000
	Rheumatic multiple valve disease. unspecified	I08.9	14	0.088	10	0.085	6	0.223	30	0.099	0.8929
	**Other rheumatic heart diseases**	**I09**	0	0.000	0	0.000	0	0.000	0	0.000	0.0000
	Rheumatic myocarditis	I09.0	0	0.000	0	0.000	0	0.000	0	0.000	0.0000
	Rheumatic diseases of endocardium. valve unspecified	I09.1	1	0.006	0	0.000	1	0.037	2	0.007	0.8776
	Chronic rheumatic pericarditis	I09.2	0	0.000	0	0.000	0	0.000	0	0.000	0.0000
	Other specified rheumatic heart diseases	I09.8	4	0.025	2	0.017	0	0.000	6	0.020	0.9713
	Rheumatic heart failure	I09.81	0	0.000	0	0.000	0	0.000	0	0.000	0.0000
	Other specified rheumatic heart diseases	I09.89	0	0.000	0	0.000	0	0.000	0	0.000	0.0000
	Rheumatic heart disease. unspecified	I09.9	6	0.038	4	0.034	2	0.074	12	0.040	0.8674
	**Angina pectoris**	**I20**	0	0.000	0	0.000	0	0.000	0	0.000	0.0000
	Unstable angina	I20.0	243	1.526	209	1.784	61	2.266	513	1.691	0.1051
	Angina pectoris with documented spasm	I20.1	9	0.057	6	0.051	4	0.149	19	0.063	0.9412
	Refractory angina pectoris	I20.2	0	0.000	0	0.000	0	0.000	0	0.000	0.0000
	Other forms of angina pectoris	I20.8	0	0.000	0	0.000	0	0.000	0	0.000	0.0000
	Angina pectoris. unspecified	I20.9	388	2.437	337	2.876	97	3.603	822	2.710	0.0264

	**Acute myocardial infarction**	**I21**	0	0.000	0	0.000	0	0.000	0	0.000	0.0000
	ST elevation	I21.0	53	0.333	45	0.384	12	0.446	110	0.363	0.5450
	ST elevation	I21.01	0	0.000	0	0.000	0	0.000	0	0.000	0.0000
	ST elevation	I21.02	0	0.000	0	0.000	0	0.000	0	0.000	0.0000
	ST elevation	I21.09	0	0.000	0	0.000	0	0.000	0	0.000	0.0000
	ST elevation	I21.1	67	0.421	52	0.444	19	0.706	138	0.455	0.8447
	ST elevation	I21.11	0	0.000	0	0.000	0	0.000	0	0.000	0.0000
	ST elevation	I21.19	0	0.000	0	0.000	0	0.000	0	0.000	0.0000
	ST elevation	I21.2	13	0.082	12	0.102	1	0.037	26	0.086	0.7150
	ST elevation	I21.21	0	0.000	0	0.000	0	0.000	0	0.000	0.0000
	ST elevation	I21.29	0	0.000	0	0.000	0	0.000	0	0.000	0.0000
	ST elevation	I21.3	39	0.245	29	0.247	3	0.111	71	0.234	0.9360
	Non-ST elevation	I21.4	245	1.539	182	1.553	66	2.452	493	1.625	0.9617
	Acute myocardial infarction. unspecified	I21.9	184	1.155	141	1.203	45	1.672	370	1.220	0.7582
	Other type of myocardial infarction	I21.A	0	0.000	0	0.000	0	0.000	0	0.000	0.0000
	Myocardial infarction type 2	I21.A1	0	0.000	0	0.000	0	0.000	0	0.000	0.0000
	Other myocardial infarction type	I21.A9	0	0.000	0	0.000	0	0.000	0	0.000	0.0000
	**Subsequent ST elevation**	**I22**	0	0.000	0	0.000	0	0.000	0	0.000	0.0000
	Subsequent ST elevation	I22.0	1	0.006	0	0.000	0	0.000	1	0.003	0.8776
	Subsequent ST elevation	I22.1	2	0.013	1	0.009	0	0.000	3	0.010	0.7897
	Subsequent non-ST elevation	I22.2	0	0.000	0	0.000	0	0.000	0	0.000	0.0000
	Subsequent ST elevation	I22.8	0	0.000	0	0.000	0	0.000	0	0.000	0.0000
	Subsequent ST elevation	I22.9	1	0.006	3	0.026	0	0.000	4	0.013	0.4157
	**Certain current complications following ST elevation**	**I23**	0	0.000	0	0.000	0	0.000	0	0.000	0.0000
	Hemopericardium as current complication following acute myocardial infarction	I23.0	0	0.000	0	0.000	0	0.000	0	0.000	0.0000
	Atrial septal defect as current complication following acute myocardial infarction	I23.1	0	0.000	0	0.000	0	0.000	0	0.000	0.0000
	Ventricular septal defect as current complication following acute myocardial infarction	I23.2	0	0.000	1	0.009	0	0.000	1	0.003	0.8776
	Rupture of cardiac wall without hemopericardium as current complication following acute myocardial infarction	I23.3	0	0.000	0	0.000	0	0.000	0	0.000	0.0000

	Rupture of chordae tendineae as current complication following acute myocardial infarction	I23.4	0	0.000	0	0.000	0	0.000	0	0.000	0.0000
	Rupture of papillary muscle as current complication following acute myocardial infarction	I23.5	1	0.006	1	0.009	0	0.000	2	0.007	0.6187
	Thrombosis of atrium. auricular appendage. and ventricle as current complications following acute myocardial infarction	I23.6	2	0.013	0	0.000	0	0.000	2	0.007	0.6187
	Postinfarction angina	I23.7	0	0.000	0	0.000	0	0.000	0	0.000	0.0000
	Other current complications following acute myocardial infarction	I23.8	0	0.000	0	0.000	0	0.000	0	0.000	0.0000
	**Other acute ischemic heart diseases**	**I24**	0	0.000	0	0.000	0	0.000	0	0.000	0.0000
	Acute coronary thrombosis not resulting in myocardial infarction	I24.0	8	0.050	7	0.060	1	0.037	16	0.053	0.9412
	Dressler's syndrome	I24.1	2	0.013	0	0.000	0	0.000	2	0.007	0.6187
	Other forms of acute ischemic heart disease	I24.8	12	0.075	15	0.128	2	0.074	29	0.096	0.2341
	Acute ischemic heart disease. unspecified	I24.9	192	1.206	147	1.254	49	1.820	388	1.279	0.7576
	**Pulmonary embolism**	**I26**	0	0.000	0	0.000	0	0.000	0	0.000	0.0000
	Pulmonary embolism with acute cor pulmonale	I26.0	10	0.063	10	0.085	3	0.111	23	0.076	0.6438
	Septic pulmonary embolism with acute cor pulmonale	I26.01	0	0.000	0	0.000	0	0.000	0	0.000	0.0000
	Saddle embolus of pulmonary artery with acute cor pulmonale	I26.02	0	0.000	0	0.000	0	0.000	0	0.000	0.0000
	Other pulmonary embolism with acute cor pulmonale	I26.09	0	0.000	0	0.000	0	0.000	0	0.000	0.0000
	Pulmonary embolism without acute cor pulmonale	I26.9	639	4.013	480	4.096	115	4.272	1234	4.068	0.7512
	Septic pulmonary embolism without acute cor pulmonale	I26.90	0	0.000	0	0.000	0	0.000	0	0.000	0.0000
	Saddle embolus of pulmonary artery without acute cor pulmonale	I26.92	0	0.000	0	0.000	0	0.000	0	0.000	0.0000
	Single subsegmental pulmonary embolism without acute cor pulmonale	I26.93	0	0.000	0	0.000	0	0.000	0	0.000	0.0000
	Multiple subsegmental pulmonary emboli without acute cor pulmonale	I26.94	0	0.000	0	0.000	0	0.000	0	0.000	0.0000
	Other pulmonary embolism without acute cor pulmonale	I26.99	0	0.000	0	0.000	0	0.000	0	0.000	0.0000
	**Acute pericarditis**	**I30**	0	0.000	0	0.000	0	0.000	0	0.000	0.0000
	Acute nonspecific idiopathic pericarditis	I30.0	2	0.013	1	0.009	0	0.000	3	0.010	0.7897
	Infective pericarditis	I30.1	6	0.038	6	0.051	1	0.037	13	0.043	0.8093
	Other forms of acute pericarditis	I30.8	3	0.019	3	0.026	2	0.074	8	0.026	0.9713
	Acute pericarditis. unspecified	I30.9	34	0.214	25	0.213	5	0.186	64	0.211	0.8974
	Pericarditis in diseases classified elsewhere	I32	2	0.013	0	0.000	0	0.000	2	0.007	0.6187
	**Acute myocarditis**	**I40**	0	0.000	0	0.000	0	0.000	0	0.000	0.0000
	Isolated myocarditis	I40.1	0	0.000	0	0.000	0	0.000	0	0.000	0.0000

	Other acute myocarditis	I40.8	0	0.000	0	0.000	0	0.000	0	0.000	0.0000
	Acute myocarditis. unspecified	I40.9	5	0.031	2	0.017	2	0.074	9	0.030	0.7207
	Myocarditis in diseases classified elsewhere	I41	0	0.000	0	0.000	0	0.000	0	0.000	0.0000
**Total**			1584	9.947	1240	10.582	322	11.961	3146	10.371	0.0888

Supplementary Table 6: Diseases of the respiratory system

Organ System	Extraintestinal Manifestations and Associated Immune Disorders	ICD-10	Crohn's Disease		Ulcerative Colitis		IBD-Unclassified		IBD-Total (CD + UC + IBDU)		Comparison
			N = 15924		N = 11718		N = 2692		N = 30334		UC vs. CD
			n		n		n		n		p value
				%		%		%		%	≤
**Diseases of the respiratory system**											
	**Acute laryngitis and tracheitis**	**J04**	0	0.000	0	0.000	0	0.000	0	0.000	0.0000
	Acute laryngitis	J04.0	141	0.885	107	0.913	26	0.966	274	0.903	0.8599
	Acute tracheitis	J04.1	33	0.207	19	0.162	7	0.260	59	0.195	0.4749
	Acute tracheitis without obstruction	J04.10	0	0.000	0	0.000	0	0.000	0	0.000	0.0000
	Acute tracheitis with obstruction	J04.11	0	0.000	0	0.000	0	0.000	0	0.000	0.0000
	Acute laryngotracheitis	J04.2	12	0.075	3	0.026	0	0.000	15	0.049	0.1352
	Supraglottitis. unspecified	J04.3	0	0.000	0	0.000	0	0.000	0	0.000	0.0000
	Supraglottitis. unspecified. without obstruction	J04.30	0	0.000	0	0.000	0	0.000	0	0.000	0.0000
	Supraglottitis. unspecified. with obstruction	J04.31	0	0.000	0	0.000	0	0.000	0	0.000	0.0000
	**Acute obstructive laryngitis [croup] and epiglottitis**	**J05**	0	0.000	0	0.000	0	0.000	0	0.000	0.0000
	Acute obstructive laryngitis [croup]	J05.0	78	0.490	50	0.427	8	0.297	136	0.448	0.5001
	Acute epiglottitis	J05.1	17	0.107	12	0.102	4	0.149	33	0.109	0.9382
	Acute epiglottitis without obstruction	J05.10	0	0.000	0	0.000	0	0.000	0	0.000	0.0000
	Acute epiglottitis with obstruction	J05.11	0	0.000	0	0.000	0	0.000	0	0.000	0.0000
	**Acute bronchiolitis**	**J21**	0	0.000	0	0.000	0	0.000	0	0.000	0.0000
	Acute bronchiolitis due to respiratory syncytial virus	J21.0	4	0.025	4	0.034	2	0.074	10	0.033	0.9380
	Acute bronchiolitis due to human metapneumovirus	J21.1	0	0.000	0	0.000	0	0.000	0	0.000	0.0000
	Acute bronchiolitis due to other specified organisms	J21.8	1	0.006	4	0.034	0	0.000	5	0.016	0.2115
	Acute bronchiolitis. unspecified	J21.9	52	0.327	34	0.290	9	0.334	95	0.313	0.6689
	**Asthma**	**J45**	0	0.000	0	0.000	0	0.000	0	0.000	0.0000

	Mild intermittent asthma	J45.2	0	0.000	0	0.000	0	0.000	0	0.000	0.0000
	Mild intermittent asthma. uncomplicated	J45.20	0	0.000	0	0.000	0	0.000	0	0.000	0.0000
	Mild intermittent asthma with	J45.21	0	0.000	0	0.000	0	0.000	0	0.000	0.0000
	Mild intermittent asthma with status asthmaticus	J45.22	0	0.000	0	0.000	0	0.000	0	0.000	0.0000
	Mild persistent asthma	J45.3	0	0.000	0	0.000	0	0.000	0	0.000	0.0000
	Mild persistent asthma. uncomplicated	J45.30	0	0.000	0	0.000	0	0.000	0	0.000	0.0000
	Mild persistent asthma with	J45.31	0	0.000	0	0.000	0	0.000	0	0.000	0.0000
	Mild persistent asthma with status asthmaticus	J45.32	0	0.000	0	0.000	0	0.000	0	0.000	0.0000
	Moderate persistent asthma	J45.4	0	0.000	0	0.000	0	0.000	0	0.000	0.0000
	Moderate persistent asthma. uncomplicated	J45.40	0	0.000	0	0.000	0	0.000	0	0.000	0.0000
	Moderate persistent asthma with	J45.41	0	0.000	0	0.000	0	0.000	0	0.000	0.0000
	Moderate persistent asthma with status asthmaticus	J45.42	0	0.000	0	0.000	0	0.000	0	0.000	0.0000
	Severe persistent asthma	J45.5	0	0.000	0	0.000	0	0.000	0	0.000	0.0000
	Severe persistent asthma. uncomplicated	J45.50	0	0.000	0	0.000	0	0.000	0	0.000	0.0000
	Severe persistent asthma with	J45.51	0	0.000	0	0.000	0	0.000	0	0.000	0.0000
	Severe persistent asthma with status asthmaticus	J45.52	0	0.000	0	0.000	0	0.000	0	0.000	0.0000
	Other and unspecified asthma	J45.9	0	0.000	0	0.000	0	0.000	0	0.000	0.0000
	Unspecified asthma	J45.90	1299	8.157	838	7.151	21<br>8	8.098	235<br>5	7.764	0.0021
	Unspecified asthma with	J45.901	42	0.264	30	0.256	6	0.223	78	0.257	0.9958
	Unspecified asthma with status asthmaticus	J45.902	0	0.000	0	0.000	0	0.000	0	0.000	0.0000
	Unspecified asthma. uncomplicated	J45.909	0	0.000	0	0.000	0	0.000	0	0.000	0.0000
	Other asthma	J45.99	0	0.000	0	0.000	0	0.000	0	0.000	0.0000
	Exercise induced bronchospasm	J45.990	0	0.000	0	0.000	0	0.000	0	0.000	0.0000
	Cough variant asthma	J45.991	0	0.000	0	0.000	0	0.000	0	0.000	0.0000
	Other asthma	J45.998	0	0.000	0	0.000	0	0.000	0	0.000	0.0000
	**Other interstitial pulmonary diseases**	**J84**	0	0.000	0	0.000	0	0.000	0	0.000	0.0000
	Alveolar and parieto-alveolar conditions	J84.0	1	0.006	5	0.043	0	0.000	6	0.020	0.1060
	Alveolar proteinosis	J84.01	0	0.000	0	0.000	0	0.000	0	0.000	0.0000
	Pulmonary alveolar microlithiasis	J84.02	0	0.000	0	0.000	0	0.000	0	0.000	0.0000

	Idiopathic pulmonary hemosiderosis	J84.03	0	0.000	0	0.000	0	0.000	0	0.000	0.0000
	Other alveolar and parieto-alveolar conditions	J84.09	0	0.000	0	0.000	0	0.000	0	0.000	0.0000
	Other interstitial pulmonary diseases with fibrosis	J84.1	64	0.402	60	0.512	14	0.520	138	0.455	0.2066
	Pulmonary fibrosis. unspecified	J84.10	0	0.000	0	0.000	0	0.000	0	0.000	0.0000
	Idiopathic interstitial pneumonia	J84.11	0	0.000	0	0.000	0	0.000	0	0.000	0.0000
	Idiopathic interstitial pneumonia. not otherwise specified	J84.111	0	0.000	0	0.000	0	0.000	0	0.000	0.0000
	Idiopathic pulmonary fibrosis	J84.112	0	0.000	0	0.000	0	0.000	0	0.000	0.0000
	Idiopathic non-specific interstitial pneumonitis	J84.113	0	0.000	0	0.000	0	0.000	0	0.000	0.0000
	Acute interstitial pneumonitis	J84.114	0	0.000	0	0.000	0	0.000	0	0.000	0.0000
	Respiratory bronchiolitis interstitial lung disease	J84.115	0	0.000	0	0.000	0	0.000	0	0.000	0.0000
	Cryptogenic organizing pneumonia	J84.116	0	0.000	0	0.000	0	0.000	0	0.000	0.0000
	Desquamative interstitial pneumonia	J84.117	0	0.000	0	0.000	0	0.000	0	0.000	0.0000
	Other interstitial pulmonary diseases with fibrosis in diseases classified elsewhere	J84.17	0	0.000	0	0.000	0	0.000	0	0.000	0.0000
	Interstitial lung disease with progressive fibrotic phenotype in diseases classified elsewhere	J84.170	0	0.000	0	0.000	0	0.000	0	0.000	0.0000
	Other interstitial pulmonary diseases with fibrosis in diseases classified elsewhere	J84.178	0	0.000	0	0.000	0	0.000	0	0.000	0.0000
	Lymphoid interstitial pneumonia	J84.2	0	0.000	0	0.000	0	0.000	0	0.000	0.0000
	Other specified interstitial pulmonary diseases	J84.8	15	0.094	13	0.111	4	0.149	32	0.105	0.8095
	Lymphangioleiomyomatosis	J84.81	0	0.000	0	0.000	0	0.000	0	0.000	0.0000
	Adult pulmonary Langerhans cell histiocytosis	J84.82	0	0.000	0	0.000	0	0.000	0	0.000	0.0000
	Surfactant mutations of the lung	J84.83	0	0.000	0	0.000	0	0.000	0	0.000	0.0000
	Other interstitial lung diseases of childhood	J84.84	0	0.000	0	0.000	0	0.000	0	0.000	0.0000
	Neuroendocrine cell hyperplasia of infancy	J84.841	0	0.000	0	0.000	0	0.000	0	0.000	0.0000
	Pulmonary interstitial glycogenosis	J84.842	0	0.000	0	0.000	0	0.000	0	0.000	0.0000
	Alveolar capillary dysplasia with vein misalignment	J84.843	0	0.000	0	0.000	0	0.000	0	0.000	0.0000
	Other interstitial lung diseases of childhood	J84.848	0	0.000	0	0.000	0	0.000	0	0.000	0.0000
	Other specified interstitial pulmonary diseases	J84.89	0	0.000	0	0.000	0	0.000	0	0.000	0.0000
	Interstitial pulmonary disease. unspecified	J84.9	72	0.452	59	0.503	12	0.446	143	0.471	0.5991

**Total**			1658	10.412	1092	9.319	27<br>6	10.25<br>3	302<br>6	9.976	0.0029

Supplementary Table 7: Symptoms. signs. and abnormal clinical and laboratory findings. not elsewhere classified

**Organ System**	**Extraintestinal Manifestations and Associated Immune Disorders**	**ICD-10**	**Crohn's Disease**		**Ulcerative Colitis**		**IBD-Unclassified**		**IBD-Total (CD + UC + IBDU)**		**Comparison**
			**N = 15924**		**N = 11718**		**N = 2692**		**N = 30334**		**UC vs. CD**
			**n**		**n**		**n**		**n**		**p value**
				**%**		**%**		**%**		**%**	
**Symptoms. signs and abnormal clinical and laboratory findings. not elsewhere classified**											
	**Malaise and fatigue**	**R53**	1415	8.886	916	7.817	275	10.215	2606	8.591	0.0017
	Weakness	R53.1	0	0.000	0	0.000	0	0.000	0	0.000	0.0000
	Other malaise and fatigue	R53.8	0	0.000	0	0.000	0	0.000	0	0.000	0.0000
	Other malaise	R53.81	0	0.000	0	0.000	0	0.000	0	0.000	0.0000
	Chronic fatigue. unspecified	R53.82	0	0.000	0	0.000	0	0.000	0	0.000	0.0000
	Other fatigue	R53.83	0	0.000	0	0.000	0	0.000	0	0.000	0.0000
**Total**			1415	8.886	916	7.817	275	10.215	2606	8.591	0.0017

Supplementary Table 8: Diseases of the digestive system

Organ System	Extraintestinal Manifestations and Associated Immune Disorders	ICD-10	Crohn's Disease		Ulcerative Colitis		IBD-Unclassified		IBD-Total (CD + UC + IBDU)		Comparison
			N = 15924		N = 11718		N = 2692		N = 30334		UC vs. CD
			n		n		n		n		p value
				%		%		%		%	
**Diseases of the digestive system**											
	**Vascular disorders of intestine**	**K55**	0	0.000	0	0.000	0	0.000	0	0.000	0.0000
	Acute vascular disorders of intestine	K55.0	160	1.005	112	0.956	51	1.895	323	1.065	0.7293
	Acute	K55.01	0	0.000	0	0.000	0	0.000	0	0.000	0.0000
	Focal	K55.011	0	0.000	0	0.000	0	0.000	0	0.000	0.0000
	Diffuse acute	K55.012	0	0.000	0	0.000	0	0.000	0	0.000	0.0000
	Acute	K55.019	0	0.000	0	0.000	0	0.000	0	0.000	0.0000
	Acute infarction of small intestine	K55.02	0	0.000	0	0.000	0	0.000	0	0.000	0.0000
	Focal	K55.021	0	0.000	0	0.000	0	0.000	0	0.000	0.0000
	Diffuse acute infarction of small intestine	K55.022	0	0.000	0	0.000	0	0.000	0	0.000	0.0000
	Acute infarction of small intestine. extent unspecified	K55.029	0	0.000	0	0.000	0	0.000	0	0.000	0.0000
	Acute	K55.03	0	0.000	0	0.000	0	0.000	0	0.000	0.0000
	Focal	K55.031	0	0.000	0	0.000	0	0.000	0	0.000	0.0000
	Diffuse acute	K55.032	0	0.000	0	0.000	0	0.000	0	0.000	0.0000
	Acute	K55.039	0	0.000	0	0.000	0	0.000	0	0.000	0.0000
	Acute infarction of large intestine	K55.04	0	0.000	0	0.000	0	0.000	0	0.000	0.0000
	Focal	K55.041	0	0.000	0	0.000	0	0.000	0	0.000	0.0000
	Diffuse acute infarction of large intestine	K55.042	0	0.000	0	0.000	0	0.000	0	0.000	0.0000
	Acute infarction of large intestine. extent unspecified	K55.049	0	0.000	0	0.000	0	0.000	0	0.000	0.0000
	Acute	K55.05	0	0.000	0	0.000	0	0.000	0	0.000	0.0000

	Focal	K55.051	0	0.000	0	0.000	0	0.000	0	0.000	0.0000
	Diffuse acute	K55.052	0	0.000	0	0.000	0	0.000	0	0.000	0.0000
	Acute	K55.059	0	0.000	0	0.000	0	0.000	0	0.000	0.0000
	Acute infarction of intestine. part unspecified	K55.06	0	0.000	0	0.000	0	0.000	0	0.000	0.0000
	Focal	K55.061	0	0.000	0	0.000	0	0.000	0	0.000	0.0000
	Diffuse acute infarction of intestine. part unspecified	K55.062	0	0.000	0	0.000	0	0.000	0	0.000	0.0000
	Acute infarction of intestine. part and extent unspecified	K55.069	0	0.000	0	0.000	0	0.000	0	0.000	0.0000
	Chronic vascular disorders of intestine	K55.1	41	0.257	30	0.256	12	0.446	83	0.274	0.9231
	Angiodysplasia of colon	K55.2	9	0.057	9	0.077	4	0.149	22	0.073	0.6783
	Angiodysplasia of colon without hemorrhage	K55.20	12	0.075	12	0.102	7	0.260	31	0.102	0.5837
	Angiodysplasia of colon with hemorrhage	K55.21	28	0.176	49	0.418	13	0.483	90	0.297	0.0003
	Necrotizing enterocolitis	K55.3	0	0.000	0	0.000	0	0.000	0	0.000	0.0000
	Necrotizing enterocolitis. unspecified	K55.30	0	0.000	0	0.000	0	0.000	0	0.000	0.0000
	Stage 1 necrotizing enterocolitis	K55.31	0	0.000	0	0.000	0	0.000	0	0.000	0.0000
	Stage 2 necrotizing enterocolitis	K55.32	0	0.000	0	0.000	0	0.000	0	0.000	0.0000
	Stage 3 necrotizing enterocolitis	K55.33	0	0.000	0	0.000	0	0.000	0	0.000	0.0000
	Other vascular disorders of intestine	K55.8	21	0.132	15	0.128	5	0.186	41	0.135	0.9357
	Vascular disorder of intestine. unspecified	K55.9	208	1.306	265	2.261	91	3.380	564	1.859	0.0000
	Granuloma and granuloma-like lesions of oral mucosa	K13.4	5	0.031	1	0.009	0	0.000	6	0.020	0.3886
	Eosinophilic esophagitis	K20.0	0	0.000	0	0.000	0	0.000	0	0.000	0.0000
	Phlebitis of portal vein	K75.1	2	0.013	0	0.000	0	0.000	2	0.007	0.6187
	Nonspecific reactive hepatitis	K75.2	1	0.006	0	0.000	1	0.037	2	0.007	0.8776
	Granulomatous hepatitis. not elsewhere classified	K75.3	5	0.031	2	0.017	2	0.074	9	0.030	0.7207
	Autoimmune hepatitis	K75.4	53	0.333	66	0.563	11	0.409	130	0.429	0.0051
	Other specified inflammatory liver diseases	K75.8	83	0.521	37	0.316	9	0.334	129	0.425	0.0133
	Other specified inflammatory liver diseases	K75.89	0	0.000	0	0.000	0	0.000	0	0.000	0.0000
	Inflammatory liver disease. unspecified	K75.9	133	0.835	97	0.828	22	0.817	252	0.831	0.9998
	Cholangitis	K83.0	119	0.747	145	1.237	21	0.780	285	0.940	0.0000
	Primary sclerosing cholangitis	K83.01	4	0.025	7	0.060	2	0.074	13	0.043	0.2623

	Other cholangitis	K83.09	0	0.000	0	0.000	0	0.000	0	0.000	0.0000
	**Other chronic pancreatitis (Autoimmune pancreatitis)**	**K86.1**	142	0.892	70	0.597	28	1.040	240	0.791	0.0069
	Celiac disease	K90.0	374	2.349	250	2.133	80	2.972	704	2.321	0.2504
**Total**			1209	7.592	968	8.261	290	10.773	2467	8.133	0.0438

Supplementary Table 9: Diseases of the blood and blood-forming organs and certain disorders involving the immune mechanism

Organ System	Extraintestinal Manifestations and Associated Immune Disorders	ICD-10	Crohn's Disease		Ulcerative Colitis		IBD-Unclassified		IBD-Total (CD + UC + IBDU)		Comparison
			N = 15924		N = 11718		N = 2692		N = 30334		UC vs. CD
			n	%	n	%	n	%	n	%	p value
**Diseases of the blood and blood-forming organs and certain disorders involving the immune mechanism**											
	**Acquired hemolytic anemia**	**D59**	0	0.000	0	0.000	0	0.000	0	0.000	0.0000
	Drug-induced autoimmune hemolytic anemia	D59.0	1	0.006	1	0.009	0	0.000	2	0.007	0.6187
	Other autoimmune hemolytic anemias	D59.1	14	0.088	12	0.102	1	0.037	27	0.089	0.8495
	Autoimmune hemolytic anemia. unspecified	D59.10	0	0.000	0	0.000	0	0.000	0	0.000	0.0000
	Warm autoimmune hemolytic anemia	D59.11	0	0.000	0	0.000	0	0.000	0	0.000	0.0000
	Cold autoimmune hemolytic anemia	D59.12	0	0.000	0	0.000	0	0.000	0	0.000	0.0000
	Mixed type autoimmune hemolytic anemia	D59.13	0	0.000	0	0.000	0	0.000	0	0.000	0.0000
	Other autoimmune hemolytic anemia	D59.19	0	0.000	0	0.000	0	0.000	0	0.000	0.0000
	**Anemia in chronic diseases classified elsewhere**	**D63**	0	0.000	0	0.000	0	0.000	0	0.000	0.0000
	Anemia in other chronic diseases classified elsewhere	D63.8	190	1.193	106	0.905	31	1.152	327	1.078	0.0248
	**Disseminated intravascular coagulation [defibrination syndrome]**	**D65**	17	0.107	10	0.085	5	0.186	32	0.105	0.7125
	**Hereditary factor VIII deficiency**	**D66**	15	0.094	5	0.043	3	0.111	23	0.076	0.1776
	**Hereditary factor IX deficiency**	**D67**	1	0.006	2	0.017	0	0.000	3	0.010	0.7897
	**Other coagulation defects**	**D68**	0	0.000	0	0.000	0	0.000	0	0.000	0.0000
	Von Willebrand disease	D68.0	11	0.069	3	0.026	1	0.037	15	0.049	0.1878
	Von Willebrand disease. unspecified	D68.00	0	0.000	0	0.000	0	0.000	0	0.000	0.0000
	Von Willebrand disease. type 1	D68.01	0	0.000	0	0.000	0	0.000	0	0.000	0.0000
	Von Willebrand disease. type 2	D68.02	0	0.000	0	0.000	0	0.000	0	0.000	0.0000
	Von Willebrand disease. type 2A	D68.020	0	0.000	0	0.000	0	0.000	0	0.000	0.0000

	Von Willebrand disease. type 2B	D68.021	0	0.000	0	0.000	0	0.000	0	0.000	0.0000
	Von Willebrand disease. type 2M	D68.022	0	0.000	0	0.000	0	0.000	0	0.000	0.0000
	Von Willebrand disease. type 2N	D68.023	0	0.000	0	0.000	0	0.000	0	0.000	0.0000
	Von Willebrand disease. type 2. unspecified	D68.029	0	0.000	0	0.000	0	0.000	0	0.000	0.0000
	Von Willebrand disease. type 3	D68.03	0	0.000	0	0.000	0	0.000	0	0.000	0.0000
	Acquired von Willebrand disease	D68.04	0	0.000	0	0.000	0	0.000	0	0.000	0.0000
	Other von Willebrand disease	D68.09	0	0.000	0	0.000	0	0.000	0	0.000	0.0000
	Hereditary factor XI deficiency	D68.1	1	0.006	0	0.000	1	0.037	2	0.007	0.8776
	Hereditary deficiency of other clotting factors	D68.2	13	0.082	6	0.051	1	0.037	20	0.066	0.4704
	Hemorrhagic disorder due to circulating anticoagulants	D68.3	69	0.433	64	0.546	19	0.706	152	0.501	0.2105
	Hemorrhagic disorder due to intrinsic circulating anticoagulants. antibodies. or inhibitors	D68.31	0	0.000	0	0.000	0	0.000	0	0.000	0.0000
	Acquired hemophilia	D68.311	0	0.000	0	0.000	0	0.000	0	0.000	0.0000
	Antiphospholipid antibody with hemorrhagic disorder	D68.312	0	0.000	0	0.000	0	0.000	0	0.000	0.0000
	Other hemorrhagic disorder due to intrinsic circulating anticoagulants. antibodies. or inhibitors	D68.318	0	0.000	0	0.000	0	0.000	0	0.000	0.0000
	Hemorrhagic disorder due to extrinsic circulating anticoagulants	D68.32	0	0.000	0	0.000	0	0.000	0	0.000	0.0000
	Acquired coagulation factor deficiency	D68.4	11	0.069	3	0.026	1	0.037	15	0.049	0.1878
	Primary thrombophilia	D68.5	34	0.214	32	0.273	3	0.111	69	0.227	0.3799
	Activated protein C resistance	D68.51	0	0.000	0	0.000	0	0.000	0	0.000	0.0000
	Prothrombin gene mutation	D68.52	0	0.000	0	0.000	0	0.000	0	0.000	0.0000
	Other primary thrombophilia	D68.59	0	0.000	0	0.000	0	0.000	0	0.000	0.0000
	Other thrombophilia	D68.6	27	0.170	15	0.128	7	0.260	49	0.162	0.4714
	Antiphospholipid syndrome	D68.61	0	0.000	0	0.000	0	0.000	0	0.000	0.0000
	Lupus anticoagulant syndrome	D68.62	0	0.000	0	0.000	0	0.000	0	0.000	0.0000
	Other thrombophilia	D68.69	0	0.000	0	0.000	0	0.000	0	0.000	0.0000
	Other specified coagulation defects	D68.8	12	0.075	10	0.085	2	0.074	24	0.079	0.9402
	Coagulation defect. unspecified	D68.9	162	1.017	103	0.879	34	1.263	299	0.986	0.2696
	Immune thrombocytopenic purpura	D69.3	0	0.000	0	0.000	0	0.000	0	0.000	0.0000
	**Immunodeficiency with predominantly antibody defects**	**D80**	0	0.000	0	0.000	0	0.000	0	0.000	0.0000

	Hereditary hypogammaglobulinemia	D80.0	1	0.006	0	0.000	0	0.000	1	0.003	0.8776
	Nonfamilial hypogammaglobulinemia	D80.1	52	0.327	16	0.137	6	0.223	74	0.244	0.0025
	Selective deficiency of immunoglobulin A [IgA]	D80.2	9	0.057	6	0.051	1	0.037	16	0.053	0.9412
	Selective deficiency of immunoglobulin G [IgG] subclasses	D80.3	14	0.088	5	0.043	3	0.111	22	0.073	0.2355
	Selective deficiency of immunoglobulin M [IgM]	D80.4	3	0.019	1	0.009	0	0.000	4	0.013	0.8430
	Immunodeficiency with increased immunoglobulin M [IgM]	D80.5	1	0.006	1	0.009	0	0.000	2	0.007	0.6187
	Antibody deficiency with near-normal immunoglobulins or with hyperimmunoglobulinemia	D80.6	1	0.006	0	0.000	0	0.000	1	0.003	0.8776
	Transient hypogammaglobulinemia of infancy	D80.7	0	0.000	0	0.000	0	0.000	0	0.000	0.0000
	Other immunodeficiencies with predominantly antibody defects	D80.8	3	0.019	3	0.026	0	0.000	6	0.020	0.9713
	Immunodeficiency with predominantly antibody defects. unspecified	D80.9	12	0.075	3	0.026	3	0.111	18	0.059	0.1352
	**Combined immunodeficiencies**	**D81**	0	0.000	0	0.000	0	0.000	0	0.000	0.0000
	Severe combined immunodeficiency [SCID] with reticular dysgenesis	D81.0	0	0.000	0	0.000	0	0.000	0	0.000	0.0000
	Severe combined immunodeficiency [SCID] with low T- and B-cell numbers	D81.1	1	0.006	0	0.000	0	0.000	1	0.003	0.8776
	Severe combined immunodeficiency [SCID] with low or normal B-cell numbers	D81.2	1	0.006	0	0.000	0	0.000	1	0.003	0.8776
	Adenosine deaminase [ADA] deficiency	D81.3	0	0.000	0	0.000	0	0.000	0	0.000	0.0000
	Adenosine deaminase deficiency. unspecified	D81.30	0	0.000	0	0.000	0	0.000	0	0.000	0.0000
	Severe combined immunodeficiency due to adenosine deaminase deficiency	D81.31	0	0.000	0	0.000	0	0.000	0	0.000	0.0000
	Adenosine deaminase 2 deficiency	D81.32	0	0.000	0	0.000	0	0.000	0	0.000	0.0000
	Other adenosine deaminase deficiency	D81.39	0	0.000	0	0.000	0	0.000	0	0.000	0.0000
	Nezelof's syndrome	D81.4	0	0.000	0	0.000	0	0.000	0	0.000	0.0000
	Purine nucleoside phosphorylase [PNP] deficiency	D81.5	0	0.000	0	0.000	0	0.000	0	0.000	0.0000
	Major histocompatibility complex class I deficiency	D81.6	0	0.000	0	0.000	0	0.000	0	0.000	0.0000
	Major histocompatibility complex class II deficiency	D81.7	0	0.000	0	0.000	0	0.000	0	0.000	0.0000
	Other combined immunodeficiencies	D81.8	5	0.031	1	0.009	0	0.000	6	0.020	0.3886
	Biotin-dependent carboxylase deficiency	D81.81	0	0.000	0	0.000	0	0.000	0	0.000	0.0000
	Biotinidase deficiency	D81.810	0	0.000	0	0.000	0	0.000	0	0.000	0.0000
	Other biotin-dependent carboxylase deficiency	D81.818	0	0.000	0	0.000	0	0.000	0	0.000	0.0000
	Biotin-dependent carboxylase deficiency. unspecified	D81.819	0	0.000	0	0.000	0	0.000	0	0.000	0.0000

	Activated Phosphoinositide 3-kinase Delta Syndrome [APDS]	D81.82	0	0.000	0	0.000	0	0.000	0	0.000	0.0000
	Other combined immunodeficiencies	D81.89	0	0.000	0	0.000	0	0.000	0	0.000	0.0000
	Combined immunodeficiency. unspecified	D81.9	9	0.057	4	0.034	4	0.149	17	0.056	0.5704
	**Immunodeficiency associated with other major defects**	**D82**	0	0.000	0	0.000	0	0.000	0	0.000	0.0000
	Immunodeficiency associated with other specified major defects	D82.8	0	0.000	0	0.000	0	0.000	0	0.000	0.0000
	Immunodeficiency associated with major defect. unspecified	D82.9	1	0.006	0	0.000	0	0.000	1	0.003	0.8776
	**Common variable immunodeficiency**	**D83**	0	0.000	0	0.000	0	0.000	0	0.000	0.0000
	Common variable immunodeficiency with predominant abnormalities of B-cell numbers and function	D83.0	0	0.000	0	0.000	0	0.000	0	0.000	0.0000
	Common variable immunodeficiency with predominant immunoregulatory T-cell disorders	D83.1	0	0.000	0	0.000	0	0.000	0	0.000	0.0000
	Common variable immunodeficiency with autoantibodies to B- or T-cells	D83.2	0	0.000	0	0.000	0	0.000	0	0.000	0.0000
	Other common variable immunodeficiencies	D83.8	4	0.025	1	0.009	2	0.074	7	0.023	0.5750
	Common variable immunodeficiency. unspecified	D83.9	18	0.113	7	0.060	4	0.149	29	0.096	0.2097
	Other immunodeficiencies	D84	0	0.000	0	0.000	0	0.000	0	0.000	0.0000
	Lymphocyte function antigen-1 [LFA-1] defect	D84.0	1	0.006	0	0.000	0	0.000	1	0.003	0.8776
	Defects in the complement system	D84.1	7	0.044	2	0.017	2	0.074	11	0.036	0.3749
	Other specified immunodeficiencies	D84.8	21	0.132	14	0.119	2	0.074	37	0.122	0.9081
	Immunodeficiency due to conditions classified elsewhere	D84.81	0	0.000	0	0.000	0	0.000	0	0.000	0.0000
	Immunodeficiency due to drugs and external causes	D84.82	0	0.000	0	0.000	0	0.000	0	0.000	0.0000
	Immunodeficiency due to drugs	D84.821	0	0.000	0	0.000	0	0.000	0	0.000	0.0000
	Immunodeficiency due to external causes	D84.822	0	0.000	0	0.000	0	0.000	0	0.000	0.0000
	Other immunodeficiencies	D84.89	0	0.000	0	0.000	0	0.000	0	0.000	0.0000
	Immunodeficiency. unspecified	D84.9	152	0.955	56	0.478	16	0.594	224	0.738	0.0000
	**Sarcoidosis**	**D86**	0	0.000	0	0.000	0	0.000	0	0.000	0.0000
	Sarcoidosis of lung	D86.0	15	0.094	8	0.068	3	0.111	26	0.086	0.5977
	Sarcoidosis of lymph nodes	D86.1	4	0.025	1	0.009	0	0.000	5	0.016	0.5750
	Sarcoidosis of lung with sarcoidosis of lymph nodes	D86.2	3	0.019	1	0.009	2	0.074	6	0.020	0.8430
	Sarcoidosis of skin	D86.3	0	0.000	1	0.009	0	0.000	1	0.003	0.8776
	Sarcoidosis of other sites	D86.8	9	0.057	1	0.009	2	0.074	12	0.040	0.0796

	Sarcoid meningitis	D86.81	0	0.000	0	0.000	0	0.000	0	0.000	0.0000
	Multiple cranial nerve palsies in sarcoidosis	D86.82	0	0.000	0	0.000	0	0.000	0	0.000	0.0000
	Sarcoid iridocyclitis	D86.83	0	0.000	0	0.000	0	0.000	0	0.000	0.0000
	Sarcoid pyelonephritis	D86.84	0	0.000	0	0.000	0	0.000	0	0.000	0.0000
	Sarcoid myocarditis	D86.85	0	0.000	0	0.000	0	0.000	0	0.000	0.0000
	Sarcoid arthropathy	D86.86	0	0.000	0	0.000	0	0.000	0	0.000	0.0000
	Sarcoid myositis	D86.87	0	0.000	0	0.000	0	0.000	0	0.000	0.0000
	Sarcoidosis of other sites	D86.89	0	0.000	0	0.000	0	0.000	0	0.000	0.0000
	Sarcoidosis. unspecified	D86.9	53	0.333	27	0.230	11	0.409	91	0.300	0.1462
	**Other disorders involving the immune mechanism. not elsewhere classified**	**D89**	0	0.000	0	0.000	0	0.000	0	0.000	0.0000
	Polyclonal hypergammaglobulinemia	D89.0	1	0.006	0	0.000	0	0.000	1	0.003	0.8776
	Cryoglobulinemia	D89.1	7	0.044	0	0.000	0	0.000	7	0.023	0.0591
	Hypergammaglobulinemia. unspecified	D89.2	4	0.025	1	0.009	2	0.074	7	0.023	0.5750
	Immune reconstitution syndrome	D89.3	2	0.013	1	0.009	0	0.000	3	0.010	0.7897
	Mast cell activation syndrome and related disorders	D89.4	0	0.000	0	0.000	0	0.000	0	0.000	0.0000
	Mast cell activation. unspecified	D89.40	0	0.000	0	0.000	0	0.000	0	0.000	0.0000
	Monoclonal mast cell activation syndrome	D89.41	0	0.000	0	0.000	0	0.000	0	0.000	0.0000
	Idiopathic mast cell activation syndrome	D89.42	0	0.000	0	0.000	0	0.000	0	0.000	0.0000
	Secondary mast cell activation	D89.43	0	0.000	0	0.000	0	0.000	0	0.000	0.0000
	Hereditary alpha tryptasemia	D89.44	0	0.000	0	0.000	0	0.000	0	0.000	0.0000
	Other mast cell activation disorder	D89.49	0	0.000	0	0.000	0	0.000	0	0.000	0.0000
	Other specified disorders involving the immune mechanism. not elsewhere classified	D89.8	24	0.151	5	0.043	2	0.074	31	0.102	0.0106
	Graft-versus-host disease	D89.81	0	0.000	0	0.000	0	0.000	0	0.000	0.0000
	Acute graft-versus-host disease	D89.810	0	0.000	0	0.000	0	0.000	0	0.000	0.0000
	Chronic graft-versus-host disease	D89.811	0	0.000	0	0.000	0	0.000	0	0.000	0.0000
	Acute on chronic graft-versus-host disease	D89.812	0	0.000	0	0.000	0	0.000	0	0.000	0.0000
	Graft-versus-host disease. unspecified	D89.813	0	0.000	0	0.000	0	0.000	0	0.000	0.0000
	Autoimmune lymphoproliferative syndrome [ALPS]	D89.82	0	0.000	0	0.000	0	0.000	0	0.000	0.0000

	Cytokine release syndrome	D89.83	0	0.000	0	0.000	0	0.000	0	0.000	0.0000
	Cytokine release syndrome. grade 1	D89.831	0	0.000	0	0.000	0	0.000	0	0.000	0.0000
	Cytokine release syndrome. grade 2	D89.832	0	0.000	0	0.000	0	0.000	0	0.000	0.0000
	Cytokine release syndrome. grade 3	D89.833	0	0.000	0	0.000	0	0.000	0	0.000	0.0000
	Cytokine release syndrome. grade 4	D89.834	0	0.000	0	0.000	0	0.000	0	0.000	0.0000
	Cytokine release syndrome. grade 5	D89.835	0	0.000	0	0.000	0	0.000	0	0.000	0.0000
	Cytokine release syndrome. grade unspecified	D89.839	0	0.000	0	0.000	0	0.000	0	0.000	0.0000
	Other specified disorders involving the immune mechanism. not elsewhere classified	D89.89	0	0.000	0	0.000	0	0.000	0	0.000	0.0000
	Disorder involving the immune mechanism. unspecified	D89.9	137	0.860	45	0.384	12	0.446	194	0.640	0.0000
**Total**			865	5.432	461	3.934	129	4.792	1455	4.797	0.0000

Supplementary Table 10: Diseases of the skin and subcutaneous tissue

Organ System	Extraintestinal Manifestations and Associated Immune Disorders	ICD-10	Crohn's Disease		Ulcerative Colitis		IBD-Unclassified		IBD-Total (CD + UC + IBDU)		Comparison
			N = 15924		N = 11718		N = 2692		N = 30334		UC vs. CD
			n		n		n		n		p value
				%		%		%		%	≤
Diseases of the skin and subcutaneous tissue											
	Atopic dermatitis	L20	0	0.000	0	0.000	0	0.000	0	0.000	0.0000
	Besnier's prurigo	L20.0	1	0.006	0	0.000	0	0.000	1	0.003	0.8776
	Other atopic dermatitis	L20.8	6	0.038	9	0.077	3	0.111	18	0.059	0.2631
	Atopic neurodermatitis	L20.81	0	0.000	0	0.000	0	0.000	0	0.000	0.0000
	Flexural eczema	L20.82	0	0.000	0	0.000	0	0.000	0	0.000	0.0000
	Infantile	L20.83	0	0.000	0	0.000	0	0.000	0	0.000	0.0000
	Intrinsic	L20.84	0	0.000	0	0.000	0	0.000	0	0.000	0.0000
	Other atopic dermatitis	L20.89	0	0.000	0	0.000	0	0.000	0	0.000	0.0000
	Atopic dermatitis. unspecified	L20.9	39	0.245	29	0.247	9	0.334	77	0.254	0.9360
	Psoriasis	L40	0	0.000	0	0.000	0	0.000	0	0.000	0.0000
	Psoriasis vulgaris	L40.0	4	0.025	1	0.009	0	0.000	5	0.016	0.5750
	Generalized pustular psoriasis	L40.1	2	0.013	2	0.017	3	0.111	7	0.023	0.8430
	Acrodermatitis continua	L40.2	0	0.000	0	0.000	0	0.000	0	0.000	0.0000
	Pustulosis palmaris et plantaris	L40.3	4	0.025	0	0.000	0	0.000	4	0.013	0.2263
	Guttate psoriasis	L40.4	10	0.063	8	0.068	2	0.074	20	0.066	0.9503
	Arthropathic psoriasis	L40.5	118	0.741	58	0.495	23	0.854	199	0.656	0.0137
	Arthropathic psoriasis. unspecified	L40.50	0	0.000	0	0.000	0	0.000	0	0.000	0.0000
	Distal interphalangeal psoriatic arthropathy	L40.51	0	0.000	0	0.000	0	0.000	0	0.000	0.0000
	Psoriatic arthritis mutilans	L40.52	0	0.000	0	0.000	0	0.000	0	0.000	0.0000
	Psoriatic spondylitis	L40.53	0	0.000	0	0.000	0	0.000	0	0.000	0.0000
	Psoriatic juvenile arthropathy	L40.54	0	0.000	0	0.000	0	0.000	0	0.000	0.0000

	**Other psoriatic arthropathy**	**L40.59**	**0**	**0.000**	**0**	**0.000**	**0**	**0.000**	**0**	**0.000**	**0.0000**
	**Other psoriasis**	**L40.8**	**18**	**0.113**	**6**	**0.051**	**4**	**0.149**	**28**	**0.092**	**0.1289**
	**Psoriasis. unspecified**	**L40.9**	**190**	**1.193**	**82**	**0.700**	**23**	**0.854**	**295**	**0.973**	**0.0001**
	**Erythema nodosum**	**L52**	**166**	**1.042**	**62**	**0.529**	**4**	**0.149**	**232**	**0.765**	**0.0000**
	**Alopecia areata**	**L63**	**0**	**0.000**	**0**	**0.000**	**0**	**0.000**	**0**	**0.000**	**0.0000**
	**Alopecia**	**L63.0**	**1**	**0.006**	**0**	**0.000**	**1**	**0.037**	**2**	**0.007**	**0.8776**
	**Alopecia universalis**	**L63.1**	**1**	**0.006**	**0**	**0.000**	**0**	**0.000**	**1**	**0.003**	**0.8776**
	**Ophiasis**	**L63.2**	**0**	**0.000**	**0**	**0.000**	**0**	**0.000**	**0**	**0.000**	**0.0000**
	**Other alopecia areata**	**L63.8**	**2**	**0.013**	**0**	**0.000**	**0**	**0.000**	**2**	**0.007**	**0.6187**
	**Alopecia areata. unspecified**	**L63.9**	**5**	**0.031**	**2**	**0.017**	**2**	**0.074**	**9**	**0.030**	**0.7207**
	**Hidradenitis suppurativa**	**L73.2**	**81**	**0.509**	**28**	**0.239**	**7**	**0.260**	**116**	**0.382**	**0.0006**
	**Other specified follicular disorders**	**L73.8**	**22**	**0.138**	**20**	**0.171**	**4**	**0.149**	**46**	**0.152**	**0.5963**
	**Vitiligo**	**L80**	**10**	**0.063**	**1**	**0.009**	**2**	**0.074**	**13**	**0.043**	**0.0536**
	**Pyoderma gangrenosum**	**L88**	**105**	**0.659**	**68**	**0.580**	**5**	**0.186**	**178**	**0.587**	**0.4553**
	**Pressure ulcer**	**L89**	**0**	**0.000**	**0**	**0.000**	**0**	**0.000**	**0**	**0.000**	**0.0000**
	**Lupus erythematosus**	**L93**	**0**	**0.000**	**0**	**0.000**	**0**	**0.000**	**0**	**0.000**	**0.0000**
	**Discoid lupus erythematosus**	**L93.0**	**29**	**0.182**	**16**	**0.137**	**3**	**0.111**	**48**	**0.158**	**0.4367**
	**Subacute cutaneous lupus erythematosus**	**L93.1**	**3**	**0.019**	**1**	**0.009**	**0**	**0.000**	**4**	**0.013**	**0.8430**
	**Other local lupus erythematosus**	**L93.2**	**4**	**0.025**	**2**	**0.017**	**0**	**0.000**	**6**	**0.020**	**0.9713**
	**Other localized connective tissue disorders**	**L94**	**0**	**0.000**	**0**	**0.000**	**0**	**0.000**	**0**	**0.000**	**0.0000**
	**Localized scleroderma [morphea]**	**L94.0**	**7**	**0.044**	**0**	**0.000**	**0**	**0.000**	**7**	**0.023**	**0.0591**
	**Linear scleroderma**	**L94.1**	**0**	**0.000**	**0**	**0.000**	**0**	**0.000**	**0**	**0.000**	**0.0000**
	**Calcinosis cutis**	**L94.2**	**1**	**0.006**	**0**	**0.000**	**0**	**0.000**	**1**	**0.003**	**0.8776**
	**Sclerodactyly**	**L94.3**	**1**	**0.006**	**0**	**0.000**	**0**	**0.000**	**1**	**0.003**	**0.8776**
	**Gottron's papules**	**L94.4**	**0**	**0.000**	**0**	**0.000**	**0**	**0.000**	**0**	**0.000**	**0.0000**
	**Poikiloderma vasculare atrophicans**	**L94.5**	**0**	**0.000**	**0**	**0.000**	**0**	**0.000**	**0**	**0.000**	**0.0000**
	**Ainhum**	**L94.6**	**0**	**0.000**	**0**	**0.000**	**0**	**0.000**	**0**	**0.000**	**0.0000**
	**Other specified localized connective tissue disorders**	**L94.8**	**1**	**0.006**	**0**	**0.000**	**0**	**0.000**	**1**	**0.003**	**0.8776**
	**Localized connective tissue disorder. unspecified**	**L94.9**	**3**	**0.019**	**3**	**0.026**	**2**	**0.074**	**8**	**0.026**	**0.9713**

	**Vasculitis limited to skin. not elsewhere classified**	**L95**	**0**	**0.000**	**0**	**0.000**	**0**	**0.000**	**0**	**0.000**	**0.0000**
	**Livedoid vasculitis**	**L95.0**	**0**	**0.000**	**0**	**0.000**	**0**	**0.000**	**0**	**0.000**	**0.0000**
	**Erythema elevatum diutinum**	**L95.1**	**0**	**0.000**	**0**	**0.000**	**0**	**0.000**	**0**	**0.000**	**0.0000**
	**Other vasculitis limited to the skin**	**L95.8**	**17**	**0.107**	**5**	**0.043**	**0**	**0.000**	**22**	**0.073**	**0.0987**
	**Vasculitis limited to the skin. unspecified**	**L95.9**	**24**	**0.151**	**8**	**0.068**	**5**	**0.186**	**37**	**0.122**	**0.0698**
	**Febrile neutrophilic dermatosis [Sweet]**	**L98.2**	**13**	**0.082**	**3**	**0.026**	**0**	**0.000**	**16**	**0.053**	**0.0967**
**Total**			**794**	**4.986**	**381**	**3.251**	**89**	**3.306**	**1264**	**4.167**	**0.0000**

Supplementary Table 11: Diseases of the nervous system

Organ System	Extraintestinal Manifestations and Associated Immune Disorders	ICD-10	Crohn's Disease		Ulcerative Colitis		IBD-Unclassified		IBD-Total (CD + UC + IBDU)		Comparison
			N = 15924		N = 11718		N = 2692		N = 30334		UC vs. CD
			n	%	n	%	n	%	n	%	p value ≤
**Diseases of the nervous system**											
	**Cavernous sinus thrombosis**	**G08**	21	0.132	6	0.051	2	0.074	29	0.096	0.0540
	**Multiple sclerosis**	G35	190	1.193	108	0.922	24	0.892	322	1.062	0.0356
	**Other acute disseminated demyelination**	G36	0	0.000	0	0.000	0	0.000	0	0.000	0.0000
	Neuromyelitis optica [Devic]	G36.0	0	0.000	2	0.017	1	0.037	3	0.010	0.3507
	Acute and subacute hemorrhagic leukoencephalitis [Hurst]	G36.1	0	0.000	0	0.000	0	0.000	0	0.000	0.0000
	Other specified acute disseminated demyelination	G36.8	0	0.000	1	0.009	0	0.000	1	0.003	0.8776
	Acute disseminated demyelination. unspecified	G36.9	0	0.000	0	0.000	0	0.000	0	0.000	0.0000
	Other demyelinating diseases of central nervous system	G37	0	0.000	0	0.000	0	0.000	0	0.000	0.0000
	Diffuse sclerosis of central nervous system	G37.0	0	0.000	0	0.000	0	0.000	0	0.000	0.0000
	Central demyelination of corpus callosum	G37.1	0	0.000	0	0.000	0	0.000	0	0.000	0.0000
	Central pontine myelinolysis	G37.2	2	0.013	3	0.026	0	0.000	5	0.016	0.7306
	Acute transverse myelitis in demyelinating disease of central nervous system	G37.3	16	0.100	8	0.068	0	0.000	24	0.079	0.4891
	Subacute necrotizing myelitis of central nervous system	G37.4	0	0.000	0	0.000	0	0.000	0	0.000	0.0000
	Concentric sclerosis [Balo] of central nervous system	G37.5	0	0.000	0	0.000	0	0.000	0	0.000	0.0000
	Other specified demyelinating diseases of central nervous system	G37.8	6	0.038	4	0.034	2	0.074	12	0.040	0.8674
	Demyelinating disease of central nervous system. unspecified	G37.9	56	0.352	33	0.282	6	0.223	95	0.313	0.3636
	**Transient cerebral ischemic attacks and related syndromes**	**G45**	0	0.000	0	0.000	0	0.000	0	0.000	0.0000
	Vertebro-basilar artery syndrome	G45.0	22	0.138	23	0.196	4	0.149	49	0.162	0.3013
	Carotid artery syndrome	G45.1	37	0.232	24	0.205	4	0.149	65	0.214	0.7244
	Multiple and bilateral precerebral artery syndromes	G45.2	1	0.006	0	0.000	0	0.000	1	0.003	0.8776

	Amaurosis fugax	G45.3	16	0.100	13	0.111	6	0.223	35	0.115	0.9382
	Transient global amnesia	G45.4	23	0.144	16	0.137	7	0.260	46	0.152	0.9915
	Other transient cerebral ischemic attacks and related syndromes	G45.8	15	0.094	7	0.060	1	0.037	23	0.076	0.4306
	Transient cerebral ischemic attack. unspecified	G45.9	325	2.041	267	2.279	71	2.637	663	2.186	0.1914
	**Vascular syndromes of brain in cerebrovascular diseases**	**G46**	0	0.000	0	0.000	0	0.000	0	0.000	0.0000
	Middle cerebral artery syndrome	G46.0	3	0.019	1	0.009	0	0.000	4	0.013	0.8430
	Anterior cerebral artery syndrome	G46.1	0	0.000	0	0.000	0	0.000	0	0.000	0.0000
	Posterior cerebral artery syndrome	G46.2	0	0.000	0	0.000	0	0.000	0	0.000	0.0000
	Brain stem stroke syndrome	G46.3	3	0.019	2	0.017	1	0.037	6	0.020	0.7306
	Cerebellar stroke syndrome	G46.4	3	0.019	7	0.060	4	0.149	14	0.046	0.1479
	Pure motor lacunar syndrome	G46.5	0	0.000	0	0.000	0	0.000	0	0.000	0.0000
	Pure sensory lacunar syndrome	G46.6	1	0.006	0	0.000	0	0.000	1	0.003	0.8776
	Other lacunar syndromes	G46.7	4	0.025	2	0.017	2	0.074	8	0.026	0.9713
	Other vascular syndromes of brain in cerebrovascular diseases	G46.8	3	0.019	2	0.017	0	0.000	5	0.016	0.7306
	**Melkersson-Rosenthal syndrome**	G51.2	0	0.000	0	0.000	0	0.000	0	0.000	0.0000
	**Postviral and related fatigue syndromes**	G93.3	13	0.082	15	0.128	6	0.223	34	0.112	0.3142
	**Myalgic encephalomyelitis/chronic fatigue syndrome**	G93.32	0	0.000	0	0.000	0	0.000	0	0.000	0.0000
**Total**			640	4.019	472	4.028	122	4.532	1234	4.068	0.9950

Supplementary Table 12: Endocrine. nutritional. and metabolic diseases

Organ System	Extraintestinal Manifestations and Associated Immune Disorders	ICD-10	Crohn's Disease		Ulcerative Colitis		IBD-Unclassified		IBD-Total (CD + UC + IBDU)		Comparison
			N = 15924		N = 11718		N = 2692		N = 30334		UC vs. CD
			n		n		n		n		p value
				%		%		%		%	≤
**Endocrine. nutritional and metabolic diseases**											
	**Thyroiditis**	**E06**	0	0.000	0	0.000	0	0.000	0	0.000	0.0000
	Acute thyroiditis	E06.0	4	0.025	1	0.009	0	0.000	5	0.016	0.5750
	Subacute thyroiditis	E06.1	7	0.044	8	0.068	1	0.037	16	0.053	0.5509
	Chronic thyroiditis with transient thyrotoxicosis	E06.2	0	0.000	0	0.000	0	0.000	0	0.000	0.0000
	Autoimmune thyroiditis	E06.3	28	0.176	16	0.137	4	0.149	48	0.158	0.5111
	Drug-induced thyroiditis	E06.4	0	0.000	1	0.009	0	0.000	1	0.003	0.8776
	Other chronic thyroiditis	E06.5	1	0.006	1	0.009	0	0.000	2	0.007	0.6187
	Thyroiditis. unspecified	E06.9	5	0.031	10	0.085	4	0.149	19	0.063	0.1007
	**Type 1 diabetes mellitus**	**E10**	0	0.000	0	0.000	0	0.000	0	0.000	0.0000
	Type 1 diabetes mellitus with ketoacidosis	E10.1	0	0.000	0	0.000	0	0.000	0	0.000	0.0000
	Type 1 diabetes mellitus with ketoacidosis without coma	E10.10	20	0.126	17	0.145	11	0.409	48	0.158	0.7862
	Type 1 diabetes mellitus with ketoacidosis with coma	E10.11	0	0.000	1	0.009	0	0.000	1	0.003	0.8776
	Type 1 diabetes mellitus with kidney complications	E10.2	0	0.000	0	0.000	0	0.000	0	0.000	0.0000
	Type 1 diabetes mellitus with diabetic nephropathy	E10.21	0	0.000	0	0.000	0	0.000	0	0.000	0.0000
	Type 1 diabetes mellitus with diabetic chronic kidney disease	E10.22	2	0.013	6	0.051	2	0.074	10	0.033	0.1313
	Type 1 diabetes mellitus with other diabetic kidney complication	E10.29	20	0.126	21	0.179	12	0.446	53	0.175	0.3239

	Type 1 diabetes mellitus with ophthalmic complications	E10.3	0	0.000	0	0.000	0	0.000	0	0.000	0.0000
	Type 1 diabetes mellitus with unspecified diabetic retinopathy	E10.31	0	0.000	0	0.000	0	0.000	0	0.000	0.0000
	Type 1 diabetes mellitus with unspecified diabetic retinopathy with macular edema	E10.311	0	0.000	0	0.000	0	0.000	0	0.000	0.0000
	Type 1 diabetes mellitus with unspecified diabetic retinopathy without macular edema	E10.319	0	0.000	0	0.000	0	0.000	0	0.000	0.0000
	Type 1 diabetes mellitus with mild nonproliferative diabetic retinopathy	E10.32	6	0.038	13	0.111	2	0.074	21	0.069	0.0390
	Type 1 diabetes mellitus with mild nonproliferative diabetic retinopathy with macular edema	E10.321	0	0.000	0	0.000	0	0.000	0	0.000	0.0000
	Type 1 diabetes mellitus with mild nonproliferative diabetic retinopathy without macular edema	E10.329	1	0.006	2	0.017	0	0.000	3	0.010	0.7897
	Type 1 diabetes mellitus with moderate nonproliferative diabetic retinopathy	E10.33	8	0.050	8	0.068	4	0.149	20	0.066	0.7166
	Type 1 diabetes mellitus with moderate nonproliferative diabetic retinopathy with macular edema	E10.331	0	0.000	0	0.000	0	0.000	0	0.000	0.0000
	Type 1 diabetes mellitus with moderate nonproliferative diabetic retinopathy without macular edema	E10.339	0	0.000	0	0.000	0	0.000	0	0.000	0.0000
	Type 1 diabetes mellitus with severe nonproliferative diabetic retinopathy	E10.34	0	0.000	0	0.000	0	0.000	0	0.000	0.0000
	Type 1 diabetes mellitus with severe nonproliferative diabetic retinopathy with macular edema	E10.341	0	0.000	0	0.000	0	0.000	0	0.000	0.0000
	Type 1 diabetes mellitus with severe nonproliferative diabetic retinopathy without macular edema	E10.349	0	0.000	0	0.000	0	0.000	0	0.000	0.0000
	Type 1 diabetes mellitus with proliferative diabetic retinopathy	E10.35	0	0.000	1	0.009	0	0.000	1	0.003	0.8776
	Type 1 diabetes mellitus with proliferative diabetic retinopathy with macular edema	E10.351	0	0.000	0	0.000	0	0.000	0	0.000	0.0000
	Type 1 diabetes mellitus with proliferative diabetic retinopathy with traction retinal detachment involving the macula	E10.352	0	0.000	0	0.000	0	0.000	0	0.000	0.0000
	Type 1 diabetes mellitus with proliferative diabetic retinopathy with traction retinal detachment not involving the macula	E10.353	0	0.000	0	0.000	0	0.000	0	0.000	0.0000
	Type 1 diabetes mellitus with proliferative diabetic retinopathy with combined traction retinal detachment and rhegmatogenous retinal detachment	E10.354	0	0.000	0	0.000	0	0.000	0	0.000	0.0000
	Type 1 diabetes mellitus with stable proliferative diabetic retinopathy	E10.355	0	0.000	0	0.000	0	0.000	0	0.000	0.0000
	Type 1 diabetes mellitus with proliferative diabetic retinopathy without macular edema	E10.359	0	0.000	0	0.000	0	0.000	0	0.000	0.0000
	Type 1 diabetes mellitus with diabetic cataract	E10.36	0	0.000	0	0.000	0	0.000	0	0.000	0.0000
	Type 1 diabetes mellitus with diabetic macular edema. resolved following treatment	E10.37	0	0.000	0	0.000	0	0.000	0	0.000	0.0000
	Type 1 diabetes mellitus with other diabetic ophthalmic complication	E10.39	0	0.000	0	0.000	0	0.000	0	0.000	0.0000

	Type 1 diabetes mellitus with neurological complications	E10.4	0	0.000	0	0.000	0	0.000	0	0.000	0.0000
	Type 1 diabetes mellitus with diabetic neuropathy. unspecified	E10.40	1	0.006	2	0.017	3	0.111	6	0.020	0.7897
	Type 1 diabetes mellitus with diabetic mononeuropathy	E10.41	15	0.094	12	0.102	7	0.260	34	0.112	0.9832
	Type 1 diabetes mellitus with diabetic polyneuropathy	E10.42	7	0.044	6	0.051	9	0.334	22	0.073	0.9951
	Type 1 diabetes mellitus with diabetic autonomic	E10.43	0	0.000	0	0.000	0	0.000	0	0.000	0.0000
	Type 1 diabetes mellitus with diabetic amyotrophy	E10.44	0	0.000	0	0.000	0	0.000	0	0.000	0.0000
	Type 1 diabetes mellitus with other diabetic neurological complication	E10.49	0	0.000	0	0.000	0	0.000	0	0.000	0.0000
	Type 1 diabetes mellitus with circulatory complications	E10.5	0	0.000	0	0.000	0	0.000	0	0.000	0.0000
	Type 1 diabetes mellitus with diabetic peripheral angiopathy without gangrene	E10.51	0	0.000	1	0.009	0	0.000	1	0.003	0.8776
	Type 1 diabetes mellitus with diabetic peripheral angiopathy with gangrene	E10.52	54	0.339	45	0.384	20	0.743	119	0.392	0.6060
	Type 1 diabetes mellitus with other circulatory complications	E10.59	0	0.000	0	0.000	0	0.000	0	0.000	0.0000
	Type 1 diabetes mellitus with other specified complications	E10.6	0	0.000	0	0.000	0	0.000	0	0.000	0.0000
	Type 1 diabetes mellitus with diabetic arthropathy	E10.61	2	0.013	1	0.009	2	0.074	5	0.016	0.7897
	Type 1 diabetes mellitus with diabetic neuropathic arthropathy	E10.610	0	0.000	0	0.000	0	0.000	0	0.000	0.0000
	Type 1 diabetes mellitus with other diabetic arthropathy	E10.618	0	0.000	0	0.000	0	0.000	0	0.000	0.0000
	Type 1 diabetes mellitus with skin complications	E10.62	0	0.000	0	0.000	0	0.000	0	0.000	0.0000
	Type 1 diabetes mellitus with diabetic dermatitis	E10.620	0	0.000	0	0.000	0	0.000	0	0.000	0.0000
	Type 1 diabetes mellitus with foot ulcer	E10.621	0	0.000	0	0.000	0	0.000	0	0.000	0.0000
	Type 1 diabetes mellitus with other skin ulcer	E10.622	0	0.000	0	0.000	0	0.000	0	0.000	0.0000
	Type 1 diabetes mellitus with other skin complications	E10.628	0	0.000	0	0.000	0	0.000	0	0.000	0.0000
	Type 1 diabetes mellitus with oral complications	E10.63	28	0.176	26	0.222	10	0.371	64	0.211	0.4722
	Type 1 diabetes mellitus with periodontal disease	E10.630	0	0.000	0	0.000	0	0.000	0	0.000	0.0000
	Type 1 diabetes mellitus with other oral complications	E10.638	0	0.000	0	0.000	0	0.000	0	0.000	0.0000
	Type 1 diabetes mellitus with hypoglycemia	E10.64	34	0.214	23	0.19	15	0.557	72	0.237	0.8587

						6					
	Type 1 diabetes mellitus with hypoglycemia with coma	E10.641	0	0.000	0	0.000	0	0.000	0	0.000	0.0000
	Type 1 diabetes mellitus with hypoglycemia without coma	E10.649	0	0.000	0	0.000	0	0.000	0	0.000	0.0000
	Type 1 diabetes mellitus with hyperglycemia	E10.65	0	0.000	0	0.000	0	0.000	0	0.000	0.0000
	Type 1 diabetes mellitus with other specified complication	E10.69	0	0.000	0	0.000	0	0.000	0	0.000	0.0000
	Type 1 diabetes mellitus with unspecified complications	E10.8	0	0.000	0	0.000	0	0.000	0	0.000	0.0000
	Type 1 diabetes mellitus without complications	E10.9	243	1.526	205	1.749	66	2.452	514	1.694	0.1598
	**Polyglandular dysfunction**	**E31**	0	0.000	0	0.000	0	0.000	0	0.000	0.0000
	Autoimmune polyglandular failure	E31.0	1	0.006	4	0.034	0	0.000	5	0.016	0.2115
	Polyglandular hyperfunction	E31.1	0	0.000	0	0.000	0	0.000	0	0.000	0.0000
	Multiple endocrine neoplasia [MEN] syndromes	E31.2	0	0.000	0	0.000	0	0.000	0	0.000	0.0000
	Multiple endocrine neoplasia [MEN] syndrome. unspecified	E31.20	0	0.000	0	0.000	0	0.000	0	0.000	0.0000
	Multiple endocrine neoplasia [MEN] type I	E31.21	0	0.000	0	0.000	0	0.000	0	0.000	0.0000
	Multiple endocrine neoplasia [MEN] type IIA	E31.22	0	0.000	0	0.000	0	0.000	0	0.000	0.0000
	Multiple endocrine neoplasia [MEN] type IIB	E31.23	0	0.000	0	0.000	0	0.000	0	0.000	0.0000
	Other polyglandular dysfunction	E31.8	0	0.000	0	0.000	0	0.000	0	0.000	0.0000
	Polyglandular dysfunction. unspecified	E31.9	0	0.000	0	0.000	0	0.000	0	0.000	0.0000
	Amyloidosis	**E85**	0	0.000	0	0.000	0	0.000	0	0.000	0.0000
	Non-neuropathic heredofamilial amyloidosis	E85.0	2	0.013	0	0.000	0	0.000	2	0.007	0.6187
	Neuropathic heredofamilial amyloidosis	E85.1	0	0.000	0	0.000	0	0.000	0	0.000	0.0000
	Heredofamilial amyloidosis. unspecified	E85.2	0	0.000	0	0.000	0	0.000	0	0.000	0.0000
	Secondary systemic amyloidosis	E85.3	0	0.000	1	0.009	0	0.000	1	0.003	0.8776
	Organ-limited amyloidosis	E85.4	4	0.025	5	0.043	2	0.074	11	0.036	0.6441
	Other amyloidosis	E85.8	2	0.013	3	0.026	0	0.000	5	0.016	0.7306

	Light chain	E85.81	0	0.000	0	0.000	0	0.000	0	0.000	0.0000
	Wild-type transthyretin-related	E85.82	0	0.000	0	0.000	0	0.000	0	0.000	0.0000
	Other amyloidosis	E85.89	0	0.000	0	0.000	0	0.000	0	0.000	0.0000
	Amyloidosis. unspecified	E85.9	5	0.031	5	0.043	0	0.000	10	0.033	0.8674
**Total**			327	2.054	286	2.441	94	3.492	707	2.331	0.0341

Supplementary Table 13: Diseases of the eye and adnexa

Organ System	Extraintestinal Manifestations and Associated Immune Disorders	ICD-10	Crohn's Disease		Ulcerative Colitis		IBD-Unclassified		IBD-Total (CD + UC + IBDU)		Comparison
			N = 15924		N = 11718		N = 2692		N = 30334		UC vs. CD
			n		n		n		n		p value
				%		%		%		%	≤
**Diseases of the eye and adnexa**											
	**Disorders of sclera**	H15	0	0.000	0	0.000	0	0.000	0	0.000	0.0000
	Scleritis	H15.0	51	0.320	26	0.222	3	0.111	80	0.264	0.1561
	Unspecified scleritis	H15.00	0	0.000	0	0.000	0	0.000	0	0.000	0.0000
	Unspecified scleritis. right eye	H15.001	0	0.000	0	0.000	0	0.000	0	0.000	0.0000
	Unspecified scleritis. left eye	H15.002	0	0.000	0	0.000	0	0.000	0	0.000	0.0000
	Unspecified scleritis. bilateral	H15.003	0	0.000	0	0.000	0	0.000	0	0.000	0.0000
	Unspecified scleritis. unspecified eye	H15.009	0	0.000	0	0.000	0	0.000	0	0.000	0.0000
	Anterior scleritis	H15.01	0	0.000	0	0.000	0	0.000	0	0.000	0.0000
	Anterior scleritis. right eye	H15.011	0	0.000	0	0.000	0	0.000	0	0.000	0.0000
	Anterior scleritis. left eye	H15.012	0	0.000	0	0.000	0	0.000	0	0.000	0.0000
	Anterior scleritis. bilateral	H15.013	0	0.000	0	0.000	0	0.000	0	0.000	0.0000
	Anterior scleritis. unspecified eye	H15.019	0	0.000	0	0.000	0	0.000	0	0.000	0.0000
	Brawny scleritis	H15.02	0	0.000	0	0.000	0	0.000	0	0.000	0.0000
	Brawny scleritis. right eye	H15.021	0	0.000	0	0.000	0	0.000	0	0.000	0.0000
	Brawny scleritis. left eye	H15.022	0	0.000	0	0.000	0	0.000	0	0.000	0.0000
	Brawny scleritis. bilateral	H15.023	0	0.000	0	0.000	0	0.000	0	0.000	0.0000
	Brawny scleritis. unspecified eye	H15.029	0	0.000	0	0.000	0	0.000	0	0.000	0.0000
	Posterior scleritis	H15.03	0	0.000	0	0.000	0	0.000	0	0.000	0.0000
	Posterior scleritis. right eye	H15.031	0	0.000	0	0.000	0	0.000	0	0.000	0.0000
	Posterior scleritis. left eye	H15.032	0	0.000	0	0.000	0	0.000	0	0.000	0.0000

	Posterior scleritis. bilateral	H15.033	0	0.000	0	0.000	0	0.000	0	0.000	0.0000
	Posterior scleritis. unspecified eye	H15.039	0	0.000	0	0.000	0	0.000	0	0.000	0.0000
	Scleritis with corneal involvement	H15.04	0	0.000	0	0.000	0	0.000	0	0.000	0.0000
	Scleritis with corneal involvement. right eye	H15.041	0	0.000	0	0.000	0	0.000	0	0.000	0.0000
	Scleritis with corneal involvement. left eye	H15.042	0	0.000	0	0.000	0	0.000	0	0.000	0.0000
	Scleritis with corneal involvement. bilateral	H15.043	0	0.000	0	0.000	0	0.000	0	0.000	0.0000
	Scleritis with corneal involvement. unspecified eye	H15.049	0	0.000	0	0.000	0	0.000	0	0.000	0.0000
	Scleromalacia perforans	H15.05	0	0.000	0	0.000	0	0.000	0	0.000	0.0000
	Scleromalacia perforans. right eye	H15.051	0	0.000	0	0.000	0	0.000	0	0.000	0.0000
	Scleromalacia perforans. left eye	H15.052	0	0.000	0	0.000	0	0.000	0	0.000	0.0000
	Scleromalacia perforans. bilateral	H15.053	0	0.000	0	0.000	0	0.000	0	0.000	0.0000
	Scleromalacia perforans. unspecified eye	H15.059	0	0.000	0	0.000	0	0.000	0	0.000	0.0000
	Other scleritis	H15.09	0	0.000	0	0.000	0	0.000	0	0.000	0.0000
	Other scleritis. right eye	H15.091	0	0.000	0	0.000	0	0.000	0	0.000	0.0000
	Other scleritis. left eye	H15.092	0	0.000	0	0.000	0	0.000	0	0.000	0.0000
	Other scleritis. bilateral	H15.093	0	0.000	0	0.000	0	0.000	0	0.000	0.0000
	Other scleritis. unspecified eye	H15.099	0	0.000	0	0.000	0	0.000	0	0.000	0.0000
	Episcleritis	H15.1	56	0.352	26	0.222	4	0.149	86	0.284	0.0645
	Unspecified episcleritis	H15.10	0	0.000	0	0.000	0	0.000	0	0.000	0.0000
	Unspecified episcleritis. right eye	H15.101	0	0.000	0	0.000	0	0.000	0	0.000	0.0000
	Unspecified episcleritis. left eye	H15.102	0	0.000	0	0.000	0	0.000	0	0.000	0.0000
	Unspecified episcleritis. bilateral	H15.103	0	0.000	0	0.000	0	0.000	0	0.000	0.0000
	Unspecified episcleritis. unspecified eye	H15.109	0	0.000	0	0.000	0	0.000	0	0.000	0.0000
	Episcleritis periodica fugax	H15.11	0	0.000	0	0.000	0	0.000	0	0.000	0.0000
	Episcleritis periodica fugax. right eye	H15.111	0	0.000	0	0.000	0	0.000	0	0.000	0.0000
	Episcleritis periodica fugax. left eye	H15.112	0	0.000	0	0.000	0	0.000	0	0.000	0.0000
	Episcleritis periodica fugax. bilateral	H15.113	0	0.000	0	0.000	0	0.000	0	0.000	0.0000
	Episcleritis periodica fugax. unspecified eye	H15.119	0	0.000	0	0.000	0	0.000	0	0.000	0.0000
	Nodular episcleritis	H15.12	0	0.000	0	0.000	0	0.000	0	0.000	0.0000

	Nodular episcleritis. right eye	H15.121	0	0.000	0	0.000	0	0.000	0	0.000	0.0000
	Nodular episcleritis. left eye	H15.122	0	0.000	0	0.000	0	0.000	0	0.000	0.0000
	Nodular episcleritis. bilateral	H15.123	0	0.000	0	0.000	0	0.000	0	0.000	0.0000
	Nodular episcleritis. unspecified eye	H15.129	0	0.000	0	0.000	0	0.000	0	0.000	0.0000
	Interstitial and deep keratitis	H16.3	3	0.019	1	0.009	0	0.000	4	0.013	0.8430
	Unspecified interstitial keratitis	H16.30	0	0.000	0	0.000	0	0.000	0	0.000	0.0000
	Unspecified interstitial keratitis. right eye	H16.301	0	0.000	0	0.000	0	0.000	0	0.000	0.0000
	Unspecified interstitial keratitis. left eye	H16.302	0	0.000	0	0.000	0	0.000	0	0.000	0.0000
	Unspecified interstitial keratitis. bilateral	H16.303	0	0.000	0	0.000	0	0.000	0	0.000	0.0000
	Unspecified interstitial keratitis. unspecified eye	H16.309	0	0.000	0	0.000	0	0.000	0	0.000	0.0000
	Diffuse interstitial keratitis	H16.32	0	0.000	0	0.000	0	0.000	0	0.000	0.0000
	Diffuse interstitial keratitis. right eye	H16.321	0	0.000	0	0.000	0	0.000	0	0.000	0.0000
	Diffuse interstitial keratitis. left eye	H16.322	0	0.000	0	0.000	0	0.000	0	0.000	0.0000
	Diffuse interstitial keratitis. bilateral	H16.323	0	0.000	0	0.000	0	0.000	0	0.000	0.0000
	Diffuse interstitial keratitis. unspecified eye	H16.329	0	0.000	0	0.000	0	0.000	0	0.000	0.0000
	Sclerosing keratitis	H16.33	0	0.000	0	0.000	0	0.000	0	0.000	0.0000
	Sclerosing keratitis. right eye	H16.331	0	0.000	0	0.000	0	0.000	0	0.000	0.0000
	Sclerosing keratitis. left eye	H16.332	0	0.000	0	0.000	0	0.000	0	0.000	0.0000
	Sclerosing keratitis. bilateral	H16.333	0	0.000	0	0.000	0	0.000	0	0.000	0.0000
	Sclerosing keratitis. unspecified eye	H16.339	0	0.000	0	0.000	0	0.000	0	0.000	0.0000
	Other interstitial and deep keratitis	H16.39	0	0.000	0	0.000	0	0.000	0	0.000	0.0000
	Other interstitial and deep keratitis. right eye	H16.391	0	0.000	0	0.000	0	0.000	0	0.000	0.0000
	Other interstitial and deep keratitis. left eye	H16.392	0	0.000	0	0.000	0	0.000	0	0.000	0.0000
	Other interstitial and deep keratitis. bilateral	H16.393	0	0.000	0	0.000	0	0.000	0	0.000	0.0000
	Other interstitial and deep keratitis. unspecified eye	H16.399	0	0.000	0	0.000	0	0.000	0	0.000	0.0000
	**Iridocyclitis**	**H20**	0	0.000	0	0.000	0	0.000	0	0.000	0.0000
	Acute and subacute iridocyclitis	H20.0	29	0.182	15	0.128	1	0.037	45	0.148	0.3358
	Unspecified acute and subacute iridocyclitis	H20.00	0	0.000	0	0.000	0	0.000	0	0.000	0.0000
	Primary iridocyclitis	H20.01	0	0.000	0	0.000	0	0.000	0	0.000	0.0000

	Primary iridocyclitis. right eye	H20.011	0	0.000	0	0.000	0	0.000	0	0.000	0.0000
	Primary iridocyclitis. left eye	H20.012	0	0.000	0	0.000	0	0.000	0	0.000	0.0000
	Primary iridocyclitis. bilateral	H20.013	0	0.000	0	0.000	0	0.000	0	0.000	0.0000
	Primary iridocyclitis. unspecified eye	H20.019	0	0.000	0	0.000	0	0.000	0	0.000	0.0000
	Recurrent acute iridocyclitis	H20.02	0	0.000	0	0.000	0	0.000	0	0.000	0.0000
	Recurrent acute iridocyclitis. right eye	H20.021	0	0.000	0	0.000	0	0.000	0	0.000	0.0000
	Recurrent acute iridocyclitis. left eye	H20.022	0	0.000	0	0.000	0	0.000	0	0.000	0.0000
	Recurrent acute iridocyclitis. bilateral	H20.023	0	0.000	0	0.000	0	0.000	0	0.000	0.0000
	Recurrent acute iridocyclitis. unspecified eye	H20.029	0	0.000	0	0.000	0	0.000	0	0.000	0.0000
	Secondary infectious iridocyclitis	H20.03	0	0.000	0	0.000	0	0.000	0	0.000	0.0000
	Secondary infectious iridocyclitis. right eye	H20.031	0	0.000	0	0.000	0	0.000	0	0.000	0.0000
	Secondary infectious iridocyclitis. left eye	H20.032	0	0.000	0	0.000	0	0.000	0	0.000	0.0000
	Secondary infectious iridocyclitis. bilateral	H20.033	0	0.000	0	0.000	0	0.000	0	0.000	0.0000
	Secondary infectious iridocyclitis. unspecified eye	H20.039	0	0.000	0	0.000	0	0.000	0	0.000	0.0000
	Secondary noninfectious iridocyclitis	H20.04	0	0.000	0	0.000	0	0.000	0	0.000	0.0000
	Secondary noninfectious iridocyclitis. right eye	H20.041	0	0.000	0	0.000	0	0.000	0	0.000	0.0000
	Secondary noninfectious iridocyclitis. left eye	H20.042	0	0.000	0	0.000	0	0.000	0	0.000	0.0000
	Secondary noninfectious iridocyclitis. bilateral	H20.043	0	0.000	0	0.000	0	0.000	0	0.000	0.0000
	Secondary noninfectious iridocyclitis. unspecified eye	H20.049	0	0.000	0	0.000	0	0.000	0	0.000	0.0000
	Hypopyon	H20.05	0	0.000	0	0.000	0	0.000	0	0.000	0.0000
	Hypopyon. right eye	H20.051	0	0.000	0	0.000	0	0.000	0	0.000	0.0000
	Hypopyon. left eye	H20.052	0	0.000	0	0.000	0	0.000	0	0.000	0.0000
	Hypopyon. bilateral	H20.053	0	0.000	0	0.000	0	0.000	0	0.000	0.0000
	Hypopyon. unspecified eye	H20.059	0	0.000	0	0.000	0	0.000	0	0.000	0.0000
	Chronic iridocyclitis	H20.1	5	0.031	2	0.017	0	0.000	7	0.023	0.7207
	Chronic iridocyclitis. unspecified eye	H20.10	0	0.000	0	0.000	0	0.000	0	0.000	0.0000
	Chronic iridocyclitis. right eye	H20.11	0	0.000	0	0.000	0	0.000	0	0.000	0.0000
	Chronic iridocyclitis. left eye	H20.12	0	0.000	0	0.000	0	0.000	0	0.000	0.0000
	Chronic iridocyclitis. bilateral	H20.13	0	0.000	0	0.000	0	0.000	0	0.000	0.0000

	Lens-induced iridocyclitis	H20.2	0	0.000	0	0.000	0	0.000	0	0.000	0.0000
	Lens-induced iridocyclitis. unspecified eye	H20.20	0	0.000	0	0.000	0	0.000	0	0.000	0.0000
	Lens-induced iridocyclitis. right eye	H20.21	0	0.000	0	0.000	0	0.000	0	0.000	0.0000
	Lens-induced iridocyclitis. left eye	H20.22	0	0.000	0	0.000	0	0.000	0	0.000	0.0000
	Lens-induced iridocyclitis. bilateral	H20.23	0	0.000	0	0.000	0	0.000	0	0.000	0.0000
	Other iridocyclitis	H20.8	11	0.069	2	0.017	2	0.074	15	0.049	0.0910
	Fuchs' heterochromic cyclitis	H20.81	0	0.000	0	0.000	0	0.000	0	0.000	0.0000
	Fuchs' heterochromic cyclitis. right eye	H20.811	0	0.000	0	0.000	0	0.000	0	0.000	0.0000
	Fuchs' heterochromic cyclitis. left eye	H20.812	0	0.000	0	0.000	0	0.000	0	0.000	0.0000
	Fuchs' heterochromic cyclitis. bilateral	H20.813	0	0.000	0	0.000	0	0.000	0	0.000	0.0000
	Fuchs' heterochromic cyclitis. unspecified eye	H20.819	0	0.000	0	0.000	0	0.000	0	0.000	0.0000
	Vogt-Koyanagi syndrome	H20.82	0	0.000	0	0.000	0	0.000	0	0.000	0.0000
	Vogt-Koyanagi syndrome. right eye	H20.821	0	0.000	0	0.000	0	0.000	0	0.000	0.0000
	Vogt-Koyanagi syndrome. left eye	H20.822	0	0.000	0	0.000	0	0.000	0	0.000	0.0000
	Vogt-Koyanagi syndrome. bilateral	H20.823	0	0.000	0	0.000	0	0.000	0	0.000	0.0000
	Vogt-Koyanagi syndrome. unspecified eye	H20.829	0	0.000	0	0.000	0	0.000	0	0.000	0.0000
	Unspecified iridocyclitis	H20.9	377	2.367	172	1.468	32	1.189	581	1.915	0.0000
**Total**			458	2.876	212	1.809	37	1.374	707	2.331	0.0000

Supplementary Table 14: Diseases of the ear and mastoid process

Organ System	Extraintestinal Manifestations and Associated Immune Disorders	ICD-10	Crohn's Disease		Ulcerative Colitis		IBD-Unclassified		IBD-Total (CD + UC + IBDU)		Comparison
			N = 15924		N = 11718		N = 2692		N = 30334		UC vs. CD
			n	%	n	%	n	%	n	%	p value ≤
**Diseases of the ear and mastoid process**											
	**Conductive and sensorineural hearing loss**	**H90**	0	0.000	0	0.000	0	0.000	0	0.000	0.0000
	Sensorineural hearing loss. bilateral	H90.3	109	0.685	90	0.768	26	0.966	225	0.742	0.4593
	Sensorineural hearing loss. unilateral with unrestricted hearing on the contralateral side	H90.4	33	0.207	35	0.299	8	0.297	76	0.251	0.1633
	Sensorineural hearing loss. unilateral. right ear. with unrestricted hearing on the contralateral side	H90.41	0	0.000	0	0.000	0	0.000	0	0.000	0.0000
	Sensorineural hearing loss. unilateral. left ear. with unrestricted hearing on the contralateral side	H90.42	0	0.000	0	0.000	0	0.000	0	0.000	0.0000
	Unspecified sensorineural hearing loss	H90.5	99	0.622	91	0.777	12	0.446	202	0.666	0.1425
	Mixed conductive and sensorineural hearing loss. bilateral	H90.6	15	0.094	15	0.128	2	0.074	32	0.105	0.5100
	Mixed conductive and sensorineural hearing loss. unilateral with unrestricted hearing on the contralateral side	H90.7	10	0.063	10	0.085	1	0.037	21	0.069	0.6438
	Mixed conductive and sensorineural hearing loss. unilateral. right ear. with unrestricted hearing on the contralateral side	H90.71	0	0.000	0	0.000	0	0.000	0	0.000	0.0000
	Mixed conductive and sensorineural hearing loss. unilateral. left ear. with unrestricted hearing on the contralateral side	H90.72	0	0.000	0	0.000	0	0.000	0	0.000	0.0000
	Mixed conductive and sensorineural hearing loss. unspecified	H90.8	10	0.063	10	0.085	2	0.074	22	0.073	0.6438
	Conductive and sensorineural hearing loss with restricted hearing on the contralateral side	H90.A	0	0.000	0	0.000	0	0.000	0	0.000	0.0000
	Conductive hearing loss. unilateral. with restricted hearing on the contralateral side	H90.A1	0	0.000	0	0.000	0	0.000	0	0.000	0.0000
	Conductive hearing loss. unilateral. right ear with restricted hearing on the contralateral side	H90.A11	0	0.000	0	0.000	0	0.000	0	0.000	0.0000
	Conductive hearing loss. unilateral. left ear with restricted hearing on the contralateral side	H90.A12	0	0.000	0	0.000	0	0.000	0	0.000	0.0000
	Sensorineural hearing loss. unilateral. with restricted hearing on the contralateral side	H90.A2	0	0.000	0	0.000	0	0.000	0	0.000	0.0000
	Sensorineural hearing loss. unilateral. right ear. with restricted hearing on the contralateral side	H90.A21	0	0.000	0	0.000	0	0.000	0	0.000	0.0000

	Sensorineural hearing loss. unilateral. left ear. with restricted hearing on the contralateral side	H90.A22	0	0.000	0	0.000	0	0.000	0	0.000	0.0000
	Mixed conductive and sensorineural hearing loss. unilateral with restricted hearing on the contralateral side	H90.A3	0	0.000	0	0.000	0	0.000	0	0.000	0.0000
	Mixed conductive and sensorineural hearing loss. unilateral. right ear with restricted hearing on the contralateral side	H90.A31	0	0.000	0	0.000	0	0.000	0	0.000	0.0000
	Mixed conductive and sensorineural hearing loss. unilateral. left ear with restricted hearing on the contralateral side	H90.A32	0	0.000	0	0.000	0	0.000	0	0.000	0.0000
**Total**			244	1.532	215	1.835	48	1.783	507	1.671	0.0578
